# Comparative efficacy and acceptability of licensed dose intranasal corticosteroids for moderate-to-severe allergic rhinitis: a systematic review and network meta-analysis

**DOI:** 10.3389/fphar.2023.1184552

**Published:** 2023-05-23

**Authors:** Kay Khine Soe, Thanachit Krikeerati, Chatkamol Pheerapanyawaranun, Suvimol Niyomnaitham, Phichayut Phinyo, Torpong Thongngarm

**Affiliations:** ^1^ Department of Pharmacology, Faculty of Medicine Siriraj Hospital, Mahidol University, Bangkok, Thailand; ^2^ Division of Allergy and Clinical Immunology, Department of Medicine, Faculty of Medicine Siriraj Hospital, Mahidol University, Bangkok, Thailand; ^3^ Siriraj Institute of Clinical Research, Faculty of Medicine Siriraj Hospital, Mahidol University, Bangkok, Thailand; ^4^ Center for Clinical Epidemiology and Clinical Statistics, Faculty of Medicine, Chiang Mai University, Chiang Mai, Thailand; ^5^ Department of Family Medicine, Faculty of Medicine, Chiang Mai University, Chiang Mai, Thailand; ^6^ Musculoskeletal Science and Translational Research (MSTR), Chiang Mai University, Chiang Mai, Thailand

**Keywords:** acceptability, allergic rhinitis, efficacy, intranasal corticosteroid, meta-analysis, systematic review, total nasal symptom score, total ocular symptom score

## Abstract

No evidence shows that one intranasal corticosteroid (INCS) is better than another for treating moderate-to-severe allergic rhinitis (AR). This network meta-analysis assessed the comparative efficacy and acceptability of licensed dose aqueous INCSs. PubMed/MEDLINE, Scopus, EMBASE, and the Cochrane Central Register of Controlled Trials were searched until 31 March 2022. Eligible studies included randomized controlled trials comparing INCSs with placebo or other types of INCSs in patients with moderate-to-severe allergic rhinitis. Two reviewers independently screened and extracted data following the Preferred Reporting Items in Systematic Reviews and Meta-analysis guideline. A random-effects model was used for data pooling. Continuous outcomes were expressed as standardized mean difference (SMD). The primary outcomes were the efficacy in improving total nasal symptom score (TNSS) and treatment acceptability (the study dropout). We included 26 studies, 13 with 5,134 seasonal AR patients and 13 with 4,393 perennial AR patients. Most placebo-controlled studies had a moderate quality of evidence. In seasonal AR, mometasone furoate (MF) was ranked the highest efficacy, followed by fluticasone furoate (FF), ciclesonide (CIC), fluticasone propionate and triamcinolone acetonide (TAA) (SMD −0.47, 95% CI: −0.63 to −0.31; −0.46, 95% CI: −0.59 to −0.33; −0.44, 95% CI: −0.75 to −0.13; −0.42, 95% CI: −0.67 to −0.17 and −0.41, 95% CI: −0.81 to −0.00), In perennial AR, budesonide was ranked the highest efficacy, followed by FF, TAA, CIC, and MF (SMD −0.43, 95% CI: −0.75 to −0.11; −0.36, 95% CI: −0.53 to −0.19; −0.32, 95% CI: −0.54 to −0.10; −0.29, 95% CI: −0.48 to −0.11; and −0.28, 95% CI: −0.55 to −0.01). The acceptability of all included INCSs was not inferior to the placebo. According to our indirect comparison, some INCSs have superior efficacy to others with moderate quality of evidence in most placebo-controlled studies for treating moderate-to-severe AR.

## 1 Introduction

The introduction of intranasal corticosteroid (INCS) spray in the early 1970s (Mygind, 1973) was a crucial advanced step for treating allergic rhinitis (AR). INCS is more effective than antihistamines, both oral and intranasal routes, and anti-leukotrienes and is currently the mainstay of treatment in patients with moderate-to-severe AR in both children and adults (Bousquet et al., 2020; Dykewicz et al., 2020).

Eight INCS in an aqueous nasal spray are approved for AR management: beclomethasone dipropionate (BDP), budesonide (BUD), flunisolide, triamcinolone acetonide (TAA), ciclesonide (CIC), fluticasone propionate (FP), mometasone furoate (MF), and fluticasone furoate (FF). Newer INCS, including FF, MF, and FP, have higher glucocorticoid receptor (GR) binding affinities and very low systemic bioavailability compared to older ones, such as BDP, BUD, and TAA (Derendorf and Meltzer, 2008). Although the pharmacological profiles of newer agents are close to desired criteria of an ideal INCS, well-designed head-to-head randomized controlled trials (RCTs) comparing the efficacy among INCSs are limited. Moreover, differences in types and severity of AR population, study duration, and outcome assessment of those RCTs also hamper the comparison among INCSs.

Therefore, identifying the preferred INCS with the most remarkable efficacy remains challenging. This systematic review (SR) and network meta-analysis (NMA) aims to assess the comparative efficacy and treatment acceptability (the study dropout) across all licensed-dose aqueous INCSs for moderate-to-severe AR.

## 2 Materials and methods

We followed the Cochrane Handbook for Systematic Reviews of Interventions version 6.0 (Chaimani et al., 2019) in conducting this SR and NMA. The reporting of this review complied with the Preferred Reporting Items for Systematic Reviews and Meta-analyses statement extension for NMA (Hutton et al., 2015). The review protocol was registered in the International prospective register of systematic reviews (PROSPERO CRD42022336687).

### 2.1 Eligibility criteria

The study inclusion criteria comprised: 1) RCTs; 2) Participants: patients of all ages with moderate-to-severe AR, defined by the baseline total nasal symptom score (TNSS) of at least 6 of 0–12 scale, 3) Intervetion: a licensed dose of aqueous INCSs for at least 2 weeks for seasonal AR (SAR) and at least 4 weeks for perennial AR (PAR) (United States Department of Health and Human Services Food and Drug Administration Center for Drug Evaluation and Research (CDER), 2018); 4) Comparators: placebo or other types of aqueous INCSs. Exclusion criteria are patients with non-allergic rhinitis, rhinosinusitis, INCS in a formulation other than aqueous, studies with no abstract or available full-text, and duplicated published studies.

The primary outcomes were efficacy measured by TNSS changes from baseline and treatment acceptability (defined by the study dropout for any reason). The secondary outcomes were efficacy in improving ocular symptoms measured by the changes from baseline in total ocular symptom score (TOSS). All the outcomes were measured at week 2 for seasonal AR and week 4 for perennial AR after randomized assignments.

### 2.2 Search strategy

Electronic medical databases included PubMed/MEDLINE, Scopus, EMBASE, and the Cochrane Central Register of Controlled Trials (CENTRAL). A search strategy for each database is provided in Supplementary Table S1. A prespecified search strategy was used to search for relevant literature from its inception to the end of 31 March 2022. The authors also reviewed previous references from previously reported SR and/or meta-analyses on the same topic.

### 2.3 Study selection

Two review authors (KS and TK) independently screened titles and abstracts of all retrieved records from database searching to determine the eligible studies. These two authors retrieved and screened the full-text articles according to the prespecified inclusion and exclusion criteria. All studies chosen to be excluded were discussed, and the reasons for the exclusion were recorded. Any disagreement during this selection process was resolved by consulting a clinical expert in allergy (TT) and a clinical methodologist (PP).

### 2.4 Data extraction

Two reviewers (KS and CP) independently extracted the data: study and patient characteristics, including baseline symptom or severity score, definition or description of intervention and control treatments, factors with potential effect modification, and the outcomes of interest. All the extracted data was cross-checked and confirmed with the lead investigator (TT). To assess the plausibility of conducting NMA, we tabulated the study and clinical characteristics, including potential effect modifiers, to evaluate the transitivity assumptions to ensure systematic differences among all available treatment comparisons do not exist (Salanti, 2012; Rouse et al., 2017).

We extracted the exact mean change values and their standard deviations (SD) for each treatment arm from each study for continuous outcomes. However, for studies that did not directly report these values, we employed the methods suggested by the Cochrane Handbook for Systematic Reviews of Interventions (Higgins et al., 2019) and other relevant literature (Wan et al., 2014; Luo et al., 2018) to estimate the mean and SD values. For categorical outcomes, the total number of patients and events in each treatment arm were collected. If no events were identified, we imputed the zero value with 0.5 (Friedrich et al., 2007).

### 2.5 Risk of bias assessment

The internal validity of the included RCTs was evaluated using Risk-of-Bias 2 assessment tools (Sterne et al., 2019). Two authors (KS and TK) independently assessed the risk of bias. Any discrepancy during the assessment was resolved through discussion with PP and TT.

### 2.6 Grading quality of evidence

Two reviewers (KS and TK) independently graded the certainty evidence for each outcome using the Grading of Recommended Assessment, Development, and Evaluation (GRADE) approach (Puhan et al., 2014). All pairwise comparisons were rated based on their risk of bias, imprecision, inconsistency, and indirectness into four levels of evidence quality: high, moderate, low, and very low. Any disagreement was resolved through discussion with the clinical methodologist (PP).

### 2.7 Statistical analysis

Before conducting the meta-analysis, both clinical and methodological heterogeneity of each study were assessed to examine transitivity and trial homogeneity. Heterogeneity was assessed by Cochran’s Q test and Higgin’s I^2^ statistic, respectively (Higgins et al., 2003).

A pairwise meta-analysis was performed using a random-effects model by DerSimonian and Laird (DerSimonian and Laird, 1986). A random-effects NMA was performed using a frequentist approach to estimate the comparative efficacy among all available treatments (Lu and Ades, 2004; Rucker and Schwarzer, 2015). We planned to express the continuous outcomes using mean difference. As the TNSS and TOSS were continuous data with varying scales of measurements, standardized mean difference (SMD) was used. The interpretation of SMD was as follows: 0.2 for small, 0.5 for medium, and 0.8 for large effect (Cohen, 1988). In this study, we determined the cutoff for minimal clinically important difference at an SMD of 0.2 (Lemieux et al., 2007). For categorical outcomes, the odds ratio (OR) was used.

To ensure valid NMA estimates, we evaluated the consistency assumption using the global test, the loop-specific approach, and the node-splitting approach (Mills et al., 2013; Veroniki et al., 2013; Chaimani et al., 2017; Rouse et al., 2017). Treatment ranking for each outcome was based on the mean surface under the cumulative ranking (SUCRA). Rankograms and league tables are presented separately for each outcome. A hierarchical clustering analysis was conducted using two-dimensional plots to group treatments according to their underlying SUCRA on efficacy and acceptability (Chaimani et al., 2013). Publication bias was evaluated using a comparison-adjusted funnel plot of treatments (Chaimani and Salanti, 2012).

Sensitivity analyses were performed by excluding studies involving children, studies with a high risk of bias, studies published prior to 2000, and studies with small sample sizes (sample size less than the 10th percentile). We also conducted a leave-one-out sensitivity analysis in which we omitted one study from each round of analysis.

All statistical analyses were performed using Stata 17 (StataCorp, College Station, TX). Except for a *p*-value less than .10 for the heterogeneity test, a 2-tailed *p*-value less than .05 was considered statistical significance.

## 3 Results

### 3.1 Study selection and characteristics

**FIGURE 1:**
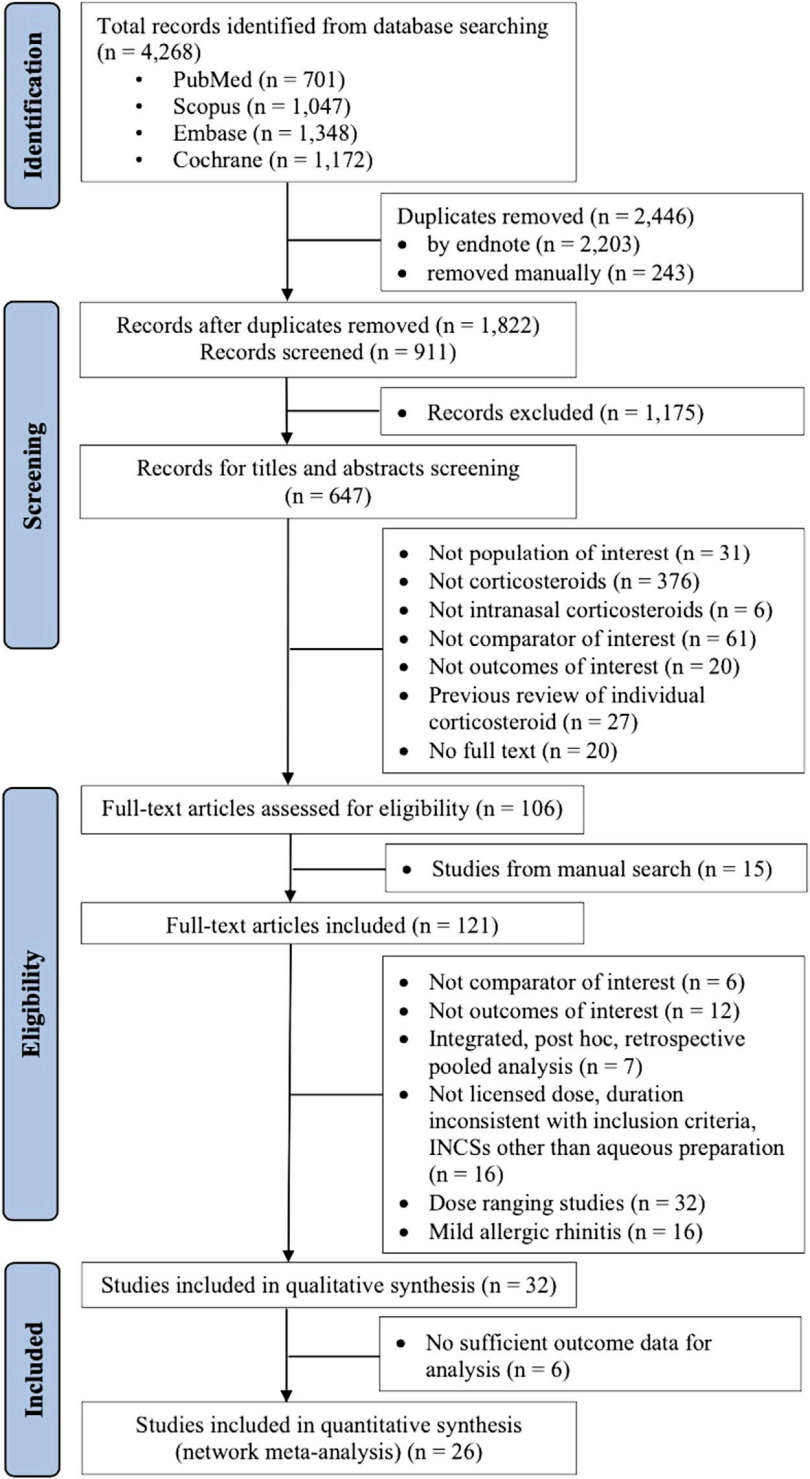
Preferred Reporting Items for Systematic Reviews and Meta-Analyses (PRISMA) flow diagram of included and excluded studies.

The systematic literature search details are provided in Figure 1. Screening titles and abstracts retrieved 121 full texts of potentially relevant studies. After screening those full texts, 89 studies were excluded, as shown in Supplementary Table S2. Thirty-two RCTs involving patients with moderate-to-severe AR were included for qualitative synthesis, as described in Table 1. Six studies had insufficient outcome data for quantitative synthesis (Ratner et al., 1992; van Bavel et al., 1994; Bronsky et al., 1996; Gross et al., 2002; Lumry et al., 2003; Meltzer et al., 2004). Therefore, only 26 studies, 13 with 5,134 SAR patients (Meltzer et al., 1998; Berger et al., 2003; Gawchik et al., 2003; Ratner et al., 2006; Fokkens et al., 2007; Kaiser et al., 2007; Andrews et al., 2009; Jacobs et al., 2009; Okubo et al., 2009; Prenner et al., 2010; Meltzer et al., 2011; Igarashi et al., 2012; Ratner et al., 2015) and 13 with 4,393 PAR patients (Kobayashi et al., 1995; Fokkens et al., 2002; Tai and Wang, 2003; Chervinsky et al., 2007; Meltzer et al., 2007; Rosenblut et al., 2007; Nathan et al., 2008; Vasar et al., 2008; Weinstein et al., 2009; Baena-Cagnani and Patel, 2010; Given et al., 2010; Meltzer et al., 2010; Karaulov et al., 2019), were included in NMA. Of 26 studies, 3 (Fokkens et al., 2002; Weinstein et al., 2009; Baena-Cagnani and Patel, 2010) were conducted in children and 23 in adults and adolescents. Three studies (Berger et al., 2003; Tai and Wang, 2003; Karaulov et al., 2019) compared active drug VS active drug, 1 (Okubo et al., 2009) compared 2 active drugs VS placebo, and 22 compared active drug VS placebo.

**TABLE 1:** Characteristics of included studies.

Studies	Site of study (no. of centers)	Type of RCTs	Duration of study (wk)	Study size (n)	Type of AR	Duration of AR (yr)	Intervention/comparator	Sample size (n) in each arm	Age (yr)^a^	Baseline TNSS (mean ± SD)	Baseline TOSS (mean ± SD)
Ratner et al. (2015)	United States (6)	double-blind	2	626	SAR	2	FP 200 µg OD	314	40.4 ± 14.55	NR	6.75 ± 1.364
							Placebo	312	40.5 ± 16.36	NR	6.98 ± 1.365
Igarashi et al. (2012)	Japan (1)	double-blind	4	11	SAR	NR	MF 200 µg OD	7	45.0 ± 7.9	4.86 ± 2.85	3.00 ± 1.85
							Placebo	4	44.0 ± 4.2	2.25 ± 2.27	2.75 ± 0.43
Meltzer et al. (2011)	United States (24)	double-blind	2	684	SAR	2	MF 200 µg OD	344	38.3 ± 13.8	9.5	NR
							Placebo	340	38.8 ± 13.9	9.58	NR
Prenner et al. (2010)	United States (25)	double-blind	2	429	SAR	2	MF 200 µg OD	220	34.5 ± 14.1	9.79 ± 1.44	6.95 ± 1.34
							Placebo	209	36.8 ± 14.5	9.82 ± 1.51	6.93 ± 1.43
Okubo et al. (2009)	Japan (7)	double-blind	2	446	SAR	2	FP 200 µg OD	148	32.1 ± 10.27	5.9 ± 1.43	NR
							Placebo	75	30.6 ± 10.2	NR	NR
							FF 110 µg OD	151	32.4 ± 10.98	5.8 ± 1.33	NR
							Placebo	72	32.5 ± 11.48	5.9 ± 1.28	NR
Jacobs et al. (2009)	United States (7)	double-blind	2	302	SAR	1	FF 110 µg OD	152	37.0 ± 13.9	9.8 ± 1.37	6.6 ± 1.34
							Placebo	150	38.1 ± 13.6	9.8 ± 1.37	6.5 ± 1.33
Andrews et al. (2009)	United States (10)	double-blind	2	936	SAR	1	FF 110 µg OD	312	37.8 ± 13.95	9.8 ± 1.59	7.0 ± 1.41
							Fexofenadine 180 mg OD	311	39.6 ± 14.63	10.0 ± 1.41	7.0 ± 1.41
							Placebo	313	37.8 ± 14.39	10.0 ± 1.42	7.1 ± 1.42
	United States (42)	double-blind	2	680	SAR	1	FF 110 µg OD	224	34.0 ± 13.55	10.0 ± 1.5	6.9 ± 1.5
							Fexofenadine 180 mg OD	227	34.3 ± 13.66	10.0 ± 1.66	7.1 ± 1.51
							Placebo	229	34.8 ± 12.71	9.9 ± 1.51	7.0 ± 1.36
Kaiser et al. (2007)	United States (17)	double-blind	2	299	SAR	1	FF 110 µg OD	148	35.4 ± 13.85	9.6 ± 1.56	6.6 ± 1.44
							Placebo	151	34.5 ± 14.09	9.9 ± 1.33	6.5 ± 1.47
Fokkens et al. (2007)	Europe (23)	double-blind	2	285	SAR	1	FF 110 µg OD	141	30.7 ± 11.7	8.3 ± 1.47	5.4 ± 1.23
							Placebo	144	29.4 ± 10.93	8.4 ± 1.35	5.3 ± 1.20
Ratner et al. (2006)	United States	double-blind	4	327	SAR	2	CIC 200 µg OD	164	39.6 ± 14	8.96 ± 1.96	NR
							Placebo	163	41 ± 15	8.83 ± 1.82	NR
Meltzer et al. (2004)	United States (1)	single-blind	3	39	SAR	2	TAA 220 µg OD	19	29.4 ± 17.9	NR	NR
							FP 200 µg OD	20	30.5 ± 14.4	NR	NR
Gawchik et al. (2003)	United States (11)	double-blind	2	245	SAR	1	MF 200 µg OD	122	34.7 (12–74)	11.6 ± 2.1	NR
							Placebo	123	34.2 (12–74)	10.8 ± 2.22	NR
Lumry et al. (2003)	United States (5)	single-blind	3	152	SAR	2	TAA 220 µg OD	75	36.2 (19–59)	6.8 ± 1.73	2.0 ± 0.1
							BDP 168 µg BID	77	37.5 (19–71)	7.1 ± 1.76	2.0 ± 0.1
Berger et al. (2003)	United States (9)	single-blind	3	295	SAR	2	TAA 220 µg OD	148	30.7 ± 14.2	8.06 ± 0.16	NR
							FP 200 µg OD	147	32.6 ± 12.9	7.64 ± 0.16	NR
Gross et al. (2002)	United States (8)	double-blind	3	352	SAR	2	TAA 220 µg OD	172	40.0 ± 12.2	8.95 ± 1.70	NR
							FP 200 µg OD	180	37.5 ± 12.4	9.01 ± 1.74	NR
Meltzer et al. (1998)	United States (1)	double-blind	2	128	SAR	2	MF 200 µg OD	85	12–65 (range)	7.32 ± 1.8	NR
							Placebo	43	12–65 (range)	7.68 ± 1.92	NR
Bronsky et al. (1996)	United States (10)	double-blind	4	348	SAR	1	FP 200 µg OD	117	30.4	271^b^	NR
							Terfenadine 60 mg BID	116	29.7	279^b^	NR
							Placebo	115	30.1	283^b^	NR
van Bavel et al. (1994)	United States (5)	double-blind	2	232	SAR	1	FP 200 µg OD	78	39.2	300^b^	NR
							Terfenadine 60 mg BID	77	39.8	300^b^	NR
							Placebo	77	40.1	300^b^	NR
Ratner et al. (1992)	United States (5)	double-blind	2	313	SAR	2	FP 200 µg OD	106	35 (18–65)	NR	NR
							BDP 168 µg BID	103	38.5 (18–66)	NR	NR
							Placebo	104	37.8 (19–72)	NR	NR
Karaulov et al. (2019)	Russia (12)	double-blind	4	260	PAR	1	TAA 220 µg OD	129	33.3 ± 8.5	10.3 ± 2.08	NR
							FP 200 µg OD	131	31.8 ± 8.47	10.1 ± 1.87	NR
Meltzer et al. (2010)	United States (1)	double-blind	4	30	PAR	2	MF 200 µg OD	20	34.6 (21–54)	18.68^c^	NR
							Placebo	10	34.4 (22–46)	17.57^c^	NR
Given et al. (2010)	United States, Canada and Europe (34)	double-blind	4	315	PAR	2	FF 110 µg OD	160	38.1 ± 14.2	9.1 ± 1.77	6.3 ± 1.77
							Placebo	155	39.3 ± 15.1	9.1 ± 1.62	6.6 ± 1.37
Baena-Cagnani and Patel (2010)	South America, Mexico, Canada and Europe (24)	double-blind	4	381	PAR	1	MF 100 µg OD	190	7.6 (3.0–11.0)	6.8	NR
							Placebo	191	7.4 (3.0–11.0)	6.8	NR
Weinstein et al. (2009)	United States (3)	double-blind	4	464	PAR	1	TAA 110 µg OD	231	3.6 ± 1.05	7.98 ± 1.96	NR
							Placebo	233	3.5 ± 1.04	7.86 ± 2.09	NR
Nathan et al. (2008)	United States, Canada (41)	double-blind	4	302	PAR	2	FF 110 µg OD	149	37.7 ± 14.93	8.6 ± 1.59	NR
							Placebo	153	35.8 ± 14.83	8.7 ± 1.73	NR
Vasar et al. (2008)	United States, Australia, New Zealand, Canada, and Europe (40)	double-blind	6	288	PAR	2	FF 110 µg OD	151	37.1 (12–76)	8.8 ± 1.87	4.6 ± 2.27
							Placebo	151	37.2 (12–77)	8.5 ± 1.43	4.2 ± 2.30
Meltzer et al. (2007)	United States (3)	double-blind	6	471	PAR	2	CIC 200 µg OD	238	35.66 ± 14.2	7.59	NR
							Placebo	233	35.37 ± 14.2	7.72	NR
Chervinsky et al. (2007)	United States (3)	double-blind	52	663	PAR	2	CIC 200 µg OD	441	37 (12–73)	6.4	NR
							Placebo	222	36 (12–68)	6.3	NR
Rosenblut et al. (2007)	13 countries worldwide	double-blind	52	806	PAR	2	FF 110 µg OD	605	32.7 ± 14.29	NR	NR
							Placebo	201	31.6 ± 14.65	NR	NR
Tai and Wang (2003)	Taiwan (1)	double-blind	8	24	PAR	0.5	FP 200 µg OD	14	43.2 ± 8.9	9.00 ± 3.64	NR
							BUD 200 µg BID	10	37.8 ± 11.7	9.80 ± 1.78	NR
Fokkens et al. (2002)	Netherlands, Hungary, Portugal (35)	double-blind	6	202	PAR	1	BUD 128 µg OD	100	10.5 (6–16)	4.62	NR
							Placebo	102	10.7 (6–16)	4.61	NR
Kobayashi et al. (1995)	United States (6)	double-blind	4	178	PAR	2	TAA 220 µg OD	88	32 (12–56)	6.5 ± 0.94	NR
							Placebo	90	30 (11–59)	6.4 + 0.95	NR

AR, allergic rhinitis; BDP, beclomethasone dipropionate; BID, twice daily; BUD, budesonide; CIC, ciclesonide; FF, fluticasone furoate; FP, fluticasone propionate; MF, mometasone furoate; NR, not reported; OD, once daily; PAR, perennial allergic rhinitis; SAR, seasonal allergic rhinitis; SD, standard deviation; TAA, triamcinolone acetonide; TNSS, total nasal symptom score; TOSS, total ocular symptom score; wk, week; yr, year.

^a^ The numbers indicate mean or mean ± SD or mean (range) unless stated otherwise.

^b^ Sum of visual analog scale ranging from 0 (none) to 100 (severe) on each symptoms, including nasal congestion, nasal itching, sneezing, and rhinorrhea.

^c^ Sum of nasal symptom scores, including nasal congestion, nasal itching, sneezing, and rhinorrhea, each rated from 0 (none) to 6 (severe).

**FIGURE 2:**
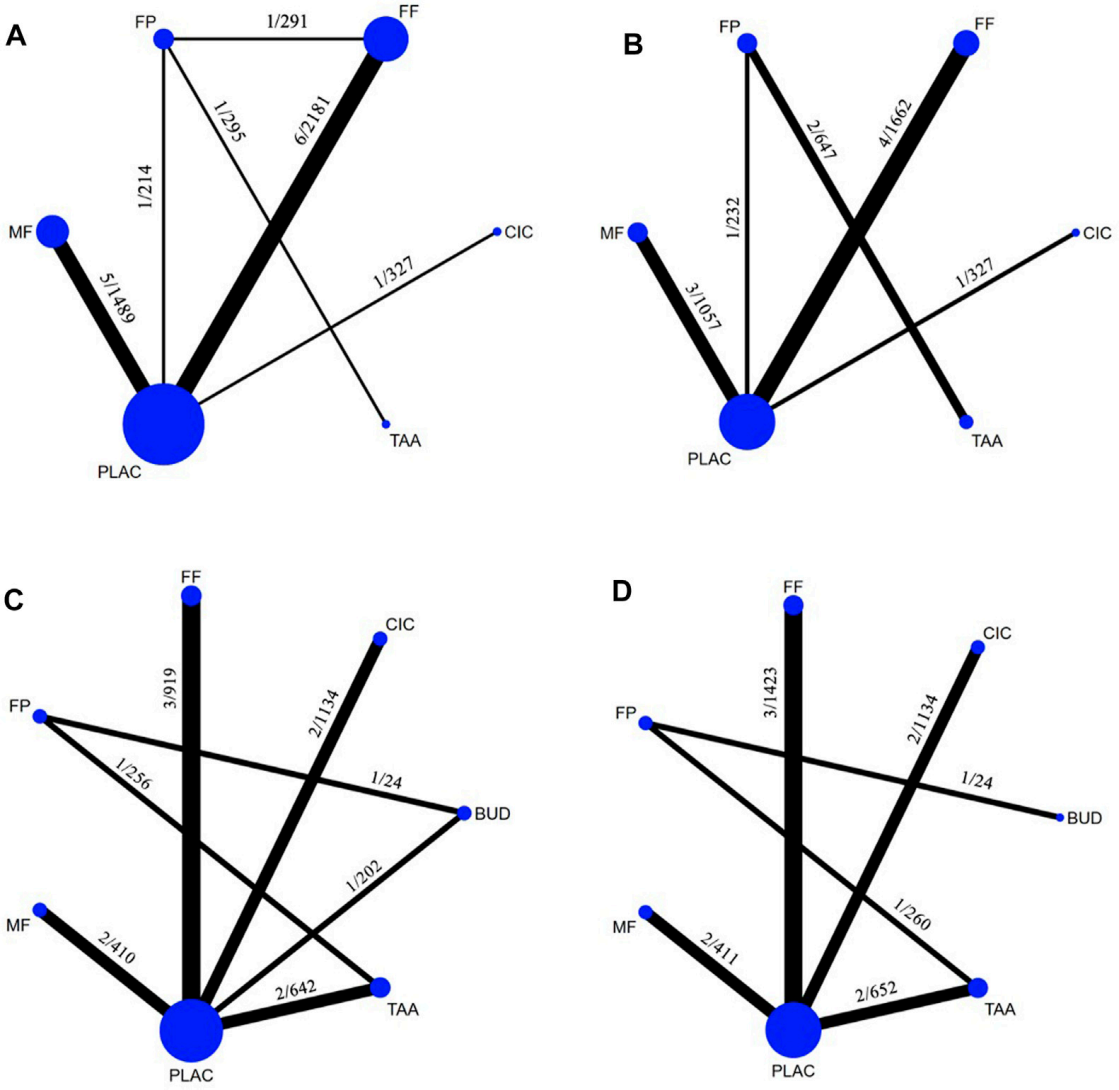
Networks of treatment comparisons according to the outcomes measured: **(A)**, Total nasal symptom score changes from baseline (12 studies, 15 treatment pairs, 4,508 patients) and **(B)**, Acceptability (10 studies, 11 treatment pairs, 3,925 patients) in seasonal allergic rhinitis; **(C)**, Total nasal symptom score changes from baseline (12 studies, 12 treatment pairs, 3,587 patients) and **(D)**, Acceptability (11 studies, 11 treatment pairs, 3,904 patients) in perennial allergic rhinitis. The thickness of the lines is proportional to the number of trials comparing each pair of treatments. The labels on each line represent the number of studies/the total number of patients involved in the comparison. The size of each circle is proportional to the number of randomly assigned participants. BUD, budesonide; CIC, ciclesonide; FF, fluticasone furoate; FP, fluticasone propionate; MF, mometasone furoate; TAA, triamcinolone acetonide.

The network diagrams of available comparison pairs for both efficacy and acceptability outcomes are illustrated in Figure 2. Details on the inclusion criteria, exclusion criteria, number, and reasons for withdrawals of each study are shown in Supplementary Table S3. Details on the outcome of interest, point of outcome measurements, and definitions of outcomes are shown in Supplementary Table S4.

### 3.2 Assessment of risk of bias

Regarding the quality of the studies, 26 studies were rated to have a low risk of bias, 4 studies had a high risk of bias, and 2 studies had some concerns (Supplementary Figure S1 and Supplementary Figure S2). Details of the risk-of-bias evaluation of each included study are shown in Supplementary Table S5.

### 3.3 Changes from baseline in total nasal symptom score in seasonal allergic rhinitis

**FIGURE 3:**
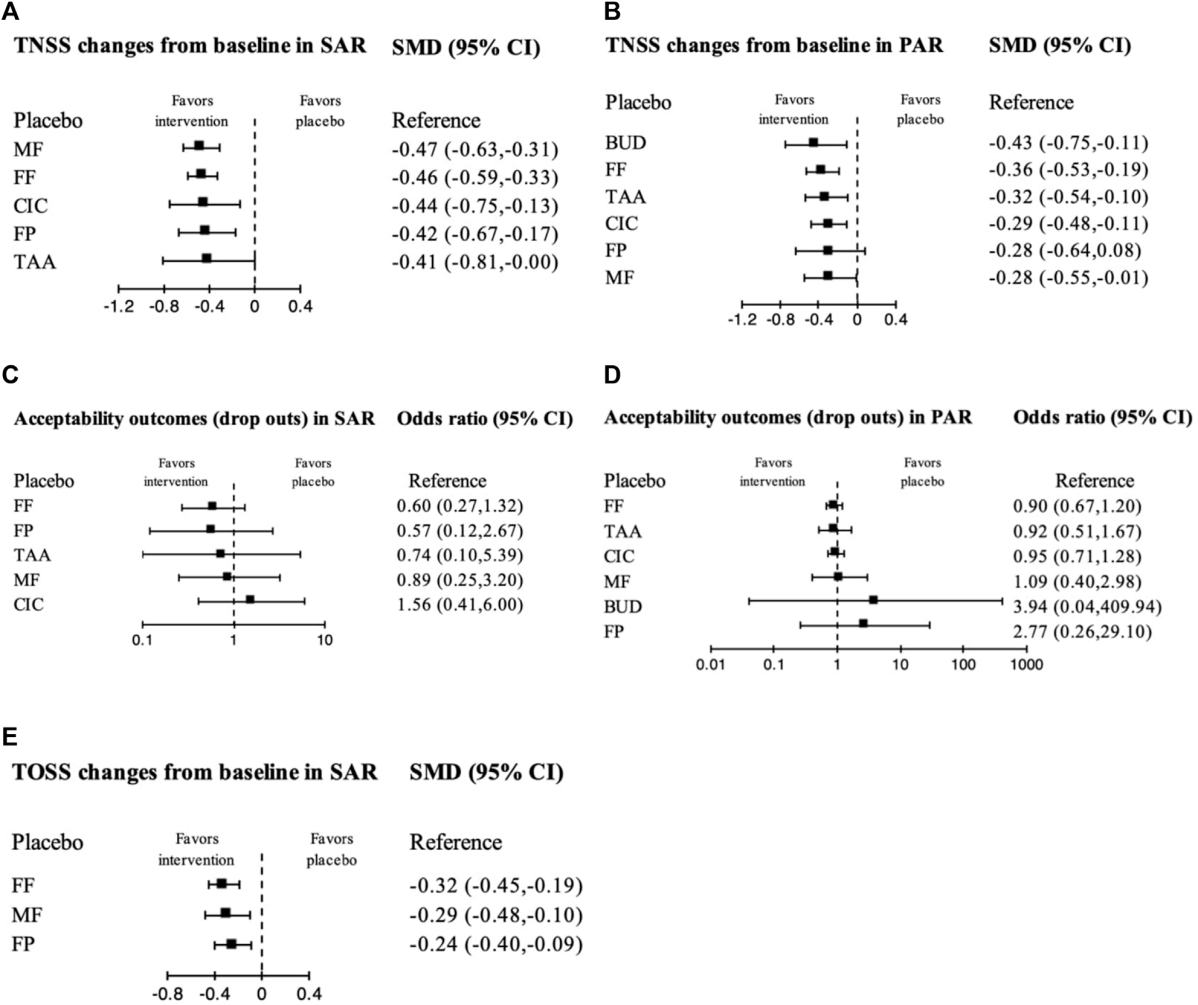
Forest plot showing network meta-analysis results of all treatment options compared with placebo using randomized controlled trials. The following figures show the effect sizes (standardized mean difference, SMD) of each treatment compared to the placebo and are presented separately according to the measured outcomes. **(A)**, Total nasal symptom score (TNSS) changes from baseline in seasonal allergic rhinitis (SAR); **(B)**, TNSS changes from baseline in perennial allergic rhinitis (PAR); **(C)**, Acceptability in SAR; **(D)**, Acceptability in PAR; **(E)**, Total ocular symptom score (TOSS) changes from baseline in SAR. CI, Confidence interval; BUD, budesonide; CIC, ciclesonide; FF, fluticasone furoate; FP, fluticasone propionate; MF, mometasone furoate; TAA, triamcinolone acetonide.

The TNSS outcome was available in 12 SAR studies involving 5 INCSs (Figure 2A). MF, FF, CIC, FP, and TAA, significantly had superior efficacy to placebo with small treatment effects [SMD −0.47 (95% CI: −0.63 to −0.31), −0.46 (95% CI: −0.59 to −0.33), −0.44 (95% CI: −0.75 to −0.13), −0.42 (95% CI: −0.67 to −0.17), and −0.41 (95% CI: −0.81 to −0.00), respectively] (Table 2; Figure 3A). Based on SUCRA, MF was ranked the highest efficacy, followed by FF, CIC, FP, and TAA, respectively (Supplementary Figure S3A).

**TABLE 2:** League table of efficacy measured by standardized mean difference for total nasal symptom score changes from baseline and acceptability outcome in patients with seasonal allergic rhinitis.

MF	0.67 (0.15,2.97)	0.57 (0.09,3.65)	0.64 (0.09,4.74)	0.83 (0.08,8.82)	0.89 (0.25,3.20)
−0.01 (−0.21,0.19)	**FF**	0.38 (0.08,1.83)	1.06 (0.19,6.01)	0.81 (0.10,6.92)	0.60 (0.27,1.32)
−0.03 (−0.38,0.32)	−0.02 (−0.36,0.32)	**CIC**	0.36 (0.05,2.82)	0.47 (0.04,5.21)	1.56 (0.41,6.00)
−0.05 (−0.35,0.25)	−0.04 (−0.29,0.21)	−0.02 (−0.42,0.38)	**FP**	0.77 (0.22,2.68)	0.57 (0.12,2.67)
−0.06 (−0.50,0.38)	−0.05 (−0.45,0.35)	−0.03 (−0.54,0.48)	−0.01 (−0.33,0.31)	**TAA**	0.74 (0.10,5.39)
**−0.47 (-0.63,-0.31)**	**−0.46 (-0.59,-0.33)**	**−0.44 (-0.75,-0.13)**	**−0.42 (-0.67,-0.17)**	**−0.41 (-0.81,-0.00)**	**PLAC**

MF, mometasone furoate; FF, fluticasone furoate; CIC, ciclesonide; FP, fluticasone propionate; TAA, triamcinolone acetonide; PLAC, placebo.

Bold values indicate statistical significance.

The numbers highlighted with yellow color in the lower-left portion represent standardized mean difference (SMD) for total nasal symptom score (TNSS) changes from baseline in seasonal allergic rhinitis and the numbers highlighted with blue color in the upper-right portion represent acceptability outcome (study discontinuation or dropout) in patients with seasonal allergic rhinitis. Treatments are arranged in order of the mean ranking from network meta-analysis of TNSS, from the best (left) to the worst (right).

### 3.4 Changes from baseline in total nasal symptom score in perennial allergic rhinitis

The TNSS outcome was available in 12 PAR studies involving 6 INCSs (Figure 2C). BUD, FF, TAA, CIC, and MF, significantly had superior efficacy to placebo with small treatment effects [SMD −0.43 (95% CI: −0.75 to −0.11), −0.36 (95% CI: −0.53 to −0.19), −0.32 (95% CI: −0.54 to −0.10), −0.29 (95% CI: −0.48 to −0.11), and −0.28 (95% CI: −0.55 to −0.01), respectively], while FP had no significant treatment effect compared with placebo [SMD-0.28 (95% CI: −0.64 to 0.08)] (Table 3; Figure 3B). Based on SUCRA, BUD was ranked the highest efficacy, followed by FF, TAA, CIC, and MF (Supplementary Figure S3B).

**TABLE 3:** League table of efficacy measured by standardized mean difference for total nasal symptom score changes from baseline and acceptability outcome in patients with perennial allergic rhinitis.

BUD	0.23 (0.00,24.05)	0.23 (0.00,23.53)	1.42 (0.03,78.11)	0.24 (0.00,25.49)	0.28 (0.00,32.22)	3.94 (0.04,409.94)
−0.07 (−0.43,0.30)	**FF**	0.98 (0.51,1.88)	0.33 (0.03,3.48)	0.94 (0.62,1.43)	0.82 (0.29,2.34)	0.90 (0.67,1.20)
−0.11 (−0.49,0.27)	−0.04 (−0.32,0.23)	**TAA**	0.33 (0.03,3.25)	0.97 (0.50,1.87)	0.23 (0.00,23.53)	0.92 (0.51,1.67)
−0.15 (−0.60,0.30)	−0.08 (−0.48,0.32)	−0.04 (−0.33,0.26)	**FP**	0.34 (0.03,3.69)	0.40 (0.03,5.09)	2.77 (0.26,29.10)
−0.13 (−0.50,0.24)	−0.07 (−0.32,0.19)	−0.02 (−0.31,0.26)	0.01 (−0.39,0.42)	**CIC**	0.87 (0.31,2.48)	0.95 (0.71,1.28)
−0.15 (−0.57,0.28)	−0.08 (−0.40,0.24)	−0.03 (−0.37,0.30)	0.00 (−0.44,0.44)	−0.01 (−0.34,0.32)	**MF**	1.09 (0.40,2.98)
**−0.43 (-0.75,-0.11)**	**−0.36 (-0.53,-0.19)**	**−0.32 (-0.54,-0.10)**	−0.28 (−0.64,0.08)	**−0.29 (-0.48,-0.11)**	**−0.28 (-0.55,-0.01)**	**PLAC**

BUD, budesonide; FF, fluticasone furoate; TAA, triamcinolone acetonide; FP, fluticasone propionate; CIC, ciclesonide; MF, mometasone furoate; PLAC, placebo.

Bold values indicate statistical significance.

The numbers highlighted with yellow color in the lower-left portion represent standardized mean difference (SMD) for total nasal symptom score (TNSS) changes from baseline in perennial allergic rhinitis and the numbers highlighted with blue color in the upper-right portion represent acceptability outcome (study discontinuation or dropout) in patients with perennial allergic rhinitis. Treatments are arranged in order of the mean ranking from network meta-analysis of TNSS, from the best (left) to the worst (right).

### 3.5 Changes from baseline in total ocular symptom score

Six SAR studies (Fokkens et al., 2007; Kaiser et al., 2007; Jacobs et al., 2009; Prenner et al., 2010; Igarashi et al., 2012; Ratner et al., 2015) involving 3 INCSs provided TOSS outcomes for NMA (Supplementary Figure S4A). FF, MF, and FP significantly improved TOSS with small treatment effects compared with the placebo [SMD -0.32 (95% CI: −0.45 to −0.19), −0.29 (95% CI: −0.48 to −0.10), and −0.24 (95% CI: −0.40 to −0.09), respectively] (Supplementary Table S6 and Figure 3E). Based on SUCRA, FF was ranked the highest efficacy, followed by MF and FP (Figure 3E and Supplementary Figure S4B).

### 3.6 Acceptability of treatments

Networks for acceptability outcomes in SAR and PAR are illustrated in Figures 2B, D. An assessment of 10 SAR studies (Bronsky et al., 1996; Meltzer et al., 1998; Gross et al., 2002; Berger et al., 2003; Gawchik et al., 2003; Ratner et al., 2006; Fokkens et al., 2007; Kaiser et al., 2007; Andrews et al., 2009; Meltzer et al., 2011) and 11 PAR studies (Kobayashi et al., 1995; Tai and Wang, 2003; Chervinsky et al., 2007; Meltzer et al., 2007; Rosenblut et al., 2007; Vasar et al., 2008; Weinstein et al., 2009; Baena-Cagnani and Patel, 2010; Given et al., 2010; Meltzer et al., 2010; Karaulov et al., 2019) for the acceptability of treatments found that all INCSs were comparable to placebo in acceptability outcome without statistical significance (Table 2; Table 3; Figures 3C, D). Based on SUCRA, The ranking in treatment acceptability in SAR and PAR is shown in Supplementary Figure S3C and Supplementary Figure S3D, respectively.

### 3.7 Hierarchical cluster analysis

**FIGURE 4:**
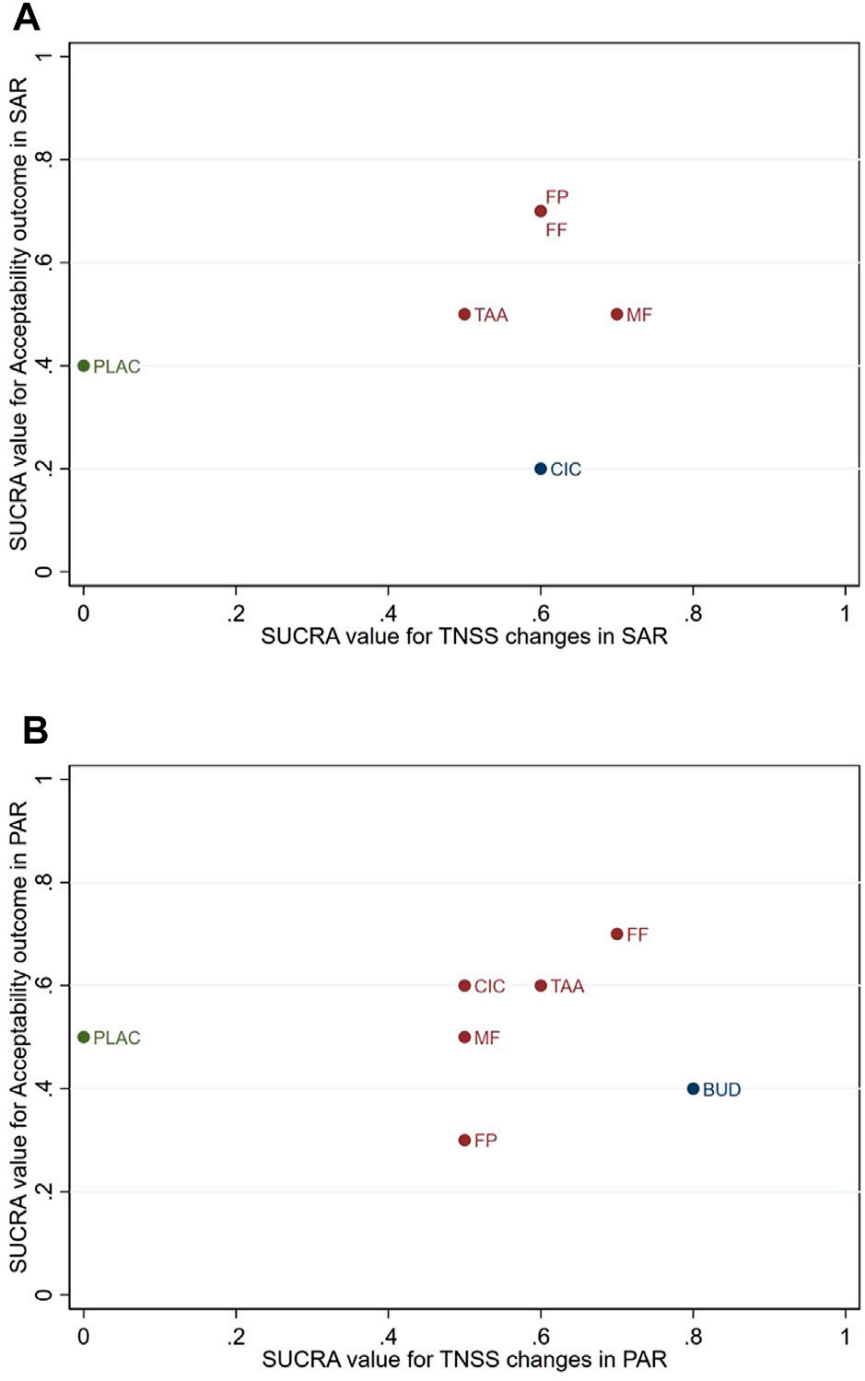
Cluster ranking based on the surface under the cumulative ranking (SUCRA) for changes in the total nasal symptom score (TNSS) from baseline and acceptability outcome via any cause of dropout in patients with **(A)**, seasonal allergic rhinitis (SAR), and **(B)**, perennial allergic rhinitis (PAR). BUD, budesonide; CIC, ciclesonide; FF, fluticasone furoate; FP, fluticasone propionate; MF, mometasone furoate; PLAC, placebo; TAA, triamcinolone acetonide.

The SUCRA values for treatment efficacy in improving the TNSS, TOSS, and acceptability by the patient were used in the hierarchical cluster analysis. Regarding SAR (Figure 4A), FF, FP, MF, and TAA were classified into groups with high efficacy and acceptability, with FF and FP being equivalent in both outcomes. CIC had high efficacy but the lowest acceptability. Regarding PAR (Figure 4B), CIC, FF, MF, and TAA were classified into groups with high efficacy and acceptability. FP also had high efficacy but low acceptability. BUD had the highest efficacy in PAR but had low acceptability, with FP being the lowest SUCRA for acceptability. Regarding TOSS (Supplementary Figure S4B), FF, FP, and MF had high efficacy and high acceptability, with FF being the highest in both outcomes.

### 3.8 Subgroup analyses and sensitivity analyses

The results of the sensitivity analyses are shown in Supplementary Table S7. Concerning the TNSS changes in SAR, MF was ranked with the highest efficacy, followed by FF in the primary analysis of 12 studies. By excluding one study (*n* = 11) with a small sample size and a significant risk of bias (Igarashi et al., 2012), both MF and FF became the first rank. In contrast, by excluding one study with SD imputation (Meltzer et al., 2011), FF became the highest efficacy, followed by MF. The results in the leave-one-out sensitivity analysis also went in the same direction and magnitude of effect estimates.

Regarding the TNSS changes in PAR, BUD was ranked with the highest efficacy, followed by FF and TAA in the primary analysis of 12 studies. By excluding two with a significant risk of bias (Chervinsky et al., 2007; Vasar et al., 2008), and two studies with a small sample size (Tai and Wang, 2003; Meltzer et al., 2010), BUD remained in the first rank. In contrast, by excluding three studies in children (Fokkens et al., 2002; Weinstein et al., 2009; Baena-Cagnani and Patel, 2010), MF became the first rank, followed by TAA. However, the leave-one-out sensitivity analysis results were consistent with the primary analysis.

Concerning the TOSS changes in SAR, FF was ranked with the highest efficacy, followed by MF and FP in the primary analysis of 6 studies. The results remained consistent with the primary analysis by excluding one study with a small sample size (Igarashi et al., 2012).

### 3.9 Heterogeneity, inconsistency, transitivity, publication bias, and strength of evidence

From the results of the pairwise meta-analysis, there was some evidence of moderate-to-high statistical heterogeneity among included studies, especially in the treatment outcomes of TNSS and TOSS changes (Supplementary Table S8 and Supplementary Table S9). Evidence of inconsistency between direct and indirect evidence was not identified from the global design-by-treatment interaction model and 2 loops of treatment efficacy (Supplementary Table S10A, B). No significant evidence of asymmetry was found in the analysis of comparison-adjusted funnel plots (Supplementary Figure S5). We graded the strength of evidence for the synthesized NMA estimates by considering all relevant domains and assumptions. Most placebo-controlled studies were rated as having a moderate quality of evidence, while active-controlled studies were rated as having very low to low quality of evidence for the TNSS outcomes in SAR and PAR. The grading summary is provided separately for placebo-controlled and active-controlled comparisons in Supplementary Table S11 and Supplementary Table S12.

## 4 Discussion

### 4.1 Summary of main findings

The present NMA included 26 studies, 13 with 5,134 SAR patients and 13 with 4,393 PAR patients. Most studies were placebo-controlled trials with moderate quality of evidence. In SAR, MF was ranked the highest efficacy in improving TNSS, followed by FF, CIC, FP, and TAA. In PAR, BUD was ranked the highest efficacy in improving TNSS, followed by FF, TAA, CIC, and MF. FF was ranked the highest efficacy in improving TOSS in SAR, followed by MF and FP. The acceptability of all included INCSs was not inferior to the placebo.

Some INCSs were missing in this NMA because no BDP studies in PAR, BUD studies in SAR, and flunisolide studies in both SAR and PAR met our inclusion criteria. Two BDP studies in SAR were included in the qualitative analysis, but there was no sufficient data for NMA (Ratner et al., 1992; Lumry et al., 2003). However, those 2 studies showed that BDP was as effective as FP and TAA. Our methodology requires a 2-week and 4-week study duration for SAR and PAR to be consistent with the USFDA guidance for conducting INCS trials (United States Department of Health and Human Services Food and Drug Administration Center for Drug Evaluation and Research (CDER), 2018). Including patients with moderate-to-severe AR corresponds to an indication of INCS recommended by the standard guideline (United States Department of Health and Human Services Food and Drug Administration Center for Drug Evaluation and Research (CDER), 2018; Bousquet et al., 2020; Dykewicz et al., 2020). Excluding patients with mild AR would help distinguish the efficacy among INCSs since any INCSs may be effective in AR with mild severity, irrespective of their pharmacological profiles. Although the visual analog scale has recently become popular for grading AR severity, it was clearly shown to correlate well with TNSS used in our study (Klimek et al., 2017). These robust prespecified criteria allowed us to assure the transitivity of the network, minimize heterogeneity and enhance the applicability of the results to clinical practice.

Concerning the comparative efficacy, all INCSs were superior to placebo in either SAR or PAR or both. However, those INCSs showed an improvement in TNSS and TOSS with a small treatment effect compared with placebo, reflecting their similar efficacy. In addition, 8 out of 32 studies comparing FF VS FP (Okubo et al., 2009), FP VS TAA (Gross et al., 2002; Berger et al., 2003; Meltzer et al., 2004; Karaulov et al., 2019), BDP VS TAA (Lumry et al., 2003), BDP VS FP (Ratner et al., 1992), and BUD VS FP (Tai and Wang, 2003) demonstrated the equivalent efficacy between each paired comparison. These findings suggest that the licensed dose of any INCSs is sufficient to control allergic inflammation, irrespective of their different pharmacological profiles. Nevertheless, in our NMA, FP failed to show superior efficacy to placebo in PAR because no placebo-controlled FP studies agreed with our prespecified inclusion criteria. The indirect comparison results came from two active-controlled studies comparing FP VS TAA (*n* = 260) (Karaulov et al., 2019) and FP VS BUD (*n* = 24) (Tai and Wang, 2003) in PAR. Although FP was as efficacious as its comparators in those two studies, a limited number of studies with insufficient sample size may, at least in part, account for its insignificant efficacy compared with a placebo.

Apart from efficacy, safety is also crucial for choosing an INCS. Using standard-dose INCSs is usually safe for the adult population (Donaldson et al., 2020). In contrast, some INCSs, including BDP, TAA, and FF, had evidence of long-term effects on growth retardation in children (Skoner et al., 2000; Lee et al., 2014; Skoner et al., 2015). Another factor concerning INCS selection is patient preference and satisfaction, which could be affected by odor, taste, types of delivery devices, and cost (Sher and Ross, 2014). Patient-physician interaction to understand the differences among INCS products is essential to accomplish the treatment of AR.

### 4.2 Strengths and limitations

The primary strength of our study was the use of strict inclusion criteria in scoping the domain of patients and the timing of outcome measurement to ensure that all included RCTs were homogeneous enough to address our specific clinical questions. However, our study carries some limitations. First, a limited number of RCTs were included for each specific outcome. Some studies could not be included due to the stringent inclusion criteria. Thus, statistical significance among head-to-head comparisons could not be demonstrated, and the ranking sequence might be alternated by chance. We suggested that the interpretation of results should not be weighted entirely on the statistical significance and SUCRA but on the magnitude of the effect estimates and the certainty of evidence, which takes into account multiple aspects affecting the credibility of the results (e.g., the heterogeneity between each pairwise comparison, the imprecision of the estimates, and the inconsistency between the direct and indirect evidence) (Brignardello-Petersen et al., 2018). Second, there was an insufficient number of studies in each comparative pair to evaluate the source of heterogeneity using meta-regression or subgroup analysis. Third, new evidence after concluding the database search in March 2022 might have been reported so far.

## Conclusion

According to our NMA, MF was ranked the highest efficacy in improving TNSS in SAR, followed by FF, CIC, FP, and TAA. BUD was ranked the highest efficacy in improving TNSS in PAR, followed by FF, TAA, CIC, and MF. FF was ranked the highest efficacy in improving TOSS in SAR, followed by MF and FP. In addition to efficacy, other factors, including safety, cost, patient preference, and education, should be considered to improve long-term adherence and achieve AR control.
