# Supplementary material for: Comparative efficacy and acceptability of licensed dose intranasal corticosteroids for moderate-to-severe allergic rhinitis: a systematic review and network meta-analysis
Source: Front Pharmacol. 2023 May 23;14:1184552. doi: 10.3389/fphar.2023.1184552 (PMC10242043; doi:10.3389/fphar.2023.1184552)
Supplement: Supplementary file 1 [file DataSheet1.pdf]

Supplementary Material

Comparative efficacy and acceptability of licensed dose intranasal corticosteroids for moderate-to-severe allergic rhinitis: A systematic review and network meta-analysis

Kay Khine Soe, MD, Thanachit Krikeerati, MD, Chatkamol Pheerapanyawaranun, MSc, Suvimol Niyomnaitham, MD, PhD, Phichayut Phinyo, MD, PhD, Torpong Thongngarm, MD*

*** Correspondence:** Torpong Thongngarm, MD: torallergy@gmail.com

Supplementary Table S1. Searching strategies from A) PubMed, B) Scopus, C) Embase, and D) Cochrane Trial

Search in MEDLINE via PubMed

Domain		Search terms	items found
P	#1	allergic rhinitis [MeSH Terms]	22,746
	#2	"allergic rhinitis" [Text word]	30,172
	#3	#1 OR #2	30,728
I	#4	corticosteroid [MeSH Terms]	297,110
	#5	corticosteroid* [Text word]	114,687
	#6	ciclesonide [Text word]	444
	#7	fluticasone furoate [Text word]	463
	#8	fluticasone propionate [Text word]	2,491
	#9	mometasone [Text word]	1,269
	#10	beclomethasone [Text word]	3,928
	#11	budesonide [Text word]	6,814
	#12	flunisolide [Text word]	381
	#13	triamcinolone [Text word]	12,456
	#14	#4 OR #5 OR #6 OR #7 OR #8 OR #9 OR #10 OR #11 OR #12 OR #13	376,661
C	#15	placebo [MeSH Terms]	39,094
	#16	placebo [Text word]	233,638
	#17	#15 OR #16	250,520
I + C	#18	#14 OR #17	615,110
O	#19	total nasal symptom score [Text word]	394
	#20	tnss [Text word]	333
	#21	total ocular symptom score [Text word]	56
	#22	toss [Text word]	524
	#23	combined symptom-medication scores [Text word]	12
	#24	CSMS [Text word]	264
	#25	Rhinoconjunctivitis Quality of Life Questionnaire score [Text word]	23
	#26	RQLQ score [Text word]	67
	#27	visual analog scale [Text word]	33,486
	#28	VAS [Text word]	58,484
	#29	individual nasal symptom score [Text word]	8
	#30	nasal peak inspiratory flow [Text word]	83
	#31	acoustic rhinometry [Text word]	1,070
	#32	acoustic rhinomanometry [Text word]	16

Domain		Search terms	items found
	#33	quality-of-life score [Text word]	2,232
	#34	QoL score [Text word]	2,106
	#35	adverse events [Text word]	175,979
	#36	#19 OR #20 OR #21 OR #22 OR #23 OR #24 OR #25 OR #26 OR #27 OR #28 OR #29 OR #30 OR #31 OR #32 OR #33 OR #34 OR #35	254,153
PICO	#37	#3 AND #18 AND #36	880
RCT	#38	randomized [Text word]	947,266
	#39	randomised [Text word]	120,000
	#40	"randomized trial" [Text word]	49,992
	#41	"randomised trial" [Text word]	10,356
	#42	"randomized controlled trial" [Text word]	597,186
	#43	"randomised controlled trial" [Text word]	28,035
	#44	#38 OR #39 OR #40 OR #41 OR #42 OR #43	988,368
PubMed	#45	#37 AND #44	701

Search in Scopus

Domain		Search terms	items found
P	#1	TITLE-ABS-KEY (“allergic rhinitis”)	35,884
	#2	TITLE-ABS-KEY (“hay fever”)	13,027
	#3	#1 OR #2	44,872
I	#4	TITLE-ABS-KEY (corticosteroids)	327,451
	#5	TITLE-ABS-KEY (ciclesonide)	1,694
	#6	TITLE-ABS-KEY (“fluticasone furoate”)	1,224
	#7	TITLE-ABS-KEY (“fluticasone propionate”)	10,194
	#8	TITLE-ABS-KEY (mometasone)	5,266
	#9	TITLE-ABS-KEY (beclomethasone)	4,697
	#10	TITLE-ABS-KEY (budesonide)	22,467
	#11	TITLE-ABS-KEY (flunisolide)	2,473
	#12	TITLE-ABS-KEY (triamcinolone)	32,771
	#13	#4 OR #5 OR #6 OR #7 OR #8 OR #9 OR #10 OR #11 OR #12	367,341
C	#14	TITLE-ABS-KEY (placebo)	418,684
I + C	#15	#13 OR #14	762,849
O	#16	TITLE-ABS-KEY (“total nasal symptom score”)	603
	#17	TITLE-ABS-KEY (tnss)	396
	#18	TITLE-ABS-KEY (“total ocular symptom score”)	82
	#19	TITLE-ABS-KEY (toss)	2,037
	#20	TITLE-ABS-KEY (“combined symptom-medication scores”)	40
	#21	TITLE-ABS-KEY (csms)	635
	#22	TITLE-ABS-KEY (“rhinoconjunctivitis Quality of Life Questionnaire score”)	48
	#23	TITLE-ABS-KEY (“rqlq score”)	120
	#24	TITLE-ABS-KEY (“rhinitis control assessment test”)	39
	#25	TITLE-ABS-KEY (rcat)	214
	#26	TITLE-ABS-KEY (“visual analog scale”)	105,122
	#27	TITLE-ABS-KEY (vas)	126,198
	#28	TITLE-ABS-KEY (“individual nasal symptom score”)	35
	#29	TITLE-ABS-KEY (“nasal peak inspiratory flow”)	89
	#30	TITLE-ABS-KEY (“acoustic rhinometry”)	1,301
	#31	TITLE-ABS-KEY (“acoustic rhinomanometry”)	17
	#32	TITLE-ABS-KEY (“quality-of-life score”)	7,709
	#33	TITLE-ABS-KEY (“QoL score”)	6,562
	#34	TITLE-ABS-KEY (“adverse events”)	340,448

Domain	Search terms		items found
	#35	#16 OR #17 OR #18 OR #19 OR #20 OR #21 OR #22 OR #23 OR #24 OR #25 OR #26 OR #27 OR #28 OR #29 OR #30 OR #31 OR #32 OR #33 OR #34	536,351
PICO	#36	#3 AND #15 AND #27	1,498
RCT	#37	TITLE-ABS-KEY (randomized)	1,212,154
	#38	TITLE-ABS-KEY (“randomized trial”)	110,214
	#39	TITLE-ABS-KEY (“randomized controlled trial”)	886,236
	#40	#37 OR #38 OR #39	1,212,154
SCOPUS	#41	#36 AND #40	1,047

Search in Embase

Domain	Search terms		items found
P	#1	"allergic rhinitis"	56,857
	#2	"hay fever"	4,884
	#3	#1 OR #2	57,481
I	#4	corticosteroid	337,122
	#5	corticosteroid*	368,331
	#6	ciclesonide	1,894
	#7	"fluticasone furoate"	1,671
	#8	"fluticasone propionate"	11,577
	#9	mometasone	5,904
	#10	beclomethasone	9,906
	#11	budesonide	25,595
	#12	flunisolide	2,472
	#13	triamcinolone	34,179
	#14	#4 OR #5 OR #6 OR #7 OR #8 OR #9 OR #10 OR #11 OR #12 OR #13	413,969
C	#15	placebo	499,263
I + C	#16	#14 OR #15	886,426
O	#17	"total nasal symptom score"	763
	#18	tnss	667
	#19	"total ocular symptom score"	133
	#20	toss	1,144
	#21	"combined symptom-medication scores"	27
	#22	csms	407
	#23	"rhinoconjunctivitis Quality of Life Questionnaire score"	27
	#24	"rqlq score"	122
	#25	"rhinitis control assessment test"	62
	#26	rcat	142
	#27	"visual analog scale"	113,544
	#28	vas	102,554
	#29	"individual nasal symptom score"	9
	#30	"nasal peak inspiratory flow"	112
	#31	"acoustic rhinometry"	1,394
	#32	"acoustic rhinomanometry"	22
	#33	"quality of life score"	3,860
	#34	"QoL score"	4,292
	#35	"adverse events"	309,763

Domain	Search terms		items found
	#36	#17 OR #18 OR #19 OR #20 OR #21 OR #22 OR #23 OR #24 OR #25 OR #26 OR #27 OR #28 OR #29 OR #30 OR #31 OR #32 OR #33 OR #34 OR #35	476,897
PICO	#37	#3 AND #16 AND #36	1,929
RCT	#38	randomized	1,273,610
	#39	randomised	174,106
	#40	“randomized trial”	70,082
	#41	“randomised trial”	14,405
	#42	“randomized controlled trial”	938,032
	#43	“randomised controlled trial”	37,623
	#44	#38 OR #39 OR #40 OR #41 OR #42 OR #43	1,334,935
Embase	#45	#37 AND #44	1,348

Search in Cochrane Library

Domain	Search terms		items found
P	#1	"allergic rhinitis"	6,925
	#2	"hay fever"	700
	#3	#1 OR #2	7,325
I	#4	corticosteroid	15,202
	#5	corticosteroid*	25,138
	#6	ciclesonide	580
	#7	"fluticasone furoate"	831
	#8	"fluticasone propionate"	3,511
	#9	mometasone	1,429
	#10	beclomethasone	2,479
	#11	budesonide	5,235
	#12	flunisolide	301
	#13	triamcinolone	3,609
	#14	#4 OR #5 OR #6 OR #7 OR #8 OR #9 OR #10 OR #11 OR #12 OR #13	35,693
C	#15	placebo	349,655
I + C	#16	#14 OR #15	372,613
O	#17	"total nasal symptom score"	484
	#18	tnss	473
	#19	"total ocular symptom score"	97
	#20	toss	772
	#21	"combined symptom-medication scores"	0
	#22	csms	96
	#23	"rhinoconjunctivitis Quality of Life Questionnaire score"	16
	#24	"rqlq score"	84
	#25	"rhinitis control assessment test"	21
	#26	rcat	27
	#27	"visual analog scale"	52,071
	#28	vas	42,036
	#29	"individual nasal symptom score"	4
	#30	"nasal peak inspiratory flow"	55
	#31	"acoustic rhinometry"	253
	#32	"acoustic rhinomanometry"	8
	#33	"quality of life score"	2,205
	#34	"QoL score"	1,266
	#35	"adverse events"	117,659

Domain	Search terms		items found
	#36	#17 OR #18 OR #19 OR #20 OR #21 OR #22 OR #23 OR #24 OR #25 OR #26 OR #27 OR #28 OR #29 OR #30 OR #31 OR #32 OR #33 OR #34 OR #35	179,393
PICO	#37	#3 AND #16 AND #36	1,299
RCT	#38	randomized	1,252,129
	#39	“randomized trial”	91,562
	#40	“randomized controlled trial”	953,468
	#41	#38 OR #39 OR #40	1,252,129
Cochrane Library	#42	#37 AND #41	1,172

**Sources of database**	**items found**
MEDLINE via PubMed	701
Scopus via Scopus	1,047
Embase	1,348
Cochrane Controlled Trials Register (CCTR)	1,172
Total	4,268

Supplementary Table S2. List of studies excluded at full-text screening stage, with brief reasons

No.	Studies	References	Reasons for exclusion
1	Intranasal fluticasone furoate in pediatric allergic rhinitis: randomized controlled study	Zhang Y, Wei P, Chen B, Li X, Luo X, Chen X, et al. Intranasal fluticasone furoate in pediatric allergic rhinitis: randomized controlled study. Pediatr Res. 2021;89(7):1832-9.	dose ranging study
2	Fast effectiveness of a solubilized low-dose budesonide nasal spray in allergic rhinitis	Zieglmayer P, Schmutz R, Lemell P, Unger-Manhart N, Nakowitsch S, Goessl A, et al. Fast effectiveness of a solubilized low-dose budesonide nasal spray in allergic rhinitis. Clin Exp Allergy. 2020;50(9):1065-77.	not outcome of interest
3	Isotonic nasal spray versus fluticasone nasal spray in treatment of allergic rhinitis	Gaddipatti R, Rao S, Rajiv K, Jain M. Isotonic nasal spray versus fluticasone nasal spray in treatment of allergic rhinitis. International Journal of Otorhinolaryngology and Head and Neck Surgery. 2017.	not comparator of interest
4	Efficacy and safety of beclomethasone dipropionate nasal aerosol in children with perennial allergic rhinitis	Berger WE, Jacobs RL, Amar NJ, Tantry SK, Li J, Small CJ. Efficacy and safety of beclomethasone dipropionate nasal aerosol in children with perennial allergic rhinitis. Ann Allergy Asthma Immunol. 2015;115(2):130-6.	aerosol formulation of INCS
5	A comparison of fluticasone propionate nasal spray and cetirizine in ragweed fall seasonal allergic rhinitis	Kuna P, Wasiak W, Jones S, Kreft KZ. Comparative safety and efficacy of two formulations of mometasone nasal spray in adult seasonal allergic rhinitis. Allergy Asthma Proc. 2014;35(4):332-7.	not comparator of interest
6	Intranasal budesonide in children affected by persistent allergic rhinitis and its effect on nasal patency and Nasal Obstruction Symptom Evaluation (NOSE) score	Zicari AM, Occasi F, Montanari G, Indinnimeo L, De Castro G, Tancredi G, et al. Intranasal budesonide in children affected by persistent allergic rhinitis and its effect on nasal patency and Nasal Obstruction Symptom Evaluation (NOSE) score. Curr Med Res Opin. 2015;31(3):391-6.	not comparator of interest

No.	Studies	References	Reasons for exclusion
7	Long-term efficacy and safety of once-daily treatment with beclomethasone dipropionate nasal aerosol	Weinstein SF, Andrews CP, Shah SR, Chylack LT, Jr., Tankelevich A, Ding Y, et al. Long-term efficacy and safety of once-daily treatment with beclomethasone dipropionate nasal aerosol. Allergy Asthma Proc. 2014;35(4):323-31.	aerosol formulation of INCS
8	Efficacy and safety of fluticasone furoate nasal spray in Japanese children with perennial allergic rhinitis: a multicentre, randomized, double-blind, placebo-controlled trial	Okubo K, Okamasa A, Honma G, Komatsubara M. Efficacy and safety of fluticasone furoate nasal spray in Japanese children with perennial allergic rhinitis: a multicentre, randomized, double-blind, placebo-controlled trial. Allergol Int. 2014;63(4):543-51.	duration not consistent with inclusion criteria
9	Comparative safety and efficacy of two formulations of mometasone nasal spray in adult seasonal allergic rhinitis	Kuna P, Wasiak W, Jones S, Kreft KZ. Comparative safety and efficacy of two formulations of mometasone nasal spray in adult seasonal allergic rhinitis. Allergy Asthma Proc. 2014;35(4):332-7.	not comparator of interest
10	A pilot study of the effects of intranasal budesonide delivered by NasoNeb® on patients with perennial allergic rhinitis	Brown K, Lane J, Silva MP, DeTineo M, Naclerio RM, Baroody FM. A pilot study of the effects of intranasal budesonide delivered by NasoNeb® on patients with perennial allergic rhinitis. Int Forum Allergy Rhinol. 2014;4(1):43-8.	old device
11	Multicenter, double-blind, randomized, placebo-controlled study on mometasone furoate nasal spray in Japanese pediatric subjects with perennial allergic rhinitis	Okamoto Y, Suzuki I. Multicenter, Double-blind, Randomized, Placebo-controlled Study on Mometasone Furoate Nasal Spray in Japanese Pediatric Subjects with Perennial Allergic Rhinitis. 耳鼻咽喉科臨床　補冊. 2014;138:34-6.	duration not consistent with inclusion criteria
12	Efficacy and safety of beclomethasone dipropionate nasal aerosol in pediatric patients with seasonal allergic rhinitis	Storms WW, Segall N, Mansfield LE, Amar NJ, Kelley L, Ding Y, et al. Efficacy and safety of beclomethasone dipropionate nasal aerosol in pediatric patients with seasonal allergic rhinitis. Ann Allergy Asthma Immunol. 2013;111(5):408-14.e1.	dose ranging study

No.	Studies	References	Reasons for exclusion
13	Efficacy, safety, and optimal dose selection of beclomethasone dipropionate nasal aerosol for seasonal allergic rhinitis in adolescents and adults	Raphael GD, Berger WE, Prenner BM, Finn AF, Jr., Kelley L, Tantry SK. Efficacy, safety, and optimal dose selection of beclomethasone dipropionate nasal aerosol for seasonal allergic rhinitis in adolescents and adults. Curr Med Res Opin. 2013;29(10):1329-40.	dose ranging study
14	An integrated analysis of the efficacy of fluticasone furoate nasal spray versus placebo on the nasal symptoms of perennial allergic rhinitis	Wu W, Walters RD, Nadeau GA, Botnick W, Broughton N. An integrated analysis of the efficacy of fluticasone furoate nasal spray versus placebo on the nasal symptoms of perennial allergic rhinitis. Allergy Asthma Proc. 2013;34(3):283-91.	integrated analysis
15	Ocular safety of fluticasone furoate nasal spray in patients with perennial allergic rhinitis: A 2-year study	LaForce C, Journeay GE, Miller SD, Silvey MJ, Wu W, Lee LA, et al. Ocular safety of fluticasone furoate nasal spray in patients with perennial allergic rhinitis: a 2-year study. Ann Allergy Asthma Immunol. 2013;111(1):45-50.	not outcome of interest
16	Relieving nasal congestion in children with seasonal and perennial allergic rhinitis: Efficacy and safety studies of mometasone furoate nasal spray	Meltzer EO, Baena-Cagnani CE, Gates D, Teper A. Relieving nasal congestion in children with seasonal and perennial allergic rhinitis: efficacy and safety studies of mometasone furoate nasal spray. World Allergy Organ J. 2013;6(1):5.	post hoc analysis
17	Efficacy and safety of once-daily treatment with beclomethasone dipropionate nasal aerosol in subjects with seasonal allergic rhinitis	van Bavel JH, Ratner PH, Amar NJ, Hampel FC, Jr., Melchior A, Dunbar SA, et al. Efficacy and safety of once-daily treatment with beclomethasone dipropionate nasal aerosol in subjects with seasonal allergic rhinitis. Allergy Asthma Proc. 2012;33(5):386-96.	aerosol formulation of INCS
18	Safety and efficacy of once-daily treatment with beclomethasone dipropionate nasal	Meltzer EO, Jacobs RL, LaForce CF, Kelley CL, Dunbar SA, Tantry SK. Safety and efficacy of once-daily treatment with beclomethasone dipropionate nasal aerosol in subjects with	aerosol formulation of INCS

No.	Studies	References	Reasons for exclusion
	aerosol in subjects with perennial allergic rhinitis	perennial allergic rhinitis. Allergy Asthma Proc. 2012;33(3):249-57.	
19	A 26-week tolerability study of ciclesonide nasal aerosol in patients with perennial allergic rhinitis	Berger WE, Mohar DE, LaForce C, Raphael G, Desai SY, Huang H, et al. A 26-week tolerability study of ciclesonide nasal aerosol in patients with perennial allergic rhinitis. Am J Rhinol Allergy. 2012;26(4):302-7.	dose ranging study
20	A study of the efficacy and safety of ciclesonide hydrofluoroalkane nasal aerosol in patients with seasonal allergic rhinitis from mountain cedar pollen	Ratner PH, Andrews C, Martin B, Howland W, Desai SY, Huang H, et al. A study of the efficacy and safety of ciclesonide hydrofluoroalkane nasal aerosol in patients with seasonal allergic rhinitis from mountain cedar pollen. Allergy Asthma Proc. 2012;33(1):27-35.	dose ranging study, aerosol formulation of INCS
21	Efficacy and tolerability study of ciclesonide nasal aerosol in patients with perennial allergic rhinitis	Mohar D, Berger WE, Laforce C, Raphael G, Desai SY, Huang H, et al. Efficacy and tolerability study of ciclesonide nasal aerosol in patients with perennial allergic rhinitis. Allergy Asthma Proc. 2012;33(1):19-26.	dose ranging study
22	Efficacy of mometasone furoate nasal spray for nasal symptoms, quality of life, rhinitis-disturbed sleep, and nasal nitric oxide in patients with perennial allergic rhinitis	Yamada T, Yamamoto H, Kubo S, Sakashita M, Tokunaga T, Susuki D, et al. Efficacy of mometasone furoate nasal spray for nasal symptoms, quality of life, rhinitis-disturbed sleep, and nasal nitric oxide in patients with perennial allergic rhinitis. Allergy Asthma Proc. 2012;33(2):e9-16.	duration not consistent with inclusion criteria
23	Early interventional treatment with intranasal mometasone furoate in Japanese cedar/cypress pollinosis: a randomized placebo-controlled trial	Makihara S, Okano M, Fujiwara T, Kimura M, Higaki T, Haruna T, et al. Early interventional treatment with intranasal mometasone furoate in Japanese cedar/cypress pollinosis: a randomized placebo-controlled trial. Allergol Int. 2012;61(2):295-304.	mild AR

No.	Studies	References	Reasons for exclusion
24	Early interventional treatment with intranasal corticosteroids compared with postonset treatment in pollinosis	Higaki T, Okano M, Makihara S, Fujiwara T, Haruna T, Noda Y, et al. Early interventional treatment with intranasal corticosteroids compared with postonset treatment in pollinosis. Ann Allergy Asthma Immunol. 2012;109(6):458-64.	not outcome of interest
25	Effects of intranasal mometasone furoate on itchy ear and palate in patients with seasonal allergic rhinitis	Bernstein DI, Teper A, Gopalan G, Gates D. Effects of intranasal mometasone furoate on itchy ear and palate in patients with seasonal allergic rhinitis. Ann Allergy Asthma Immunol. 2012;108(5):359-62.	not outcome of interest
26	Efficacy and safety of fluticasone furoate nasal spray in Chinese adult and adolescent subjects with intermittent or persistent allergic rhinitis	Han D, Liu S, Zhang Y, Wang J, Wang D, Kong W, et al. Efficacy and safety of fluticasone furoate nasal spray in Chinese adult and adolescent subjects with intermittent or persistent allergic rhinitis. Allergy Asthma Proc. 2011;32(6):472-81.	duration not consistent with inclusion criteria
27	Recruitment factors which affect the outcome of a seasonal allergic rhinitis trial	Sharma S, Vasnani R, De Tineo M, Du G, Pinto JM, Baroody FM, et al. Recruitment factors which affect the outcome of a seasonal allergic rhinitis trial. Allergy Asthma Proc. 2011;32(1):55-63.	not outcome of interest
28	Evaluation of the efficacy and safety of ciclesonide hydrofluoroalkane nasal aerosol, 80 or 160 μg once daily, for the treatment of seasonal allergic rhinitis	Ratner P, Jacobs R, Mohar D, Huang H, Desai SY, Hinkle J. Evaluation of the efficacy and safety of ciclesonide hydrofluoroalkane nasal aerosol, 80 or 160 μg once daily, for the treatment of seasonal allergic rhinitis. Ann Allergy Asthma Immunol. 2010;105(6):471-9.	dose ranging study
29	Mometasone furoate nasal spray provides early, continuing relief of nasal congestion and improves nasal patency in allergic patients	Salapatek AM, Patel P, Gopalan G, Varghese ST. Mometasone furoate nasal spray provides early, continuing relief of nasal congestion and improves nasal patency in allergic patients. Am J Rhinol Allergy. 2010;24(6):433-8.	duration not consistent with inclusion criteria

No.	Studies	References	Reasons for exclusion
30	An integrated analysis of the efficacy of fluticasone furoate nasal spray on individual nasal and ocular symptoms of seasonal allergic rhinitis	Maspero JF, Walters RD, Wu W, Philpot EE, Naclerio RM, Fokkens WJ. An integrated analysis of the efficacy of fluticasone furoate nasal spray on individual nasal and ocular symptoms of seasonal allergic rhinitis. Allergy Asthma Proc. 2010;31(6):483-92.	integrated analysis
31	Comparison of patient preference for sensory attributes of fluticasone furoate or fluticasone propionate in adults with seasonal allergic rhinitis: a randomized, placebo-controlled, double-blind study	Meltzer EO, Andrews C, Journeay GE, Lim J, Prillaman BA, Garris C, et al. Comparison of patient preference for sensory attributes of fluticasone furoate or fluticasone propionate in adults with seasonal allergic rhinitis: a randomized, placebo-controlled, double-blind study. Ann Allergy Asthma Immunol. 2010;104(4):331-8.	not outcome of interest
32	Efficacy and safety of ciclesonide hydrofluoroalkane nasal aerosol once daily for the treatment of seasonal allergic rhinitis	LaForce C, van Bavel J, Meltzer EO, Wingertzahn MA. Efficacy and safety of ciclesonide hydrofluoroalkane nasal aerosol once daily for the treatment of seasonal allergic rhinitis. Ann Allergy Asthma Immunol. 2009;103(2):166-73.	dose ranging study
33	Efficacy and safety of once-daily fluticasone furoate nasal spray in children with seasonal allergic rhinitis treated for 2 wk	Meltzer EO, Lee J, Tripathy I, Lim J, Ellsworth A, Philpot E. Efficacy and safety of once-daily fluticasone furoate nasal spray in children with seasonal allergic rhinitis treated for 2 wk. Pediatr Allergy Immunol. 2009;20(3):279-86.	dose ranging study
34	Safety and tolerability of fluticasone furoate nasal spray once daily in paediatric patients aged 6-11 years with allergic rhinitis: Subanalysis of three randomized, double-blind, placebo-controlled, multicentre studies	Meltzer EO, Tripathy I, Máspero JF, Wu W, Philpot E. Safety and tolerability of fluticasone furoate nasal spray once daily in paediatric patients aged 6-11 years with allergic rhinitis: subanalysis of three randomized, double-blind, placebo-controlled, multicentre studies. Clin Drug Investig. 2009;29(2):79-86.	dose ranging study

No.	Studies	References	Reasons for exclusion
35	Onset of action of ciclesonide once daily in the treatment of seasonal allergic rhinitis	Couroux P, Kunjibettu S, Hall N, Wingertzahn MA. Onset of action of ciclesonide once daily in the treatment of seasonal allergic rhinitis. Ann Allergy Asthma Immunol. 2009;102(1):62-8.	not outcome of interest
36	Dose-ranging study of fluticasone furoate nasal spray for Japanese patients with perennial allergic rhinitis	Okubo K, Nakashima M, Miyake N, Uchida J, Okuda M. Dose-ranging study of fluticasone furoate nasal spray for Japanese patients with perennial allergic rhinitis*. Curr Med Res Opin. 2008;24(12):3393-403.	dose ranging study
37	Efficacy and safety of once-daily ciclesonide nasal spray in children with allergic rhinitis	Berger WE, Nayak A, Lanier BQ, Kaiser HB, LaForce C, Darken P, et al. Efficacy and Safety of Once-Daily Ciclesonide Nasal Spray in Children With Allergic Rhinitis. Pediatric Asthma, Allergy & Immunology. 2008;21(2):73-82.	dose ranging study
38	Safety and efficacy of fluticasone furoate in pediatric patients with perennial allergic rhinitis	Máspero JF, Rosenblut A, Finn A, Jr., Lim J, Wu W, Philpot E. Safety and efficacy of fluticasone furoate in pediatric patients with perennial allergic rhinitis. Otolaryngol Head Neck Surg. 2008;138(1):30-7.	dose ranging study
39	Fluticasone furoate versus placebo in symptoms of grass-pollen allergic rhinitis induced by exposure in the Vienna Challenge Chamber	Zieglmayer P, Zieglmayer R, Bareille P, Rousell V, Salmon E, Horak F. Fluticasone furoate versus placebo in symptoms of grass-pollen allergic rhinitis induced by exposure in the Vienna Challenge Chamber. Curr Med Res Opin. 2008;24(6):1833-40.	duration not consistent with inclusion criteria
40	Onset of action of ciclesonide once daily in the treatment of seasonal allergic rhinitis	Patel P, Patel D, Kunjibettu S, Hall N, Wingertzahn MA. Onset of action of ciclesonide once daily in the treatment of seasonal allergic rhinitis. Ear Nose Throat J. 2008;87(6):340-53.	not outcome of interest
41	Intranasal mometasone furoate alleviates the ocular symptoms associated with seasonal	Anolik R, Nathan RA, Schenkel E, Danzig MR, Gates D, Varghese S. Intranasal mometasone furoate alleviates the ocular symptoms	post hoc analysis

No.	Studies	References	Reasons for exclusion
	allergic rhinitis: Results of a post hoc analysis	associated with seasonal allergic rhinitis: results of a post hoc analysis. Int Arch Allergy Immunol. 2008;147(4):323-30.	
42	Ocular symptom reduction in patients with seasonal allergic rhinitis treated with the intranasal corticosteroid mometasone furoate	Bielory L. Ocular symptom reduction in patients with seasonal allergic rhinitis treated with the intranasal corticosteroid mometasone furoate. Ann Allergy Asthma Immunol. 2008;100(3):272-9.	retrospective pooled analysis
43	Optimal dose selection of fluticasone furoate nasal spray for the treatment of seasonal allergic rhinitis in adults and adolescents	Martin BG, Ratner PH, Hampel FC, Andrews CP, Toler T, Wu W, et al. Optimal dose selection of fluticasone furoate nasal spray for the treatment of seasonal allergic rhinitis in adults and adolescents. Allergy Asthma Proc. 2007;28(2):216-25.	dose ranging study
44	Safety of once-daily ciclesonide nasal spray in children 2 to 5 years of age with perennial allergic rhinitis	Kim K, Weiswasser M, Nave R, Ratner P, Nayak A, Herron J, et al. Safety of Once-Daily Ciclesonide Nasal Spray in Children 2 to 5 Years of Age with Perennial Allergic Rhinitis. Pediatric Asthma, Allergy & Immunology. 2007;20(4):229-42.	dose ranging study
45	Effectiveness of ciclesonide nasal spray in the treatment of seasonal allergic rhinitis	Ratner PH, Wingertzahn MA, van Bavel JH, Hampel F, Darken PF, Hellbardt S, et al. Effectiveness of ciclesonide nasal spray in the treatment of seasonal allergic rhinitis. Ann Allergy Asthma Immunol. 2006;97(5):657-63.	dose ranging study
46	Effects of intranasal corticosteroid on nasal adenosine monophosphate challenge in persistent allergic rhinitis	Barnes ML, Biallosterski BT, Fujihara S, Gray RD, Fardon TC, Lipworth BJ. Effects of intranasal corticosteroid on nasal adenosine monophosphate challenge in persistent allergic rhinitis. Allergy. 2006;61(11):1319-25.	not outcome of interest
47	Treatment with intranasal fluticasone propionate significantly improves ocular	Bernstein DI, Levy AL, Hampel FC, Baidoo CA, Cook CK, Philpot EE, et al. Treatment with intranasal fluticasone propionate	mild AR

No.	Studies	References	Reasons for exclusion
	symptoms in patients with seasonal allergic rhinitis	significantly improves ocular symptoms in patients with seasonal allergic rhinitis. Clin Exp Allergy. 2004;34(6):952-7.	
48	Triamcinolone acetonide and fluticasone propionate nasal sprays provide comparable relief of seasonal allergic rhinitis symptoms regardless of disease severity	Kaiser HB, Liao Y, Diener P, Leahy MJ, Garcia J, Georges G. Triamcinolone acetonide and fluticasone propionate nasal sprays provide comparable relief of seasonal allergic rhinitis symptoms regardless of disease severity. Allergy Asthma Proc. 2004;25(6):423-8.	post hoc analysis
49	Fluticasone propionate aqueous nasal spray improves nasal symptoms of seasonal allergic rhinitis when used as needed (prn)	Dykewicz MS, Kaiser HB, Nathan RA, Goode-Sellers S, Cook CK, Witham LA, et al. Fluticasone propionate aqueous nasal spray improves nasal symptoms of seasonal allergic rhinitis when used as needed (prn). Ann Allergy Asthma Immunol. 2003;91(1):44-8.	not daily use of drugs
50	A randomized comparison of the effects of budesonide and mometasone furoate aqueous nasal sprays on nasal peak flow rate and symptoms in perennial allergic rhinitis	Bende M, Carrillo T, Vóna I, da Castel-Branco MG, Arheden L. A randomized comparison of the effects of budesonide and mometasone furoate aqueous nasal sprays on nasal peak flow rate and symptoms in perennial allergic rhinitis. Ann Allergy Asthma Immunol. 2002;88(6):617-23.	mild AR
51	Dose-dependent effects of budesonide aqueous nasal spray on symptoms in a daily nasal allergen challenge model	Andersson M, Svensson C, Persson C, Akerlund A, Greiff L. Dose-dependent effects of budesonide aqueous nasal spray on symptoms in a daily nasal allergen challenge model. Ann Allergy Asthma Immunol. 2000;85(4):279-83.	dose ranging study
52	Onset of action of intranasal budesonide (Rhinocort Aqua) in seasonal allergic rhinitis studied in a controlled exposure model	Day JH, Briscoe MP, Rafeiro E, Ellis AK, Pettersson E, Akerlund A. Onset of action of intranasal budesonide (Rhinocort aqua) in seasonal allergic rhinitis studied in a controlled exposure model. J Allergy Clin Immunol. 2000;105(3):489-94.	not outcome of interest

No.	Studies	References	Reasons for exclusion
53	A dose-ranging study of mometasone furoate aqueous nasal spray in children with seasonal allergic rhinitis	Meltzer EO, Berger WE, Berkowitz RB, Bronsky EA, Dvorin DJ, Finn AF, et al. A dose-ranging study of mometasone furoate aqueous nasal spray in children with seasonal allergic rhinitis. J Allergy Clin Immunol. 1999;104(1):107-14.	dose ranging study
54	Mometasone furcate nasal spray is rapidly effective in the treatment of seasonal allergic rhinitis in an outdoor (park), acute exposure setting	Berkowitz RB, Roberson S, Zora J, Capano D, Chen R, Lutz C, et al. Mometasone furoate nasal spray is rapidly effective in the treatment of seasonal allergic rhinitis in an outdoor (park), acute exposure setting. Allergy Asthma Proc. 1999;20(3):167-72.	duration not consistent with inclusion criteria
55	Randomized Placebo-controlled Study Comparing a Leukotriene Receptor Antagonist and a Nasal Glucocorticoid in Seasonal Allergic Rhinitis	Pullerits T, Praks L, Skoogh BE, Ani R, Lötvall J. Randomized placebo-controlled study comparing a leukotriene receptor antagonist and a nasal glucocorticoid in seasonal allergic rhinitis. Am J Respir Crit Care Med. 1999;159(6):1814-8.	mild AR
56	Demonstration of therapeutic equivalence of generic and innovator beclomethasone in seasonal allergic rhinitis. SAR Study Group	Casale TB, Azzam SM, Miller RE, Oren J. Demonstration of therapeutic equivalence of generic and innovator beclomethasone in seasonal allergic rhinitis. SAR Study Group. Ann Allergy Asthma Immunol. 1999;82(5):435-41.	not comparator of interest
57	Onset of action of mometasone furoate nasal spray (NASONEX®) in seasonal allergic rhinitis	Berkowitz RB, Bernstein DI, LaForce C, Pedinoff AJ, Rooklin AR, Damaraju CR, et al. Onset of action of mometasone furoate nasal spray (NASONEX) in seasonal allergic rhinitis. Allergy. 1999;54(1):64-9.	not outcome of interest
58	Clinical and antiinflammatory effects of intranasal budesonide aqueous pump spray in the treatment of perennial allergic rhinitis	Meltzer EO. Clinical and antiinflammatory effects of intranasal budesonide aqueous pump spray in the treatment of perennial allergic rhinitis. Ann Allergy Asthma Immunol. 1998;81(2):128-34.	dose ranging study

No.	Studies	References	Reasons for exclusion
59	Intranasal budesonide aqueous pump spray (rhinocort® aqua) for the treatment of seasonal allergic rhinitis	Creticos P, Fireman P, Settipane G, Bernstein D, Casale T, Schwartz H. Intranasal budesonide aqueous pump spray (Rhinocort Aqua) for the treatment of seasonal allergic rhinitis. Rhinocort Aqua Study Group. Allergy Asthma Proc. 1998;19(5):285-94.	dose ranging study
60	Comparison of the efficacy of budesonide and fluticasone propionate aqueous nasal spray for once daily treatment of perennial allergic rhinitis	Day J, Carrillo T. Comparison of the efficacy of budesonide and fluticasone propionate aqueous nasal spray for once daily treatment of perennial allergic rhinitis. J Allergy Clin Immunol. 1998;102(6 Pt 1):902-8.	mild AR
61	Nasal budesonide offers superior symptom relief in perennial allergic rhinitis in comparison to nasal azelastine	Stern MA, Wade AG, Ridout SM, Cambell LM. Nasal budesonide offers superior symptom relief in perennial allergic rhinitis in comparison to nasal azelastine. Ann Allergy Asthma Immunol. 1998;81(4):354-8.	mild AR
62	A 1-year placebo-controlled study of intranasal Fluticasone Propionate Aqueous Nasal Spray in patients with perennial allergic rhinitis: a safety and biopsy study	Holm AF, Fokkens WJ, Godthelp T, Mulder PG, Vroom TM, Rijntjes E. A 1-year placebo-controlled study of intranasal fluticasone propionate aqueous nasal spray in patients with perennial allergic rhinitis: a safety and biopsy study. Clin Otolaryngol Allied Sci. 1998;23(1):69-73.	mild AR
63	Budesonide aqueous nasal spray and pressurized metered dose inhaler in the treatment of adult patients with seasonal allergic rhinitis	Day J, Alexander M, Drouin M, Frankish C, Mazza J, Moote W, et al. Budesonide aqueous nasal spray and pressurized metered dose inhaler in the treatment of adult patients with seasonal allergic rhinitis. Am J Rhinol. 1997;11(1):77-83.	dose ranging study
64	Dose ranging study of mometasone furoate (Nasonex) in seasonal allergic rhinitis	Bronsky EA, Aaronson DW, Berkowitz RB, Chervinsky P, Graft D, Kaiser HB, et al. Dose ranging study of mometasone furoate (Nasonex) in seasonal allergic rhinitis. Ann Allergy Asthma Immunol. 1997;79(1):51-6.	dose ranging study

No.	Studies	References	Reasons for exclusion
65	A comparison of aqueous suspensions of budesonide nasal spray (128 microg and 256 microg once daily) and fluticasone propionate nasal spray (200 microg once daily) in the treatment of adult patients with seasonal allergic rhinitis	Stern MA, Dahl R, Nielsen LP, Pedersen B, Schrewelius C. A comparison of aqueous suspensions of budesonide nasal spray (128 micrograms and 256 micrograms once daily) and fluticasone propionate nasal spray (200 micrograms once daily) in the treatment of adult patients with seasonal allergic rhinitis. Am J Rhinol. 1997;11(4):323-30.	mild AR
66	Long-term safety and efficacy of triamcinolone acetonide aqueous nasal spray for the treatment of perennial allergic rhinitis	Koepke JW, Beaucher WN, Kobayashi RH, Ransom JH, Rosen JP, Feiss G, et al. Long-term safety and efficacy of triamcinolone acetonide aqueous nasal spray for the treatment of perennial allergic rhinitis. Allergy Asthma Proc. 1997;18(1):33-7.	open-label extension study of Kobayashi et al., 1995 (Triamcinolone acetonide aqueous nasal spray for the treatment of patients with perennial allergic rhinitis: a multicenter, randomized, double-blind, placebo-controlled study)
67	Efficacy of triamcinolone acetonide aerosol nasal inhaler in children with perennial allergic rhinitis	Storms WW, Southern DL, Feiss G, Simpson B, Furst JA, Smith JA. Efficacy of Triamcinolone Acetonide Aerosol Nasal Inhaler in Children with Perennial Allergic Rhinitis. Pediatric Asthma, Allergy & Immunology. 1996;10(2):59-64.	aerosol formulation of INCS
68	Double-strength beclomethasone dipropionate (84 µg/spray) aqueous nasal spray in the treatment of seasonal allergic rhinitis	Prenner BM, Chervinsky P, Hampel FC, Jr., Howland WC, Lawrence M, Meltzer EO, et al. Double-strength beclomethasone dipropionate (84 μg/spray) aqueous nasal spray in the treatment of seasonal allergic rhinitis. Journal of Allergy and Clinical Immunology. 1996;98(2):302-8.	dose ranging study

No.	Studies	References	Reasons for exclusion
69	The efficacy of fluticasone propionate aqueous nasal spray for allergic rhinitis and its relationship to topical effects	Howland WC, 3rd, Hampel FC, Jr., Martin BG, Ratner PH, van Bavel JH, Field EA. The efficacy of fluticasone propionate aqueous nasal spray for allergic rhinitis and its relationship to topical effects. Clin Ther. 1996;18(6):1106-17.	dose ranging study
70	Efficacy and safety of triamcinolone acetonide aqueous nasal spray in patients with seasonal allergic rhinitis	Munk ZM, LaForce C, Furst JA, Simpson B, Feiss G, Smith JA. Efficacy and safety of triamcinolone acetonide aqueous nasal spray in patients with seasonal allergic rhinitis. Ann Allergy Asthma Immunol. 1996;77(4):277-81.	mild AR
71	A placebo- and active-controlled randomized trial of prophylactic treatment of seasonal allergic rhinitis with mometasone furoate aqueous nasal spray	Graft D, Aaronson D, Chervinsky P, Kaiser H, Melamed J, Pedinoff A, et al. A placebo- and active-controlled randomized trial of prophylactic treatment of seasonal allergic rhinitis with mometasone furoate aqueous nasal spray. J Allergy Clin Immunol. 1996;98(4):724-31.	mild AR
72	A placebo-controlled study of fluticasone propionate aqueous nasal spray and beclomethasone dipropionate in perennial rhinitis: efficacy in allergic and non-allergic perennial rhinitis	Scadding GK, Lund VJ, Jacques LA, Richards DH. A placebo-controlled study of fluticasone propionate aqueous nasal spray and beclomethasone dipropionate in perennial rhinitis: efficacy in allergic and non-allergic perennial rhinitis. Clin Exp Allergy. 1995;25(8):737-43.	dose ranging study
73	The efficacy and tolerability of fluticasone propionate aqueous nasal spray in children with seasonal allergic rhinitis	Boner A, Sette L, Martinati L, Sharma RK, Richards DH. The efficacy and tolerability of fluticasone propionate aqueous nasal spray in children with seasonal allergic rhinitis. Allergy. 1995;50(6):498-505.	dose ranging study
74	Onset of action of aqueous beclomethasone dipropionate nasal spray in seasonal allergic rhinitis	Selner JC, Weber RW, Richmond GW, Stricker WE, Norton JD. Onset of action of aqueous beclomethasone dipropionate nasal spray in seasonal allergic rhinitis. Clin Ther. 1995;17(6):1099-109.	not outcome of interest

No.	Studies	References	Reasons for exclusion
75	Treatment of seasonal allergic rhinitis with once-daily intranasal fluticasone propionate therapy in children	Galant SP, Ahrens, R. C., Dockhorn, R. J., Lumry, W., Martin, B., Meltzer, E. O., Munk, Z., Ransom, J., Southern, D. L., Stricker, W. E., Field, E. A., Rogenes, P. R., Kral, K. M. Treatment of seasonal allergic rhinitis with once-daily intranasal fluticasone propionate therapy in children. The Journal of Pediatrics. 1994;125(4):628-34.	dose ranging study
76	Fluticasone propionate - an effective alternative treatment for seasonal allergic rhinitis in adults and adolescents	LaForce CF, Dockhorn RJ, Findlay SR, Meltzer EO, Nathan RA, Stricker W, et al. Fluticasone propionate: an effective alternative treatment for seasonal allergic rhinitis in adults and adolescents. J Fam Pract. 1994;38(2):145-52.	dose ranging study
77	Intranasal Fluticasone Propionate Is Effective and Well-Tolerated in Adolescents with Seasonal Allergic Rhinitis	Munk ZM, Pearlman D, Graft D, Green A, Hampel F, Pleskow W, et al. Intranasal Fluticasone Propionate Is Effective and Well-Tolerated in Adolescents with Seasonal Allergic Rhinitis. Pediatric Asthma, Allergy & Immunology. 1994;8(1):39-46.	dose ranging study
78	A double-blind comparison of fluticasone propionate aqueous nasal spray, terfenadine tablets and placebo in the treatment of patients with seasonal allergic rhinitis to grass pollen	Darnell R, Pecoud A, Richards DH. A double-blind comparison of fluticasone propionate aqueous nasal spray, terfenadine tablets and placebo in the treatment of patients with seasonal allergic rhinitis to grass pollen. Clin Exp Allergy. 1994;24(12):1144-50.	not outcome of interest
79	Once-daily versus twice-daily fluticasone propionate aqueous nasal spray for seasonal allergic rhinitis	Dockhorn RJ, Paull BR, Meltzer EO, van As A, Weakley S, Woehler TR, et al. Once- versus Twice-Daily Fluticasone Propionate Aqueous Nasal Spray for Seasonal Allergic Rhinitis. American Journal of Rhinology. 1993;7(2):77-83.	dose ranging study
80	Once daily fluticasone propionate is as effective for perennial allergic rhinitis as twice daily beclomethasone diproprionate	van As A, Bronsky EA, Dockhorn RJ, Grossman J, Lumry W, Meltzer EO, et al. Once daily fluticasone propionate is as effective	mild AR

No.	Studies	References	Reasons for exclusion
		for perennial allergic rhinitis as twice daily beclomethasone diproprionate. J Allergy Clin Immunol. 1993;91(6):1146-54.	
81	Nasal beclomethasone prevents the seasonal increase in bronchial responsiveness in patients with allergic rhinitis and asthma	Corren J, Adinoff AD, Buchmeier AD, Irvin CG. Nasal beclomethasone prevents the seasonal increase in bronchial responsiveness in patients with allergic rhinitis and asthma. J Allergy Clin Immunol. 1992;90(2):250-6.	mild AR
82	Once daily FP aqueous nasal spray is an effective treatment for seasonal allergic rhinitis	Nathan RA, Bronsky EA, Fireman P, Grossman J, LaForce CF, Lemanske RF, Jr., et al. Once daily fluticasone propionate aqueous nasal spray is an effective treatment for seasonal allergic rhinitis. Ann Allergy. 1991;67(3):332-8.	mild AR
83	A dose-ranging study of fluticasone propionate aqueous nasal spray for seasonal allergic rhinitis assessed by symptoms, rhinomanometry, and nasal cytology	Meltzer EO, Orgel HA, Bronsky EA, Furukawa CT, Grossman J, LaForce CF, et al. A dose-ranging study of fluticasone propionate aqueous nasal spray for seasonal allergic rhinitis assessed by symptoms, rhinomanometry, and nasal cytology. J Allergy Clin Immunol. 1990;86(2):221-30.	dose ranging study
84	Evaluation of Flunisolide Nasal Solution in the Symptomatic Treatment of Perennial Rhinitis	Warland A. Evaluation of flunisolide nasal solution in the symptomatic treatment of perennial rhinitis. Allergy. 1982;37(6):417-20.	mild AR
85	Treatment of Seasonal and Perennial Rhinitis with Intranasal Flunisolide	Turkeltaub PC, Norman PS, Johnson JD, Crepea S. Treatment of seasonal and perennial rhinitis with intranasal flunisolide. Allergy. 1982;37(5):303-11.	mild AR
86	Short-term efficacy trial and twenty-four-month follow-up of flunisolide nasal spray in the treatment of perennial rhinitis	Clayton DE, Kooistra JB, Geller M, Ouellette J, Cohen M, Reed CE, et al. Short-term efficacy trial and twenty-four-month follow-up of flunisolide nasal spray in the treatment of perennial rhinitis. J Allergy Clin Immunol. 1981;67(1):2-7.	Supratherapeutic dose (300 mcg of Flunisolide)

No.	Studies	References	Reasons for exclusion
87	Flunisolide—a new intranasal steroid for the treatment of allergic rhinitis	Sahay JN, Chatterjee SS, Engler C. Flunisolide--a new intranasal steroid for the treatment of allergic rhinitis. Clin Allergy. 1979;9(1):17-24.	duration not consistent with inclusion criteria
88	Clinical evaluation of intranasal topical flunisolide therapy in allergic rhinitis	Kammermeyer JK, Rajtora DW, Anuras J, Richerson HB. Clinical evaluation of intranasal topical flunisolide therapy in allergic rhinitis. J Allergy Clin Immunol. 1977;59(4):287-93.	dose ranging study
89	The nasal application of beclomethasone diproprionate (Beconase) in allergic rhinitis	Rudolph R, Kunkel G, Staud RD, Koennecke R. The nasal application of beclomethasone diproprionate (Beconase) in allergic rhinitis. Clin Otolaryngol Allied Sci. 1976;1(4):315-23.	mild AR

Supplementary Table S3. Inclusion criteria, exclusion criteria, and intervention withdrawal of included studies

Study	Inclusion criteria	Exclusion criteria	Name of INCS and no. of patients	No. of withdrawal (n, %)	Reasons for withdrawal (n)
Ratner (2015)	• Patients ≥12 yr with a history of AR for ≥2 yr<br>• Patients with allergic conjunctivitis symptoms of at least moderate severity with an iTOSS of ≥4 and an instantaneous nasal congestion symptom score ≥ 2 in the morning of randomization and a mean iTOSS ≥4 and an instantaneous nasal congestion symptom score ≥2 for 3 of the 5 days during the placebo lead-in	• Pregnant or breastfeeding women<br>• Patients with suspected intolerance or hypersensitivity to the study materials or with nasal disorders that investigators believed would have interfered with participation in the study<br>• Patients with significant uncontrolled diseases, such as respiratory disease, cardiac arrhythmias, congestive heart failure, malignancy, diabetes mellitus, or hypertension.<br>• Patients with current infection, impairments in learning, long-term or intermittent use of corticosteroids, and intended travel outside the geographic region for >48 hours	FP 200 µg OD (314)<br>Placebo (312)	12 (1.9%)	• 2 (0.3%) - withdrew because of AEs<br>• 1 (0.2%) - lost to follow-up<br>• 3 (0.5%) - protocol violations (as identified by the investigator)<br>• 4 (0.6%) - withdrew consent<br>• 2 (0.3%) - withdrew for other reasons
Igarashi (2012)	• Male or female patients ≥15 yr with SAR from an ophthalmologist and an otorhinolaryngologist	• NR	18 patients in MF 200 µg OD and Placebo	7 (38.9%)	• 2 (11.1%) - for failing to attend the initial and subsequent consultations<br>• 1 (5.6%) - excluded for administration outside of the heavy pollen dispersal period<br>• 3 (16.7%) - excluded for using prohibited medications<br>• 1 (5.6%) - for failing to complete the allergy diary
Meltzer (2011)	• Subjects ≥ 12 yr with ≥2-yr history of SAR that exacerbated during the	• Patients with a history of severe local reaction(s) or anaphylaxis to SPT	MF 200 µg OD (344)	4 (1.2%)	• 2 (0.6%) - AEs<br>• 1 (0.3%) - treatment failure

Study	Inclusion criteria	Exclusion criteria	Name of INCS and no. of patients	No. of withdrawal (n, %)	Reasons for withdrawal (n)
	study season and having a positive SPT in response to an appropriate seasonal allergen at visit 1	• Using any prohibited medication, having a significant medical condition(s) that might interfere with the study or require treatment<br>• Patients with an upper respiratory tract or sinus infection that required antibiotic therapy without at least a 14-day washout before the screening visit, a viral URI within 7 days before the screening visit<br>• Pregnant or breastfeeding women			• 1 (0.3%) - personal reasons
			Placebo (340)	3 (0.9%)	• 1 (0.3%) - AEs<br>• 2 (0.6%) - treatment failure
Prenner (2010)	• Subjects ≥ 12 yr with ≥2-yr history of SAR that exacerbated during the study season and having a positive SPT in response to an appropriate seasonal allergen<br>• Clinically symptomatic (as assessed by diary records) at screening: rhinorrhea ≥2; nasal congestion ≥2; TNSS (sum of individual symptom scores for rhinorrhea, nasal congestion, nasal itching, and sneezing) ≥ 6, TOSS (sum of individual symptom scores for ocular itching, tearing/watering, and ocular redness) ≥ 4, and overall evaluation of SAR ≥2 (moderate)	• History of anaphylaxis, other severe local reactions, or both to SPT.<br>• Corticosteroid-dependent asthma<br>• Current or prior frequent, clinically significant sinusitis, or chronic, purulent postnasal drip<br>• Significant comorbid medical conditions/diseases that could interfere with the completion of study diaries, affect the metabolism of study medication, or require treatment<br>• Upper respiratory tract/sinus infection, requiring a course of antibiotics not complete within ≥ 14 d of the screening visit or viral URI ≤ 7 d before screening<br>• Rhinitis medicamentosa<br>• Nasal abnormalities that could interfere with nasal airflow<br>• Dependency on nasal, ocular, or oral decongestants; nasal antihistamines; or INCS<br>• History of allergies to ≥ 2 classes of medications or intolerance to nasal sprays	MF 200 µg OD (220)	9 (2%)	• 1 % - AEs<br>• 1 % - non-compliance
			Placebo (209)		• 0.48 % - AEs<br>• 0.48 % - non-compliance<br>• 0.48 % - treatment failure<br>• 0.48 % - consent withdrawal<br>• 0.48 % - protocol violation

Study	Inclusion criteria	Exclusion criteria	Name of INCS and no. of patients	No. of withdrawal (n, %)	Reasons for withdrawal (n)
		• Subjects receiving immunotherapy, unless on a regular maintenance schedule before screening and for the duration of the study<br>• Pregnant or breastfeeding women			
Okubo (2009)	• Patients ≥16 yr with >2-yr histories of SAR (Japanese cedar pollinosis)	• Patients with an existing nasal disorder that might interfere with evaluation (e.g., acute/chronic sinusitis, nasal polyposis, vasomotor rhinitis, or drµg-induced rhinitis) or a coexisting disease (e.g., serious hepatic/renal, cardiac, or pulmonary dysfunction; tuberculous diseases; systemic mycosis; hypertension; diabetes mellitus; nasal or oropharynx candidiasis; asthma [except mild intermittent asthma]; glaucoma; cataract; herpes simplex ophthalmic; or recurrent epistaxis<br>• Pregnant or lactating women<br>• Patients receiving corticosteroids within 8 weeks of the screening period or any other medication that could affect efficacy assessment	FP 200 µg OD (148)<br>FP placebo (75)<br>FF 110 µg OD (151)<br>FF placebo (72)	13 (2.9%)	• 8 (1.8%) - protocol deviations<br>• 2 (0.5%) - due to double enrollment<br>• 2 (0.5%) - lost allergy diary<br>• 1 (0.2%)- not receive the investigational product
Jacobs (2009)	• Patients ≥12 yr with SAR and a positive SPT for mountain cedar allergy within 12 months prior to study start<br>• Patients were required to be clinically symptomatic on the first day of treatment and to have attained a predefined minimum level of symptom severity to be eligible for randomization.	• Patients with significant concomitant medical conditions or an existing nasal disorder that might interfere with the study<br>• Using corticosteroids, including INCS within 4 weeks of visit 1 (screening visit), or inhaled, oral, intramuscular, intravenous, ocular, and/or topical corticosteroids (except for hydrocortisone cream/ointment 1% or less) within 8 weeks prior to visit 1.	FF 110 µg OD (152)<br>Placebo (150)	94% of patients completed the study	• 2 (0.7%) – AEs<br>• 4 (1.3%) - protocol violations

Study	Inclusion criteria	Exclusion criteria	Name of INCS and no. of patients	No. of withdrawal (n, %)	Reasons for withdrawal (n)
		• Patients who had used other allergy medications, such as short-acting prescription and over-the-counter antihistamines within 3 days, long-acting antihistamines within 10 days, intranasal antihistamines within 2 weeks, oral or intranasal decongestants, long-acting beta-agonists, intranasal, oral or inhaled anticholinergics, or oral antileukotrienes, all within 72 hours prior to visit 1<br>• Using medications that affect AR symptoms (e.g., tricyclic antidepressants, other intranasal medications, throat lozenges, immunosuppressive medications, and medications that significantly inhibit the cytochrome P450 subfamily enzyme CYP3A4 (including ritonavir and ketoconazole)			
Andrews (2009)	• Patients ≥12 yr with SAR in the last two respective allergy seasons and a positive SPT to mountain cedar allergen (study 1) or ragweed allergen (study 2) within 12 months<br>• Patients were expected to have adequate exposure to the respective pollen during the study (i.e., resided in a geographical region where exposure to mountain cedar [study 1] or ragweed [study	• Pregnant at study entry<br>• Patients with severely deviated septum or nasal polyp; recent nasal septal surgery or perforation; asthma, unless it was mild intermittent asthma; rhinitis medicamentosa; ocular or bacterial or viral URI; chronic use of medications that could affect AR or assessments of the efficacy of study medication; and current tobacco use.<br>• Using the following medications before screening: subcutaneous omalizumab within 5 months; corticosteroids (intranasal form within	FF 110 μg OD (312)	14 (4%)	• 2 (<1%) - AEs<br>• 1 (<1%) - Lost to follow-up<br>• 9 (3%) - Protocol violation<br>• 1 (<1%) - Lack of efficacy<br>• 1 (<1%) - Others
			Placebo (313)	23 (7%)	• 1 (<1%) - AEs<br>• 1 (<1%) - Lost to follow-up<br>• 6 (2%) - Protocol violation<br>• 1 (<1) - Patient decision

Study	Inclusion criteria	Exclusion criteria	Name of INCS and no. of patients	No. of withdrawal (n, %)	Reasons for withdrawal (n)
	2] was expected to be significant and had no plans to travel outside that region for > 48 hours of the study)	4 weeks and inhaled, intramuscular, intravenous and/or topical forms, except ≤ 1% hydrocortisone cream/ointment, within 8 weeks; short-acting prescription or over-the-counter antihistamines, oral or intranasal decongestants, anticholinergics, long-acting β-agonists, or oral antileukotrienes within 3 days; long-acting antihistamines within 10 days; and intranasal antihistamines or intranasal or ocular cromolyn within 14 days			• 2 (<1%) - Lack of efficacy<br>• 12 (4%) - Others
			FF 110 µg OD (224)	15 (7%)	• 2 (<1%) - AEs<br>• 5 (2%) - Protocol violation<br>• 1 (<1%) - Patient decision<br>• 7 (3%) - Others
			Placebo (229)	10 (4%)	• 1 (<1%) - AEs<br>• 1 (<1%) - Protocol violation<br>• 1 (<1%) - Patient decision<br>• 1 (<1%) - The sponsor terminated the study<br>• 6 (3%) - Others
Kaiser (2007)	• Patients ≥12 yr with a history of SAR caused by ragweed pollen<br>• Patients with moderate-to-severe nasal and ocular symptoms<br>• Patients with a mean daily rTNSS ≥6, a mean daily reflective nasal symptom score for congestion ≥2, and a mean daily rTOSS ≥4. All were measured over four 24-hour periods.	• Patients with significant concomitant medical conditions or uncontrolled diseases, severe physical nasal obstruction or injury, asthma, rhinitis medicamentosa, bacterial or viral infection within 2 weeks, acute or significant chronic sinusitis, glaucoma and/or cataracts or ocular herpes simplex, Candida infection of the nose, any psychiatric disorder, or adrenal insufficiency<br>• Using a systemic or inhaled corticosteroid within 8 weeks, any INCS within 4 weeks, other allergy medications within specified time frames chosen to ensure no continued effect on symptoms, or any other medications that could affect AR or the effectiveness of the study drug.	FF 110 µg OD (151)	2 (1.3%)	• 2 (1.3%) – AEs
			Placebo (148)	4 (2.7%)	• 2 (1.4%) - AEs<br>• 2 (1.4%) - lack of efficacy

Study	Inclusion criteria	Exclusion criteria	Name of INCS and no. of patients	No. of withdrawal (n, %)	Reasons for withdrawal (n)
		• Patients with contact lenses or the use of any ocular preparations (including artificial tears, eyewash/irrigation solutions, or lubricants)			
Fokkens (2007)	• Patients ≥ 12 yr with a history of SAR and either positive SPT or specific IgE to grass pollen within the 12 months prior to the study	• Patients with severe physical nasal injury or obstruction, asthma, rhinitis medicamentosa, or any other chronic medical condition that could interfere with the course of the study<br>• Patients who had received any INCS within 4 weeks, any other corticosteroid within 8 weeks, and any other medications that could affect the symptoms of SAR or the effectiveness of the study drµg	FF 110 µg OD (141)	3 (2%)	1 (1%) - lack of efficacy<br>2 (1.4%) - NR
			Placebo (144)	16 (11%)	9 (6%) - lack of efficacy<br>2 (1.4%) - severe seasonal allergy<br>5 (3.5%) - NR
Ratner (2006)	• Patients ≥12 yr with ≥2-yr history of SAR and a positive SPT to mountain cedar pollen	• Patients with nasal polyps within 60 days before study initiation or clinically relevant respiratory tract malformations; recent nasal biopsy within 60 days; nasal trauma; nasal surgery; atrophic rhinitis; rhinitis medicamentosa within 60 days; or active asthma requiring treatment with inhaled or systemic corticosteroids, routine use of β-agonists, or both<br>• Patients with hypersensitivity to corticosteroids, a history of respiratory tract infection or disorder within 14 days of the screening visit, respiratory tract infection during baseline, use of antibiotics for acute conditions within 14 days of the screening visit<br>• Patients who initiate immunotherapy or increase the dose of existing immunotherapy during the study	CIC 200 µg OD (164)	21 (12.8%)	4 (2.4%) - AEs<br>4 (2.4%) - lack of efficacy<br>13 (7.9%) - NR
			Placebo (163)	14 (8.6%)	5 (3.1%) - AEs<br>5 (3.1%) - lack of efficacy<br>4 (2.5%) - NR

Study	Inclusion criteria	Exclusion criteria	Name of INCS and no. of patients	No. of withdrawal (n, %)	Reasons for withdrawal (n)
Meltzer (2004)	• Patients 12–70 yr with ≥2-yr history of SAR and a positive SPT ≥1 following allergens in their environment: grass pollens, tree pollens, and/or outdoor molds	• Patients who had begun immunotherapy or had received short- or long-acting steroids (oral or parenteral), INCS, or nasal cromolyn within 30 days; had taken an antihistamine or antileukotrienes within 5 days of baseline visit<br>• Pregnant or lactating women<br>• Patients with a history of habitual use of nasal decongestants<br>• Patients who were hypersensitive or unresponsive to INCS<br>• Patients with unstable or severe asthma, sinusitis, or an underlying nasal pathology resulting in fixed occlusion of a nostril<br>• Patients with fungal infection of the nose, mouth, or throat; or used TAA or FP within 3 months before screening	TAA 220 µg OD (19)	NR	NR
			FP 200 µg OD (20)	NR	NR
Gawchik (2003)	• Patients ≥12 yr with a history of SAR-associated coµgh for ≥1 yr<br>• Patients who had to be free of any clinically significant disease other than SAR	• Patients with asthma exacerbations during pollen season or a pre-existing coµgh due to any etiology other than SAR<br>• Patients with clinically relevant symptoms of chest tightness, wheezing, shortness of breath<br>• Patients using short-acting inhaled β-agonists more than twice per week (excluding pre-exercise); or required daily or alternate-day oral corticosteroid for a total of more than 14 days during the 6 months before screening<br>• Patients who used INCS, inhaled, and oral steroids within the previous month	MF 200 µg OD (122)	0	0
			Placebo (123)	4 (3.3%)	• 3 (2.4%) - treatment failure<br>• 1 (0.8%) - protocol non-compliance

Study	Inclusion criteria	Exclusion criteria	Name of INCS and no. of patients	No. of withdrawal (n, %)	Reasons for withdrawal (n)
Lumry (2003)	• Patients ≥18 yr with a history of SAR during the preceding two years' ragweed pollen season requiring medication use and were considered candidates for treatment with INCS	• Patients with any significant physical abnormalities or abnormal laboratory values<br>• Nasal candidiasis, acute or chronic sinusitis, significant nasal polyposis or other deformities of the nose sufficient to impair nasal breathing<br>• Concurrent medical conditions likely to interfere with the course of the study<br>• Use of systemic corticosteroids in the previous 42 days or nasal or inhaled corticosteroids in the previous 30 days<br>• Use of nasal cromolyn sodium in the previous 28 days or astemizole in the previous 60 days<br>• Treatment with an investigational drμg within 60 days<br>• Commencement of immunotherapy within the previous six months<br>• Use of medication for other conditions that might produce or relieve the signs and symptoms of AR for six days prior to and throughout the treatment period<br>• Pregnancy, lactation, or inadequate contraceptive precautions in females of child-bearing potential	TAA 220 μg OD (75)<br>BDP 168 μg BID (77)	7 (4.6%)	• 1 (0.7%) - lost to follow-up<br>• 1 (0.7%) - protocol deviation<br>• 5 (3.3%) - unable to accurately rate their allergy symptoms secondary to concurrent illnesses
Berger (2003)	• Patients 12-70 yr with ≥2-yr history of spring SAR, characterized by nasal congestion, rhinorrhea, sneezing, and nasal itching	• Patients who had received short- or long-acting steroids (oral or parenteral), INCS, or nasal cromolyn within 30 days; had taken an antihistamine or antileukotriene within 5 days of baseline visit<br>• Pregnant or lactating women<br>• Patients with habitual use of nasal decongestants	TAA 220 μg OD (148)	5 (3.4%)	• No patient discontinued due to an AEs or for reasons related to study medication
			FP 200 μg OD (147)	3 (2%)	• No patient discontinued due to AEs or for reasons related to study medication

Study	Inclusion criteria	Exclusion criteria	Name of INCS and no. of patients	No. of withdrawal (n, %)	Reasons for withdrawal (n)
		• Patients who were hypersensitive or nonresponsive to INCS<br>• Patients with unstable or severe asthma<br>• Patients who had begun immunotherapy within 1 month of study initiation<br>• Patients who had sinusitis or an underlying nasal pathology resulting in fixed occlusion of a nostril<br>• Patients with fungal infection of the nose, mouth, or throat<br>• Patients who used TAA or FP within the 3 months before screening			
Gross (2002)	• Patients 12-70 yr with ≥ 2-yr history of fall (ragweed) AR	• Pregnant or lactating women<br>• Patients who had received any of the following within 42 days of the screening visit: an oral or parenteral short-acting steroid (excluding oral contraceptives and hormone replacement therapies such as estrogen), a long-acting steroid medication, INCS, nasal cromolyn, or astemizole<br>• Patients with abuse of nasal decongestants<br>• Patients who were hypersensitive or nonresponsive to INCS<br>• Patients with the initiation of immunotherapy within 1 month of study<br>• Patients with nasal pathology resulting in fixed occlusion of a nostril<br>• Patients who had the disease with the potential to interfere with the evaluation of study medications	TAA 220 µg OD (172)	5 (2.9%)	• 3 (1.7%) - protocol violations<br>• 2 (1.2%) - AEs
			FP 200 µg OD (180)	5 (2.8%)	• 4 (2.2%) - protocol violations<br>• 1 (0.6%) - AEs

Study	Inclusion criteria	Exclusion criteria	Name of INCS and no. of patients	No. of withdrawal (n, %)	Reasons for withdrawal (n)
		• Use of any medication that might independently affect the symptoms of SAR<br>• Patients with fungal infection in the nose, mouth, or throat			
Meltzer (1998)	• Patients 12-65 yr with ≥2-yr history of SAR<br>• Symptomatic patients with a positive SPT to the relevant allergens within the previous 14 months	• Patients with medical conditions or nasal abnormalities that would interfere with the study	MF 200 µg OD (85)	5 (5.9%)	• NR
			Placebo (43)	2 (4.7%)	• NR
Bronsky. (1996)	• Patients ≥12 yr with ≥1-yr history of moderate-to-severe SAR<br>• Patients with morning plasma cortisol concentration equal to or greater than 7 µg/dL	• Patients who received oral antihistamines or cromolyn sodium for ≥2 weeks before screening<br>• Patients who received astemizole or inhaled, intranasal, or systemic corticosteroids for ≥1 month before screening	FP 200 µg OD (117)	6 (5.1%)	• 1 (1%) - AEs<br>• 0 (0%) - Lack of efficacy<br>• 5 (4%) - noncompliance, loss to follow-up, protocol violation, and withdrawn consent
			Terfenadine 60 mg BID (116)	13 (11.2%)	• 2 (2%) - AEs<br>• 3 (3%) - Lack of efficacy<br>• 8 (7%) - noncompliance, loss to follow-up, protocol violation, and withdrawn consent
			Placebo (115)	10 (0.9%)	• 3 (3%) - AEs<br>• 3 (3%) - Lack of efficacy<br>• 4 (3%) - noncompliance, lost to follow-up, protocol violation, and withdrawn consent
van Bavel (1994)	• Patients ≥12 yr with moderate-to-severe SAR diagnosed according to three criteria: (1) appearance of nasal mucosa consistent with a diagnosis	• Patients who received oral antihistamines or cromolyn for at least 2 weeks, astemizole or inhaled, intranasal, or systemic steroids for at least 1 month before screening	FP 200 µg OD (78)	15 (19.2%)	• 4 (1.7%) - AEs<br>• 7 (3%) - (five in the placebo group, one in the fluticasone group, and one in the terfenadine group) - lack of efficacy
			Terfenadine 60 mg BID (77)		
			Placebo (77)		

Study	Inclusion criteria	Exclusion criteria	Name of INCS and no. of patients	No. of withdrawal (n, %)	Reasons for withdrawal (n)
	of SAR; (2) at least a 1-year history of symptoms with the onset and offset corresponding to the beginning and end of the mountain cedar season; and (3) positive SPT to mountain cedar antigen within 12 months<br>• Patients with morning plasma cortisol concentration of ≥7 µg/dL				• 3 (1.3%) - (one per treatment arm) - protocol violation<br>• 1 (0.4%) fluticasone-treated patient did not return for the follow-up visit
Ratner (1992)	• Adults with ≥2-yr history of moderate-to-severe SAR during the mountain cedar season<br>• Women of nonchildbearing potential<br>• Patients with normal adrenal function (morning plasma cortisol concentration of ≥7 µg/dL)	• Patients who had received oral, inhaled, or INCS within 1 month or intranasal cromolyn within 2 weeks of the study entry	FP 200 µg OD (106)<br>BDP 168 µg BID (103)<br>Placebo (104)	4 (1.3%)	• 1 (0.3%) - patient receiving active drµg withdrew for personal reasons<br>• 1 (0.3%) - using systemic corticosteroids.<br>• 2 (0.6%) - AEs
Karaulov (2019)	• Patients 18-50 yr with PAR and positive SPT to an appropriate allergen (house dust mites, molds, etc.)<br>• Patients who presented symptoms that lasted for more than 4 days a week and for more than 4 consecutive weeks<br>• Patients who had a rTNSS ≥8 within the past 24 h before the screening visit, with two or more symptoms rated as moderate or severe	• Patients with SAR, intermittent AR, non-AR caused by viral or bacterial infection, rhinitis medicamentosa, asthma, or chronic sinusitis<br>• Patients presenting with URI or sinus infections that required antibiotic therapy without at least a 14-day washout or presenting with viral upper respiratory infections within 2 weeks, and patients who received specific immunotherapy that ended within the 6 months prior to the screening visit<br>• Patients who had taken the following drugs within a specific period	TAA 220 µg OD (129)	1 (0.8%)	• 1 (0.8%) - discontinued the study prematurely
			FP 200 µg OD (131)	3 (2.3%)	• 2 (1.5%) - discontinued the study prematurely<br>• 1 (0.8%) - protocol deviation

Study	Inclusion criteria	Exclusion criteria	Name of INCS and no. of patients	No. of withdrawal (n, %)	Reasons for withdrawal (n)
		(respective to each drµg's washout period) prior to screening were excluded, namely: corticosteroids, cromones, short-acting and long-acting antihistamines, as well as intranasal antihistamines, anticholinergics (intranasal, oral, or inhaled), oral antileukotrienes, and immunosuppressive medications			
Meltzer (2010)	• Patients 18-60 yr with ≥ 2-y history of PAR with or without SAR and self-reported sleep disturbances<br>• Positive SPT to relevant perennial allergens in the previous 12 months<br>• Nasal congestion score ≥ 4 of 6 and TNSS ≥12 of 24 at screening (visit 1) and baseline (visit 2)<br>• Sleep disturbance symptoms and score ≥2 (moderate severity) on the Interference with Sleep Scale at screening (visit 1) and baseline (visit 2)<br>• ≥5 to ≤30 apnea and hypopnea events per hour during screening	• Current use of medication for PAR or treatment during the 10 days before screening with antihistamines or INCS<br>• Presence/history of clinically significant sinusitis, chronic purulent postnasal drip, rhinitis medicamentosa, or respiratory tract or sinus infection<br>• Nasal septum ulcers, nasal surgery, nasal trauma, or structural abnormalities, including nasal polyps and septum deviation, that significantly interfere with nasal airflow<br>• Asthma uncontrolled by short-acting β_2-adrenergic receptor agonists<br>• Morbid obesity (BMI ≥ 40)<br>• Immunotherapy unless on a stable maintenance schedule and not administered ≤ 24 h of a study visit<br>• Night-shift workers who did not adhere to a standard day/night awake/asleep cycle	MF 200 µg OD (20)	0	0
			Placebo (10)	0	0
Given (2010)	• Patients ≥12 yr with PAR<br>• Patients who must have been symptomatic to the perennial allergen and willing to maintain the same	• Severe physical obstruction of the nose (e.g., deviated septum or nasal polyp), recent nasal septal surgery or perforation, asthma unless it was mild intermittent asthma, rhinitis	FF 110 µg OD (160)	7 (4%)	• 1 (<1%) – AEs<br>• 5 (3) – Protocol deviation<br>• 1 (<1%) - Withdrew consent

Study	Inclusion criteria	Exclusion criteria	Name of INCS and no. of patients	No. of withdrawal (n, %)	Reasons for withdrawal (n)
	environment throughout the study	medicamentosa, acute or significant chronic sinusitis, ocular or upper respiratory bacterial or viral infection, chronic use of medications that could affect AR or its symptoms, and current tobacco use<br>• Using the following medications within the following periods before the screening: subcutaneous omalizumab within 5 months; corticosteroids (intranasal form within 4 weeks and inhaled, intramuscular, intravenous, and/or topical forms, except ≤ 1% hydrocortisone cream/ointment or equivalent, within 8 weeks); long-acting antihistamines within 10 days (except astemizole, 12 weeks); intranasal antihistamines or intranasal or ocular cromolyn within 14 days; short-acting prescription or over-the-counter antihistamines, oral or intranasal decongestants, anticholinergics, long-acting β-agonists, or oral antileukotrienes within 3 days; and H_2-receptor antagonists within 1 day	Placebo (155)	7 (5%)	• 1 (<1%) - AEs<br>• 3 (2%) - protocol deviation<br>• 1 (<1%) - lost to follow-up<br>• 2 (1%) - withdrew consent
Baena-Cagnani (2010)	• Subjects 3-11 yr (8–11 yr in Chile and Sweden) with ≥1-year history of PAR requiring over-the-counter or prescription treatment within the preceding year<br>• Patients were required to have a nasal congestion score ≥ 2 (indicating a symptom of at least moderate intensity), a total	• Patients with significant diseases other than AR, that could interfere with study evaluations<br>• Patients with asthma requiring chronic use of inhaled or systemic corticosteroids, or a history of or current clinically significant sinus infection, chronic purulent postnasal drip, rhinitis medicamentosa, allergy to two or more classes of drµgs, allergy	MF 100 µg OD (190)	8 (4%)	• 2 (1%) - AEs<br>• 1 (0.5%) - treatment failure<br>• 2 (1%) - lost to follow-up<br>• 3 (1.6%) - refused to remain in the study
			Placebo (191)	7 (4%)	• 3 (1.6%) - AEs<br>• 1 (0.5%) - treatment failure

Study	Inclusion criteria	Exclusion criteria	Name of INCS and no. of patients	No. of withdrawal (n, %)	Reasons for withdrawal (n)
	nasal symptom score (TNSS) ≥ 5, an investigator-assessed overall PAR score ≥ 2 at baseline, and at least moderate rhinorrhea and/or congestion documented in a subject treatment diary for ≥ 4 of the 7 days prior to baseline	to corticosteroids, or posterior subcapsular cataracts.<br>• Patients with nasal structural abnormalities, including large nasal polyps or marked septal deviation, that significantly interfered with nasal airflow<br>• Patients treated with inhaled corticosteroids for asthma for ≥ 2 months within the 12 months prior to enrollment or within 1 month before enrollment or two courses of systemic steroids, or any course lasting ≥ 14 days, within the 12 months preceding enrollment			• 1 (0.5%) - refused to remain in the study<br>• 2 (1%) – non-compliance
Weinstein (2009)	• Patients 2-5 yr with ≥1-year history of PAR and positive SPT or positive specific IgE to a relevant perennial allergen in the patient's environment (cat, dog, molds, dust mites, or others)<br>• Patients had to have a morning serum cortisol level of at least 138 nmol/L and a 30-minute poststimulation serum cortisol level of at least 496 nmol/L at screening	• Patients with asthma requiring long-term use of inhaled or systemic corticosteroids within 6 months of the study; URI or sinus infection requiring antibiotic therapy within 2 weeks of screening or viral URI within 7 days of screening; nasal polyps, deviated septum, or nasal/ facial anatomical abnormalities; a history of cataract or glaucoma; or hypersensitivity to glucocorticoids.	TAA 110 µg OD (236)	20 (8.5%)	• 6 (2.5%) – lost to follow up<br>• 6 (2.5%) – others<br>• 3 (1.3%) – wish to discontinue<br>• 4 (1.7%)– AEs<br>• 1 (0.4%) – lack of efficacy
			Placebo (238)	22 (9.2%)	• 7 (2.9%) – lost to follow up<br>• 3 (1.3%) – others<br>• 6 (2.5%) – wish to discontinue<br>• 3 (1.3%) – AEs<br>• 3 (1.3%) – lack of efficacy
Nathan (2008)	• Patients ≥12 yr in the USA and ≥18 yr in Canada with	• Patients with significant concomitant medical conditions; severe obstruction	FF 110 µg OD (149)	> 90% completed the study	NR

Study	Inclusion criteria	Exclusion criteria	Name of INCS and no. of patients	No. of withdrawal (n, %)	Reasons for withdrawal (n)
	PAR and positive SPT to appropriate perennial allergens within 12 months<br>• Patients who had a 2-year medical history and past treatment of PAR, including year-round symptoms, and were symptomatic for the appropriate perennial allergen and willing to maintain the same environment throughout the study<br>• Patients receiving immunotherapy could be enrolled when immunotherapy was not initiated within 30 days of the study entry or if the dose remained fixed during the 30 days before the study started and for the duration of the study	of the nose; nasal, ocular, or throat injury or surgery in the previous 3 months; asthma (except mild, intermittent asthma [USA] as defined by the National Asthma Education and Prevention Program or very mild asthma, such as that treated with short-acting β-agonists as needed [Canada] as defined by the Adult Asthma Consensus Guidelines Update 2003; rhinitis medicamentosa; infection within 2 weeks of visit 1 or during the screening period; or documented acute or significant chronic sinusitis<br>• Patients with SAR to a spring seasonal allergen (e.g., grass pollen) and had used INCS within 4 weeks of visit 1, other forms of corticosteroid within 8 weeks of visit 1, or any other allergy medications within a prespecified time- frame before visit 1<br>• Patients using any other medications that might affect AR or any immunosuppressive medications in the 8 weeks<br>• Pregnant or lactating females, smokers, or individuals with clinically significant abnormal electrocardiogram or laboratory test abnormality	Placebo (153)	> 90% completed the study	NR
Vasar (2008)	• Patients ≥12 yr with a ≥2 history of PAR and a positive SPT to appropriate perennial allergens within the previous 12 months<br>• Symptomatic patients at baseline and free of any	• Patients with significant concomitant medical conditions, a severe physical obstruction of the nose, rhinitis medicamentosa, sinusitis, glaucoma, cataracts, or ocular herpes simplex, or asthma (except for mild intermittent asthma).	FF 110 µg OD (151)	30 (20%)	NR
			Placebo (151)	32 (21%)	NR

Study	Inclusion criteria	Exclusion criteria	Name of INCS and no. of patients	No. of withdrawal (n, %)	Reasons for withdrawal (n)
	clinically relevant disease that could interfere with study evaluations<br>• Patients had to be willing to maintain the same environment (e.g., unchanged exposure to the causative allergen) for the entire duration of the study	• Using concomitant medications that could interfere with study assessments, including corticosteroids and other allergy medications<br>• An appropriate washout period was required for patients who had taken INS within the 4 weeks and all other corticosteroids within the 8 weeks before visit 1, except for topical hydrocortisone cream or ointment (1% or less, or equivalent); short-acting prescription and over-the-counter antihistamines within the 7 days; or long-acting antihistamines within the 10 days before visit 1			
Meltzer (2007)	• Patients ≥12 yr with ≥2-yr history of PAR and a positive SPT to ≥1 perennial allergens<br>• PAR required continuous or intermittent treatment in the past, with treatment expected to be required throughout the study<br>• Immunotherapy was permitted if the maintenance regimen remained unchanged for 30 days or more before the first study visit	• Patients with abnormal findings, including but not limited to nasal polyps and nasal tract malformations, rhinitis medicamentosa (within 60 days of screening), evidence of a respiratory tract infection or a significant medical disorder other than AR within 14 days before the screening visit, or a positive test result for hepatitis B, hepatitis C, or human immunodeficiency virus<br>• Patients with active asthma who required treatment with inhaled or systemic corticosteroids or routine use of β-agonists and patients who received prohibited medications during various washout periods	CIC 200 μg OD (238)	32 (13.4%)	• 11 (4.6%) - AEs
			Placebo (233)	30 (12.9%)	• 11 (4.7%) - AEs
Chervinsky (2007)	• Patients ≥12 yr with a ≥2- yr history of PAR and require treatment for the duration of the study and with a positive SPT to ≥1 perennial allergen	• Patients with any history or physical findings of nasal pathology, including nasal polyps or other clinically significant respiratory tract malformations, recent nasal biopsy,	CIC 200 μg OD (441)	123 (27.9%)	• lack of compliance
			Placebo (222)	66 (29.7%)	• lack of compliance

Study	Inclusion criteria	Exclusion criteria	Name of INCS and no. of patients	No. of withdrawal (n, %)	Reasons for withdrawal (n)
		nasal trauma, nasal surgery, atrophic rhinitis, or rhinitis medicamentosa.<br>• Patients with active asthma that required treatment with inhaled or systemic corticosteroids and/or routine use of β-agonists<br>• Patients with hypersensitivity to corticosteroids<br>• Patients with respiratory infection within 14 days or development of respiratory infection during baseline or use of antibiotics for acute conditions within 14 days of the screening visit<br>• Patients with cataracts or glaucoma			
Rosenblut (2007)	• Patients ≥12 yr with a ≥ 2-year history and past treatment of PAR and a positive SPT to a perennial allergen within 12 months	• Patients with any medical condition that might interfere with safety evaluations, including severe nasal obstruction; recent nasal septal or facial surgery; asthma (except mild intermittent asthma); rhinitis medicamentosa; recent bacterial or viral infection of the upper respiratory tract; sinusitis; Candida infection of nose or oropharynx; glaucoma, cataracts or ocular herpes simplex; a history of adrenal insufficiency or abnormal electrocardiogram or clinical laboratory test.<br>• Patients received: INCS within 4 weeks; inhaled, oral, intramuscular, intravenous and/or topical corticosteroids within 6 months of screening.	FF 110 µg OD (605)	157 (26%)	• 38 (6.3%) - AEs<br>• 9 (1.5%) - Lost to follow-up<br>• 49 (8.1%) - Protocol violation<br>• 48 (7.9%) - Withdrew consent<br>• 6 (0.9%) - Lack of efficacy<br>• 7 (1.2%) - Other reasons
			Placebo (201)	57 (28.4%)	• 7 (3.5%) - AEs<br>• 4 (2%) - Lost to follow-up<br>• 20 (10%) - Protocol violation<br>• 15 (7.5%) - Withdrew consent<br>• 5 (2.5%) - Lack of efficacy<br>• 6 (3%) - Other reasons
Tai (2003)	• Patients 16-60 yr, with a history of moderate to		FP 200 µg OD (14)	0	0

Study	Inclusion criteria	Exclusion criteria	Name of INCS and no. of patients	No. of withdrawal (n, %)	Reasons for withdrawal (n)
	severe PAR for at least the previous 6 months<br>• Patients who have an allergen-specific IgE examination, which was verified by MAST CLA (multiple-antigen simultaneous test chemiluminescent assay; Immuno Systems Inc, Mountain View, CA) at entry	• Patients who had received intranasal sodium cromolyn or nedocromil sodium within 6 weeks<br>• Patients who had received immunotherapy during the previous 12 months<br>• Patients who had had nasal surgery during the past 6 weeks<br>• Patients who had obstructing nasal polyps or significant deviation of the nasal septum<br>• Patients who had an infection of the sinuses or upper or lower respiratory tract in the previous 3 weeks	BUD 200 µg BID (10)	0	0
Fokkens (2002)	• Moderate to severe symptoms consistent with PAR for at least 1 year<br>• Need for treatment of nasal symptoms<br>• Moderate-to-severe symptom score (≥2) for blocked nose and at least mild (symptom score 0–1) severity of either runny nose or sneezing on 4 of 7 days of the run-in period	• Pollen allergy in season<br>• URI within 2 weeks before screening<br>• Rhinitis medicamentosa or structural abnormalities symptomatic enough to cause significant nasal obstruction<br>• Unstable asthma, i.e., requiring systemic or inhaled corticosteroids at doses higher than 800 µg/day<br>• Immunotherapy not on a constant maintenance dose<br>• Any other significant diseases<br>• Systemic corticosteroid therapy within 2 months<br>• Extensive application of topical cutaneous (group III) steroids, INCS, within 1 month before screening<br>• Other medication possibly interfering: antihistamines within 3 days, cromoglycate within 2 weeks, astemizole within 1 month before screening	BUD 128 µg OD (100)	NR	NR
			Placebo (102)	NR	NR

Study	Inclusion criteria	Exclusion criteria	Name of INCS and no. of patients	No. of withdrawal (n, %)	Reasons for withdrawal (n)
Kobayashi (1995)	• Patients 11-59 yr with ≥2-yr histories of PAR<br>• Having positive SPT to perennial allergens, eosinophils in nasal secretions at screening, and an aggregate nasal symptom (stuffiness, discharge, sneezing, and itching) score of 24 points (out of 48) on a four-point scale where 0 = absent to 3 = severe over the 4 days before the baseline visit	• Concomitant medications for rhinitis that might interfere with the interpretation of study results<br>• Patients who had used systemic corticosteroids within 42 days, nasal cromolyn sodium within 28 days, nasal or inhaled corticosteroids within 30 days, astemizole within 60 days, any investigational drµg within 90 days, or who had started immunotherapy within 6 months of screening<br>• Patients who had used any other medication for alleviation of PAR symptoms within 6 days of randomization	TAA 220 µg OD (88)	3 (3.4%)	• 1 (0.6%) - protocol deviations<br>• 1 (0.6%) - insufficient efficacy<br>• 3 (1.7%) - consent withdrawn<br>• 1 (0.6%) - other reasons
			Placebo (90)	3 (3.3%)	

BDP, beclomethasone dipropionate; BUD, budesonide; CIC, ciclesonide; FF, fluticasone furoate; FP, fluticasone propionate; MF, mometasone furoate; TAA, triamcinolone acetonide.

AR, allergic rhinitis; AEs, adverse events; BID, twice daily; INCS, intranasal corticosteroid; NR, not reported; OD, once daily; PAR, perennial allergic rhinitis; SAR seasonal allergic rhinitis; SPT, skin prick test; (i or r)TNSS, instantaneous or reflective total nasal symptom score; (i or r)TOSS, instantaneous or reflective total ocular symptom score; URI, upper respiratory tract infection; yr, year(s)

Supplementary Table S4. Interested outcomes of included studies

Study	Interested Outcomes	Points of Measurement										Definition of Interested outcomes
		Baseline	1 wk	2 wk	3 wk	4 wk	5 wk	6 wk	7 wk	8 wk		
Ratner (2015)	rTOSS	√	√	√								Each rTOSS assessment is a sum of 3 ocular symptoms for eye itching or burning, tearing or watering, and redness, with each symptom, scored on a scale of 0 (absent symptoms) to 3 (severe symptoms).
	Nasal congestion symptom score	√	√	√								NR
Igarashi (2012)	TOSS	√	√	√	√	√						The TOSS is the combination of ocular symptoms (eye itching, tearing), using a scale of 0 to 4 (0 = none, 1 = mild, 2 = moderate, 3 = severe, 4 = most severe), whereas the ophthalmologist's evaluation of clinical findings was scored on a scale of 0 to 3 (0 = none, 1 = mild, 2 = moderate, 3 = severe).
Meltzer (2011)	TNSS	√	√	√								Total nasal symptom score (TNSS): nasal congestion/stuffiness, rhinorrhea (nasal discharge/runny nose/postnasal drip), sneezing, and nasal itching. Subjects rated their symptoms on a 4-point scale: 0 = none, no evidence of symptoms; 1 = mild, 2 = moderate, and 3 = severe.
	Individual nasal symptoms	√	√	√								Nasal congestion/stuffiness, rhinorrhea (nasal discharge/runny nose/postnasal drip), sneezing, and nasal itching. Subjects rated their symptoms on a 4-point scale: 0 = none, no evidence of symptoms; 1 = mild, 2 = moderate, and 3 = severe.
Prenner et al. (2010)	TNSS	√	√	√								Subjects rated the severity of their symptoms, including rhinorrhea, nasal congestion, nasal itching, and sneezing on

Study	Interested Outcomes	Points of Measurement										Definition of Interested outcomes
		Baseline	1 wk	2 wk	3 wk	4 wk	5 wk	6 wk	7 wk	8 wk		
												a 4-point scale (0, none; 1, mild; 2, moderate; and 3, severe).
	TOSS	√	√	√								Subjects rated the severity of their symptoms, including redness of eyes, itching/ burning eyes, and tearing/watering eyes, on a 4-point scale (0, none; 1, mild; 2, moderate; and 3, severe).
Okubo (2009)	3TNSS	√	√	√								3TNSS was defined as the sum (0–9) of three individual symptom scores for sneezing, rhinorrhea, and nasal congestion, where each symptom was scored on a scale of 0–3 in the allergy diary.
	4TNSS	√	√	√								4TNSS (sum of scores for sneezing, rhinorrhea, nasal congestion, and nasal itching)
	Individual nasal symptom scores	√	√	√								Sneezing, rhinorrhea, nasal congestion, and nasal itching, where each symptom was scored on a scale of 0–3 in the allergy diary
Jacobs (2009)	TNSS	√	√	√								TNSS is the sum of each nasal symptom (nasal congestion, nasal itching, sneezing, and rhinorrhea) on a 4-point scale (0 = none, 1 = mild, 2 = moderate, 3 = severe).
	TOSS	√	√	√								TOSS is the sum of each ocular symptom (itching/burning eyes, tearing/watering eyes, and eye redness) on a 4-point scale (0 = none, 1 = mild, 2 = moderate, 3 = severe).
Andrews (2009)	TNSS	√	√	√								TNSS is the sum of each nasal symptom (nasal congestion, nasal itching, sneezing, and rhinorrhea) on a 4-point scale (0 = none, 1 = mild, 2 = moderate, 3 = severe).
	TOSS	√	√	√								TOSS is the sum of each ocular symptom (itching/burning eyes, tearing/watering eyes, and eye redness) on a 4-point scale

Study	Interested Outcomes	Points of Measurement										Definition of Interested outcomes
		Baseline	1 wk	2 wk	3 wk	4 wk	5 wk	6 wk	7 wk	8 wk		
												(0 = none, 1 = mild, 2 = moderate, 3 = severe).
	QoL	√	√	√								Health-related quality of life was assessed with the Nocturnal Rhinoconjunctivitis Quality of Life Questionnaire (NRQLQ), a 16-item, self-administered, disease-specific instrument that patients completed at the beginning and the end of the treatment period. The NRQLQ assesses the nocturnal quality of life over a 1-week interval. Items are grouped into four domains: sleep problems, sleep time problems, symptoms of waking in the morning, and practical problems. Each question is scored from 0 to 6, with higher scores indicating greater impairment in quality of life. An overall quality-of-life score was calculated from the mean score of all items. Mean scores for each domain were also calculated.
Kaiser (2007)	TNSS	√	√	√								TNSS is the sum of each nasal symptom (nasal congestion, nasal itching, sneezing, and rhinorrhea) on a 4-point scale (0 = none, 1 = mild, 2 = moderate, 3 = severe).
	TOSS	√	√	√								TOSS is the sum of each ocular symptom (itching/burning eyes, tearing/watering eyes, and eye redness) on a 4-point scale (0 = none, 1 = mild, 2 = moderate, 3 = severe).
	QoL	√	√	√								Health-related quality of life (HRQOL) was assessed with the Rhinoconjunctivitis Quality-of-Life Questionnaire (RQLQ), a 28-item self-administered questionnaire that assesses HRQOL (activities, sleep, non- nose/ non-eye symptoms, practical problems, nasal symptoms, emotional

Study	Interested Outcomes	Points of Measurement										Definition of Interested outcomes
		Baseline	1 wk	2 wk	3 wk	4 wk	5 wk	6 wk	7 wk	8 wk		
												problems, and eye symptoms) over 1 week. Each question was scored on a scale of 0 to 6 (higher scores indicate greater HRQOL impairment). The RQLQ was completed before drμg administration and reassessed at the end of the treatment or early withdrawal.
Fokkens (2007)	TNSS	√	√	√								TNSS is the sum of each nasal symptom (nasal congestion, nasal itching, sneezing, and rhinorrhea) on a 4-point scale (0 = none, 1 = mild, 2 = moderate, 3 = severe).
	TOSS	√	√	√								TOSS is the sum of each ocular symptom (itching/burning eyes, tearing/watering eyes, and eye redness) on a 4-point scale (0 = none, 1 = mild, 2 = moderate, 3 = severe).
	QoL	√	√	√								Quality of life was assessed at baseline, and after 2 weeks of treatment using the Rhinoconjunctivitis Quality of Life Questionnaire (RQLQ), a validated, 28-item, self-administered questionnaire that assesses the quality of life over the preceding week. Each question is scored on an increasing impairment scale of 0–6, with higher scores indicating greater impairment of quality of life; a reduction in score of 0.5 or greater is considered a clinically important difference.
Ratner (2006)	rTNSS	√	√	√	√	√						The reflective TNSS measures nasal symptom severity over the past 12 hours and is calculated as the sum of 4 nasal symptoms (runny nose, itchy nose, sneezing, and nasal congestion), each rated on a scale of 0 (no signs/symptoms evident) to 3 (signs/symptoms that interfere with daily activities).

Study	Interested Outcomes	Points of Measurement										Definition of Interested outcomes
		Baseline	1 wk	2 wk	3 wk	4 wk	5 wk	6 wk	7 wk	8 wk		
	RQLQ	√	√	√	√	√						The RQLQ is a disease-specific, validated quality-of-life questionnaire developed to measure physical, emotional, and social problems common to adults and adolescents with allergies.
Meltzer (2004)	TNSS											Total nasal symptom scores (the sum of discharge, stuffiness, itching, and sneezing scores; each symptom rated from 0 = absent to 3 = severe) recorded the morning of the randomization visit, plus scores from 3 of the 4 previous days, were required to equal at least 42 (out of a possible 84) points for patients to continue in the study.
Gawchik (2003)	TNSS	√	√	√								TNSS is the sum of each nasal symptom (nasal congestion, nasal itching, sneezing, and rhinorrhea) on a 4-point scale (0 = none, 1 = mild, 2 = moderate, 3 = severe).
Lumry (2003)	Individual nasal symptom scores	√	√	√	√							Nasal discharge, nasal stuffiness, nasal itching, and sneezing, each symptom was rated on a four-point scale, where 0 = symptom absent; 1 = mild, 2 = moderate, and 3 = severe.
	Nasal index score	√	√	√	√							A nasal index score was also calculated for the combined symptoms of nasal discharge, nasal stuffiness, and sneezing (resulting in a possible total score of 0–9).
	Health-related quality of life	√	√	√	√							The patient was asked to assess the effect of nose/ eye symptoms on the following factors during the preceding week: sleep, non-hay fever symptoms, practical problems, nasal symptoms, eye symptoms, activities, and emotional impact. The severity of each nose/eye symptom for sleep, non-hay fever symptoms, practical problems, nasal symptoms, eye symptoms, and activities were scored on a seven-point

Study	Interested Outcomes	Points of Measurement										Definition of Interested outcomes
		Baseline	1 wk	2 wk	3 wk	4 wk	5 wk	6 wk	7 wk	8 wk		
												scale, where 0 = not troubled; 1 = hardly troubled at all; 2 = somewhat troubled; 3 = moderately troubled; 4 = quite a bit troubled; 5 = very troubled; and 6 = extremely troubled. The severity of each nose/eye symptom in terms of its emotional impact was also scored on a seven-point scale, assessing whether the impact was 0 = none of the time; 1 = hardly any time at all; 2 = a small part of the time; 3 = some of the time; 4 = a good part of the time; 5 = most of the time; and 6 = all of the time.
Berger (2003)	Individual nasal symptom scores	√	√	√	√							Symptoms included nasal discharge (anterior and/or posterior drainage), nasal stuffiness, nasal itching, and sneezing. Individual symptom severity was assessed on a scale of 0 to 3 (0 = absent; 1 = mild, 2 = moderate, 3 = severe.
	TNSS	√	√	√	√							TNSS is the sum of each nasal symptom (nasal congestion, nasal itching, sneezing, and rhinorrhea) on a 4-point scale (0 = none, 1 = mild, 2 = moderate, 3 = severe).
	RQLQ	√	√	√	√							The RQLQ comprised 28 items in 7 dimensions: activities, emotions, eye symptoms, nasal symptoms, non-hay fever, practical problems, and sleep. Mean dimension scores and overall HRQL scores were calculated using the RQLQ 7-point Likert scale (0 to 6; lower scores reflect the better HRQL).
Gross (2002)	Individual nasal symptoms	√	√	√	√							Individual symptom severity was evaluated on a scale ranging from 0 to 3 (0 = absent; 1 = mild, present but not annoying; 2 = moderate, present and annoying, but does not interfere with sleep or daily living; 3 = severe, interferes

Study	Interested Outcomes	Points of Measurement										Definition of Interested outcomes
		Baseline	1 wk	2 wk	3 wk	4 wk	5 wk	6 wk	7 wk	8 wk		
												with/or unable to carry out activities of daily living or sleep).
	TNSS	√	√	√	√							TNSS is the sum of each nasal symptom (nasal congestion, nasal itching, sneezing, and rhinorrhea) on a 4-point scale (0 = none, 1 = mild, 2 = moderate, 3 = severe).
	RQLQ	√	√	√	√							The RQLQ was used at visits 2 and 3 to evaluate the disease-specific quality of life in all patients according to seven dimensions: activities, emotions, eye symptoms, nasal symptoms, non-hay fever problems, practical problems, and sleep.
Meltzer (1998)	Individual nasal symptoms	√	√	√								Nasal symptoms (congestion, rhinorrhea, itching, and sneezing) were scored on a scale (0 = none, 1 = mild, 2 = moderate, and 3 = severe.
	TNSS	√	√	√								TNSS is the sum of each nasal symptom (nasal congestion, nasal itching, sneezing, and rhinorrhea) on a 4-point scale (0 = none, 1 = mild, 2 = moderate, 3 = severe).
Bronsky (1996)	Individual nasal symptoms	√	√	√	√	√						Individual nasal symptoms (sneezing, nasal obstruction, rhinorrhea. and nasal itching) on a visual analog scale ranging from 0 (absent) to 100 (severe).
	TNSS	√	√	√	√	√						Combination of individual nasal symptoms (sneezing, nasal obstruction, rhinorrhea. and nasal itching)
van Bavel (1994)	Individual nasal symptoms	√	√	√								Individual nasal symptoms (sneezing, nasal obstruction, rhinorrhea, and nasal itching) on a visual analog scale ranging from 0 (absent) to 100 (severe)
	TNSS	√	√	√								The total nasal symptom score (derived by adding individual symptom scores for sneezing, nasal obstruction, rhinorrhea, and nasal itching)

Study	Interested Outcomes	Points of Measurement										Definition of Interested outcomes
		Baseline	1 wk	2 wk	3 wk	4 wk	5 wk	6 wk	7 wk	8 wk		
Ratner (1992)	Total nasal symptoms by VAS	√	√	√	√							The total scores of four individual nasal symptoms by VAS
	Individual nasal symptoms by VAS	√	√	√	√							The severity of individual nasal symptoms was scored by clinicians at each visit and by patients on daily diary cards at the end of each day on visual analog scales from 0 (no symptoms) to 100 (severe symptoms).
Karaulov (2019)	rTNSS	√	√	√	√	√						The rTNSS is a validated symptom-scoring system that consists of the sum of four individual participant-assessed symptom scores for rhinorrhea, nasal congestion, nasal itching, and sneezing. Each symptom was evaluated over the preceding 24 h, according to the 5-point scale: 0 = none (no sign/symptom present), 1 = mild (symptom present but not annoying or troublesome; easily tolerated), 2 = moderate (symptom frequently troublesome but not interfering with normal daily activities or sleep; tolerable), 3 = severe (symptom sufficiently troublesome to interfere with normal daily activities or sleep; hard to tolerate), or 4 = very severe (symptom severe enoμgh to warrant an immediate visit to the physician).
	QoL	√	√	√	√	√						QoL was measured by the mini Rhinoconjunctivitis Quality of Life Questionnaire (miniRQLQ) from baseline to the last day of treatment, and patient and physician satisfaction with therapy (measured by a 5-point scale questionnaire) after 28 days of treatment.
Meltzer (2010)	Individual nasal symptoms	√	√	√	√	√						Patients rated nasal symptoms (obstruction/blockage/congestion, drainage [anterior/posterior], nasal itch, and

Study	Interested Outcomes	Points of Measurement										Definition of Interested outcomes
		Baseline	1 wk	2 wk	3 wk	4 wk	5 wk	6 wk	7 wk	8 wk		
												sneezing) on a scale from 0 (none) to 6 (very severe).
	TNSS	√	√	√	√	√						The TNSS equaled the summed individual nasal symptom scores.
	PNIF	√	√	√	√	√						Peak nasal inspiratory flow (PNIF) measurements were made before morning dosing and repeated approximately 12 hours later using an In-Check portable nasal flow meter (Clement Clarke International Ltd, Harlow, England).
Given (2010)	TNSS	√	√	√	√	√						Patients used an electronic diary to rate symptoms of nasal congestion, rhinorrhea, nasal itching, and sneezing on a scale ranging from 0 (symptom not present) to 3 (symptom difficult to tolerate; interferes with activities of daily living and/or sleeping).
	TOSS	√	√	√	√	√						Patients used an electronic diary to rate ocular symptoms of itching/ burning, tearing/watering, and redness on a scale ranging from 0 (symptom not present) to 3 (symptom difficult to tolerate; interferes with activities of daily living and/or sleeping).
	QoL	√	√	√	√	√						Health-related quality of life was assessed with the RQLQ(S), a 28-item, self-administered, disease-specific instrument that patients completed at the beginning and the end of the treatment period or on early withdrawal from the study. The RQLQ(S) assesses the quality of life over a 1-week interval. Items are grouped into seven domains: activity limitations, sleep problems, non-nose/non-eye symptoms,

Study	Interested Outcomes	Points of Measurement										Definition of Interested outcomes
		Baseline	1 wk	2 wk	3 wk	4 wk	5 wk	6 wk	7 wk	8 wk		
												practical problems, nasal symptoms, eye symptoms, and emotional function. Each question is scored from 0 to 6, with higher scores indicating greater impairment in quality of life.
Baena-Cagnani (2010)	TNSS	√	√	√	√	√						Grading of symptom severity for TNSS is recorded as 0 = none, 1= mild, 2= moderate, 3= severe).
Weinstein (2009)	TNSS	√	√	√	√	√						The TNSS is the sum of the scores for nasal stuffiness, nasal discharge, sneezing, and itching. Symptoms were recorded by caregivers in daily diaries on arising in the morning immediately before the administration of study medication. Symptom severity was rated on a 4-point scale (0 = symptom absent and 3 = severe).
Nathan (2008)	TNSS	√	√	√	√	√						Patients completed a diary card on which they rated the severity of nasal symptoms (rhinorrhea, nasal congestion, itching, and sneezing) and non-nasal symptoms (ocular symptoms of itching or burning, tearing or watering, and redness, and throat or palatal itching) of AR in a reflective and instantaneous manner. Symptoms were rated on a 4-point categorical scale: 0 indicating none (no sign or symptom evident); 1, mild (sign or symptom clearly present but causing minimal awareness and easily tolerated); 2, moderate (definite awareness of sign or symptom that is bothersome but tolerable); and 3, severe (sign or symptom that is hard to tolerate and causes interference with activities of daily living and/or sleeping).
	QoL	√	√	√	√	√						This is a 28-item, self-administered, disease-specific quality-of-life (QOL) instrument that assesses QOL during 1

Study	Interested Outcomes	Points of Measurement										Definition of Interested outcomes
		Baseline	1 wk	2 wk	3 wk	4 wk	5 wk	6 wk	7 wk	8 wk		
												week. Each question is scored from 0 to 6, with higher scores indicating greater impairment of QOL. Items are grouped into 7 domains: activity limitations, sleep problems, non-nasal and non-ocular symptoms, practical problems, nasal symptoms, ocular symptoms, and emotional function. An overall QOL score is calculated from the mean score of all items.
Vasar (2008)	TNSS	√	√	√	√	√	√	√				Individual nasal symptom scores for nasal congestion, rhinorrhea, sneezing, and nasal itching were recorded in an electronic diary (eDiary) and combined to give the total nasal symptom score (TNSS).
	TOSS	√	√	√	√	√	√	√				Individual ocular symptom scores for itching/burning eyes, tearing/watering eyes, and eye redness also was recorded in the eDiary and combined to give the total ocular symptom score (TOSS).
	PNIF	√	√	√	√	√	√	√				Patients measured their PNIF rate twice daily (A.M. predose and P.M. postdose) using a handheld nasal inspiratory flow meter and face mask and recorded measurements in the eDiary.
	QoL	√	√	√	√	√	√	√				Rhinitis-related quality of life was assessed using the Rhinoconjunctivitis Quality of Life Questionnaire (RQLQ). This 28-item, self-administered, disease-specific quality-of-life instrument assesses the impact of allergic rhinitis on quality of life using a 1-week recall period. Items are grouped into seven domains: daily activities, sleep, non–hay fever symptoms, practical problems, nasal symptoms, ocular symptoms, and emotional impact, and are assessed using a seven-point Likert scale

Study	Interested Outcomes	Points of Measurement										Definition of Interested outcomes
		Baseline	1 wk	2 wk	3 wk	4 wk	5 wk	6 wk	7 wk	8 wk		
												(where 0 = no impairment and 6 = maximum impairment). The questionnaire assesses an overall impact on quality of life and impact on each of the seven individual domains, with an improvement of ≥ 0.5 over placebo representing the minimally important difference.
Meltzer (2007)	rTNSS	√	√	√	√	√	√	√				Reflective TNSS was the sum of the scores for 4 nasal symptoms (runny nose, itchy nose, sneezing, and nasal congestion). Nasal symptoms were evaluated twice daily on a severity scale ranging from 0 (no signs or symptoms) to 3 (severe signs or symptoms).
	RQLQ	√			√			√				The combined adult and adolescent Rhinoconjunctivitis Quality of Life Questionnaire (RQLQ) score
Chervinsky (2007)	rTNSS	√	√	√	√	√	√	√	√	√	√	Reflective TNSS measures nasal symptom severity over the previous 24 hours and is calculated as the sum of the 4 individual nasal symptoms scores, each rated on a scale of 0 (no signs or symptoms evident) to 3 (signs or symptoms that interfere with daily activities).
	RQLQ	√	√	√	√	√	√	√	√	√	√	RQLQ is a disease-specific, validated QOL questionnaire developed for the measurement of physical, emotional, and social problems common to adults and adolescents with allergies, rated on a 7-point scale (with 0 indicating least severe and 6 indicating extremely severe) for questions related to activities, sleep, non-nose or non-eye symptoms, practical problems, nasal symptoms, eye symptoms, and emotional factors.

Study	Interested Outcomes	Points of Measurement										Definition of Interested outcomes
		Baseline	1 wk	2 wk	3 wk	4 wk	5 wk	6 wk	7 wk	8 wk		
Rosenblut (2007)	TNSS	√	√	√	√	√	√	√	√	√	52 wk	Reflective total nasal symptom score (rTNSS) is the sum of individual scores for rhinorrhoea, nasal congestion, nasal itching, and sneezing, each rated on a 4-point categorical scale, 0 = no symptoms, 3 = severe symptoms).
Tai (2003)	TNSS	√	√	√	√	√	√	√	√	√		The total nasal symptom score is the sum of 6 individual symptom scores (nasal blockage (stuffiness) on waking, Nasal blockage (stuffiness) rest of day, sneezing, nasal itching/rubbing, rhinorrhea (runny nose), eye itching). The daily total score ranged from 0 (best) to 18 (worst).
Fokkens (2002)	Individual nasal symptom scores	√	√	√	√	√	√	√				Symptom scores included blocked nose, runny nose, and sneezing. Severity ranges from 0 to 3: no, 0; mild, 1; moderate, 2; and severe, 3.
	Combined nasal symptom score	√	√	√	√	√	√	√				The combined nasal symptom score was calculated as the sum of the individual nasal symptom scores.
	PNIF	√	√	√	√	√	√	√				PNIF was measured using a Youlten flow meter (Clement Clarke International, Harlow, UK).
	QoL	√						√				For the younger children (aged 6 to 11 years), an interviewer-administered questionnaire, the Pediatric Rhinoconjunctivitis Quality of Life Questionnaire, was used by the study nurse or investigator.<br>For children above 12 years of age, a self-administered questionnaire, the Adolescent Rhinoconjunctivitis Quality of Life Questionnaire, was used.
Kobayashi (1995)	TNSS	√	√	√	√	√						The severity of nasal stuffiness, nasal discharge, sneezing, and nasal itching was rated once a day before bedtime and

Study	Interested Outcomes	Points of Measurement										Definition of Interested outcomes
		Baseline	1 wk	2 wk	3 wk	4 wk	5 wk	6 wk	7 wk	8 wk		
												reflected the patient's condition during the previous 24 hours on a scale of 0 to 3(0 = absent, 1= mild, 2 = moderate, and 3 = severe).

PNIF, peak nasal inspiratory flow; QoL, quality of life; RQLQ, Rhinoconjunctivitis Quality of Life Questionnaire; (i or r)TNSS, instantaneous or reflective total nasal symptom score; (i or r)TOSS, instantaneous or reflective total ocular symptom score; VAS, visual analog scale; wk, week(s)

Supplementary Table S5. Cochrane Risk of Bias 2 (RoB-2)

**Unique ID**	1	**Study ID**	722	**Assessor**	KS TK
**Ref or Label**	Ratner_2015	**Aim**	assignment to intervention (the 'intention-to-treat' effect)		
**Experimental**	FP	**Comparator**	Placebo	**Source**	Journal article(s)
**Outcome**		**Results**		**Weight**	

**Domain**	**Signalling question**	**Response**	**Comments**
**Bias arising from the randomization process**	1.1 Was the allocation sequence random?	PY	
	1.2 Was the allocation sequence concealed until participants were enrolled and assigned to interventions?	PY	
	1.3 Did baseline differences between intervention groups sμggest a problem with the randomization process?	N	
	**Risk of bias judgement**	**Low**	
**Bias due to deviations from intended interventions**	2.1.Were participants aware of their assigned intervention during the trial?	PN	
	2.2.Were carers and people delivering the interventions aware of participants' assigned intervention during the trial?	PN	
	2.3. If Y/PY/NI to 2.1 or 2.2: Were there deviations from the intended intervention that arose because of the experimental context?	NA	
	2.4 If Y/PY to 2.3: Were these deviations likely to have affected the outcome?	NA	
	2.5. If Y/PY/NI to 2.4: Were these deviations from intended intervention balanced between groups?	NA	

	2.6 Was an appropriate analysis used to estimate the effect of assignment to intervention?	Y	
	2.7 If N/PN/NI to 2.6: Was there potential for a substantial impact (on the result) of the failure to analyse participants in the group to which they were randomized?	NA	
	**Risk of bias judgement**	**Low**	
**Bias due to missing outcome data**	3.1 Were data for this outcome available for all, or nearly all, participants randomized?	Y	
	3.2 If N/PN/NI to 3.1: Is there evidence that result was not biased by missing outcome data?	NA	
	3.3 If N/PN to 3.2: Could missingness in the outcome depend on its true value?	NA	
	3.4 If Y/PY/NI to 3.3: Is it likely that missingness in the outcome depended on its true value?	NA	
	**Risk of bias judgement**	**Low**	
**Bias in measurement of the outcome**	4.1 Was the method of measuring the outcome inappropriate?	N	
	4.2 Could measurement or ascertainment of the outcome have differed between intervention groups?	N	
	4.3 Were outcome assessors aware of the intervention received by study participants?	N	
	4.4 If Y/PY/NI to 4.3: Could assessment of the outcome have been influenced by knowledge of intervention received?	NA	
	4.5 If Y/PY/NI to 4.4: Is it likely that assessment of the outcome was influenced by knowledge of intervention received?	NA	
	**Risk of bias judgement**	**Low**	
**Bias in selection of the reported result**	5.1 Were the data that produced this result analysed in accordance with a pre-specified analysis plan that was finalized before unblinded outcome data were available for analysis?	Y	

	5.2 ... multiple eligible outcome measurements (e.g. scales, definitions, time points) within the outcome domain?			N	
	5.3 ... multiple eligible analyses of the data?			N	
	**Risk of bias judgement**			**Low**	
**Overall bias**	**Risk of bias judgement**			**Low**	
**Unique ID**	2	**Study ID**	9991	**Assessor**	KS TK
**Ref or Label**	Igarashi_2012	**Aim**	assignment to intervention (the 'intention-to-treat' effect)		
**Experimental**	MF	**Comparator**	Placebo	**Source**	Journal article(s)
**Outcome**		**Results**		**Weight**	
**Domain**	**Signalling question**			**Response**	**Comments**
**Bias arising from the randomization process**	1.1 Was the allocation sequence random?			Y	
	1.2 Was the allocation sequence concealed until participants were enrolled and assigned to interventions?			Y	
	1.3 Did baseline differences between intervention groups sμggest a problem with the randomization process?			N	
	**Risk of bias judgement**			**Low**	
	2.1.Were participants aware of their assigned intervention during the trial?			PN	

**Bias due to deviations from intended interventions**	2.2.Were carers and people delivering the interventions aware of participants' assigned intervention during the trial?	PN	
	2.3. If Y/PY/NI to 2.1 or 2.2: Were there deviations from the intended intervention that arose because of the experimental context?	NA	
	2.4 If Y/PY to 2.3: Were these deviations likely to have affected the outcome?	NA	
	2.5. If Y/PY/NI to 2.4: Were these deviations from intended intervention balanced between groups?	NA	
	2.6 Was an appropriate analysis used to estimate the effect of assignment to intervention?	PN	
	2.7 If N/PN/NI to 2.6: Was there potential for a substantial impact (on the result) of the failure to analyse participants in the group to which they were randomized?	PN	
	**Risk of bias judgement**	**Some concerns**	
**Bias due to missing outcome data**	3.1 Were data for this outcome available for all, or nearly all, participants randomized?	N	
	3.2 If N/PN/NI to 3.1: Is there evidence that result was not biased by missing outcome data?	PN	
	3.3 If N/PN to 3.2: Could missingness in the outcome depend on its true value?	PY	
	3.4 If Y/PY/NI to 3.3: Is it likely that missingness in the outcome depended on its true value?	PY	
	**Risk of bias judgement**	**High**	
**Bias in measurement of the outcome**	4.1 Was the method of measuring the outcome inappropriate?	N	
	4.2 Could measurement or ascertainment of the outcome have differed between intervention groups?	N	
	4.3 Were outcome assessors aware of the intervention received by study participants?	N	

	4.4 If Y/PY/NI to 4.3: Could assessment of the outcome have been influenced by knowledge of intervention received?			NA	
	4.5 If Y/PY/NI to 4.4: Is it likely that assessment of the outcome was influenced by knowledge of intervention received?			NA	
	**Risk of bias judgement**			**Low**	
**Bias in selection of the reported result**	5.1 Were the data that produced this result analysed in accordance with a pre-specified analysis plan that was finalized before unblinded outcome data were available for analysis?			Y	
	5.2 ... multiple eligible outcome measurements (e.g. scales, definitions, time points) within the outcome domain?			PN	
	5.3 ... multiple eligible analyses of the data?			PN	
	**Risk of bias judgement**			**Low**	
**Overall bias**	**Risk of bias judgement**			**High**	Very small sample size
**Unique ID**	3	**Study ID**	1107	**Assessor**	KS TK
**Ref or Label**	Meltzer_2011	**Aim**	assignment to intervention (the 'intention-to-treat' effect)		
**Experimental**	MF	**Comparator**	Placebo	**Source**	Journal article(s)
**Outcome**		**Results**		**Weight**	
**Domain**	**Signalling question**			**Response**	**Comments**
**Bias arising from the randomization process**	1.1 Was the allocation sequence random?			Y	

	1.2 Was the allocation sequence concealed until participants were enrolled and assigned to interventions?	Y	
	1.3 Did baseline differences between intervention groups sμggest a problem with the randomization process?	N	
	**Risk of bias judgement**	**Low**	
**Bias due to deviations from intended interventions**	2.1.Were participants aware of their assigned intervention during the trial?	N	
	2.2.Were carers and people delivering the interventions aware of participants' assigned intervention during the trial?	N	
	2.3. If Y/PY/NI to 2.1 or 2.2: Were there deviations from the intended intervention that arose because of the experimental context?	NA	
	2.4 If Y/PY to 2.3: Were these deviations likely to have affected the outcome?	NA	
	2.5. If Y/PY/NI to 2.4: Were these deviations from intended intervention balanced between groups?	NA	
	2.6 Was an appropriate analysis used to estimate the effect of assignment to intervention?	Y	
	2.7 If N/PN/NI to 2.6: Was there potential for a substantial impact (on the result) of the failure to analyse participants in the group to which they were randomized?	NA	
	**Risk of bias judgement**	**Low**	
**Bias due to missing outcome data**	3.1 Were data for this outcome available for all, or nearly all, participants randomized?	Y	
	3.2 If N/PN/NI to 3.1: Is there evidence that result was not biased by missing outcome data?	NA	
	3.3 If N/PN to 3.2: Could missingness in the outcome depend on its true value?	NA	
	3.4 If Y/PY/NI to 3.3: Is it likely that missingness in the outcome depended on its true value?	NA	

	**Risk of bias judgement**			**Low**	
**Bias in measurement of the outcome**	4.1 Was the method of measuring the outcome inappropriate?			N	
	4.2 Could measurement or ascertainment of the outcome have differed between intervention groups?			N	
	4.3 Were outcome assessors aware of the intervention received by study participants?			N	
	4.4 If Y/PY/NI to 4.3: Could assessment of the outcome have been influenced by knowledge of intervention received?			NA	
	4.5 If Y/PY/NI to 4.4: Is it likely that assessment of the outcome was influenced by knowledge of intervention received?			NA	
	**Risk of bias judgement**			**Low**	
**Bias in selection of the reported result**	5.1 Were the data that produced this result analysed in accordance with a pre-specified analysis plan that was finalized before unblinded outcome data were available for analysis?			Y	
	5.2 ... multiple eligible outcome measurements (e.g. scales, definitions, time points) within the outcome domain?			N	
	5.3 ... multiple eligible analyses of the data?			N	
	**Risk of bias judgement**			**Low**	
**Overall bias**	**Risk of bias judgement**			**Low**	
**Unique ID**	4	**Study ID**	1117	**Assessor**	KS TK
**Ref or Label**	Prenner_2010	**Aim**	assignment to intervention (the 'intention-to-treat' effect)		

**Experimental**	MF	**Comparator**	Placebo	**Source**	Journal article(s)
**Outcome**		**Results**		**Weight**	

**Domain**	**Signalling question**	**Response**	**Comments**
**Bias arising from the randomization process**	1.1 Was the allocation sequence random?	Y	
	1.2 Was the allocation sequence concealed until participants were enrolled and assigned to interventions?	Y	
	1.3 Did baseline differences between intervention groups sµggest a problem with the randomization process?	N	
	**Risk of bias judgement**	**Low**	
**Bias due to deviations from intended interventions**	2.1.Were participants aware of their assigned intervention during the trial?	N	
	2.2.Were carers and people delivering the interventions aware of participants' assigned intervention during the trial?	N	
	2.3. If Y/PY/NI to 2.1 or 2.2: Were there deviations from the intended intervention that arose because of the experimental context?	NA	
	2.4 If Y/PY to 2.3: Were these deviations likely to have affected the outcome?	NA	
	2.5. If Y/PY/NI to 2.4: Were these deviations from intended intervention balanced between groups?	NA	
	2.6 Was an appropriate analysis used to estimate the effect of assignment to intervention?	Y	
	2.7 If N/PN/NI to 2.6: Was there potential for a substantial impact (on the result) of the failure to analyse participants in the group to which they were randomized?	NA	
	**Risk of bias judgement**	**Low**	

**Bias due to missing outcome data**	3.1 Were data for this outcome available for all, or nearly all, participants randomized?	Y	But Final number < calculated sample size?
	3.2 If N/PN/NI to 3.1: Is there evidence that result was not biased by missing outcome data?	NA	
	3.3 If N/PN to 3.2: Could missingness in the outcome depend on its true value?	NA	
	3.4 If Y/PY/NI to 3.3: Is it likely that missingness in the outcome depended on its true value?	NA	
	**Risk of bias judgement**	**Low**	
**Bias in measurement of the outcome**	4.1 Was the method of measuring the outcome inappropriate?	N	
	4.2 Could measurement or ascertainment of the outcome have differed between intervention groups?	N	
	4.3 Were outcome assessors aware of the intervention received by study participants?	N	
	4.4 If Y/PY/NI to 4.3: Could assessment of the outcome have been influenced by knowledge of intervention received?	NA	
	4.5 If Y/PY/NI to 4.4: Is it likely that assessment of the outcome was influenced by knowledge of intervention received?	NA	
	**Risk of bias judgement**	**Low**	
**Bias in selection of the reported result**	5.1 Were the data that produced this result analysed in accordance with a pre-specified analysis plan that was finalized before unblinded outcome data were available for analysis?	Y	
	5.2 ... multiple eligible outcome measurements (e.g. scales, definitions, time points) within the outcome domain?	N	
	5.3 ... multiple eligible analyses of the data?	N	
	**Risk of bias judgement**	**Low**	

**Overall bias**	**Risk of bias judgement**			**Low**	
**Unique ID**	5	**Study ID**	254	**Assessor**	KS TK
**Ref or Label**	Okubo_2009	**Aim**	assignment to intervention (the 'intention-to-treat' effect)		
**Experimental**	FF	**Comparator**	FP	**Source**	Journal article(s)
**Outcome**		**Results**		**Weight**	
**Domain**	**Signalling question**			**Response**	**Comments**
**Bias arising from the randomization process**	1.1 Was the allocation sequence random?			Y	
	1.2 Was the allocation sequence concealed until participants were enrolled and assigned to interventions?			Y	
	1.3 Did baseline differences between intervention groups sμggest a problem with the randomization process?			N	
	**Risk of bias judgement**			**Low**	
**Bias due to deviations from intended interventions**	2.1.Were participants aware of their assigned intervention during the trial?			PN	4 arms (2 active vs 2 matched packaging placebo) Patients wouldn't know that they received medication or placebo but the device between 2 active arms are unblinded
	2.2.Were carers and people delivering the interventions aware of participants' assigned intervention during the trial?			N	

	2.3. If Y/PY/NI to 2.1 or 2.2: Were there deviations from the intended intervention that arose because of the experimental context?	NA	
	2.4 If Y/PY to 2.3: Were these deviations likely to have affected the outcome?	NA	
	2.5. If Y/PY/NI to 2.4: Were these deviations from intended intervention balanced between groups?	NA	
	2.6 Was an appropriate analysis used to estimate the effect of assignment to intervention?	Y	
	2.7 If N/PN/NI to 2.6: Was there potential for a substantial impact (on the result) of the failure to analyse participants in the group to which they were randomized?	NA	
	**Risk of bias judgement**	**Low**	
**Bias due to missing outcome data**	3.1 Were data for this outcome available for all, or nearly all, participants randomized?	Y	
	3.2 If N/PN/NI to 3.1: Is there evidence that result was not biased by missing outcome data?	NA	
	3.3 If N/PN to 3.2: Could missingness in the outcome depend on its true value?	NA	
	3.4 If Y/PY/NI to 3.3: Is it likely that missingness in the outcome depended on its true value?	NA	
	**Risk of bias judgement**	**Low**	
**Bias in measurement of the outcome**	4.1 Was the method of measuring the outcome inappropriate?	N	
	4.2 Could measurement or ascertainment of the outcome have differed between intervention groups?	N	
	4.3 Were outcome assessors aware of the intervention received by study participants?	PN	
	4.4 If Y/PY/NI to 4.3: Could assessment of the outcome have been influenced by knowledge of intervention received?	NA	

	4.5 If Y/PY/NI to 4.4: Is it likely that assessment of the outcome was influenced by knowledge of intervention received?			NA	
	**Risk of bias judgement**			**Low**	
**Bias in selection of the reported result**	5.1 Were the data that produced this result analysed in accordance with a pre-specified analysis plan that was finalized before unblinded outcome data were available for analysis?			Y	
	5.2 ... multiple eligible outcome measurements (e.g. scales, definitions, time points) within the outcome domain?			N	
	5.3 ... multiple eligible analyses of the data?			N	
	**Risk of bias judgement**			**Low**	
**Overall bias**	**Risk of bias judgement**			**Low**	
**Unique ID**	6	**Study ID**	472	**Assessor**	KS TK
**Ref or Label**	Jacobs_2009	**Aim**	assignment to intervention (the 'intention-to-treat' effect)		
**Experimental**	FF	**Comparator**	Placebo	**Source**	Journal article(s)
**Outcome**		**Results**		**Weight**	
**Domain**	**Signalling question**			**Response**	**Comments**
**Bias arising from the randomization process**	1.1 Was the allocation sequence random?			Y	
	1.2 Was the allocation sequence concealed until participants were enrolled and assigned to interventions?			Y	

	1.3 Did baseline differences between intervention groups sμggest a problem with the randomization process?	N	
	**Risk of bias judgement**	**Low**	
**Bias due to deviations from intended interventions**	2.1.Were participants aware of their assigned intervention during the trial?	N	
	2.2.Were carers and people delivering the interventions aware of participants' assigned intervention during the trial?	N	
	2.3. If Y/PY/NI to 2.1 or 2.2: Were there deviations from the intended intervention that arose because of the experimental context?	NA	
	2.4 If Y/PY to 2.3: Were these deviations likely to have affected the outcome?	NA	
	2.5. If Y/PY/NI to 2.4: Were these deviations from intended intervention balanced between groups?	NA	
	2.6 Was an appropriate analysis used to estimate the effect of assignment to intervention?	Y	
	2.7 If N/PN/NI to 2.6: Was there potential for a substantial impact (on the result) of the failure to analyse participants in the group to which they were randomized?	NA	
	**Risk of bias judgement**	**Low**	
**Bias due to missing outcome data**	3.1 Were data for this outcome available for all, or nearly all, participants randomized?	Y	
	3.2 If N/PN/NI to 3.1: Is there evidence that result was not biased by missing outcome data?	NA	
	3.3 If N/PN to 3.2: Could missingness in the outcome depend on its true value?	NA	
	3.4 If Y/PY/NI to 3.3: Is it likely that missingness in the outcome depended on its true value?	NA	
	**Risk of bias judgement**	**Low**	

**Bias in measurement of the outcome**	4.1 Was the method of measuring the outcome inappropriate?			N	
	4.2 Could measurement or ascertainment of the outcome have differed between intervention groups?			N	
	4.3 Were outcome assessors aware of the intervention received by study participants?			N	
	4.4 If Y/PY/NI to 4.3: Could assessment of the outcome have been influenced by knowledge of intervention received?			NA	
	4.5 If Y/PY/NI to 4.4: Is it likely that assessment of the outcome was influenced by knowledge of intervention received?			NA	
	**Risk of bias judgement**			**Low**	
**Bias in selection of the reported result**	5.1 Were the data that produced this result analysed in accordance with a pre-specified analysis plan that was finalized before unblinded outcome data were available for analysis?			Y	
	5.2 ... multiple eligible outcome measurements (e.g. scales, definitions, time points) within the outcome domain?			N	
	5.3 ... multiple eligible analyses of the data?			N	
	**Risk of bias judgement**			**Low**	
**Overall bias**	**Risk of bias judgement**			**Low**	
**Unique ID**	7	**Study ID**	838	**Assessor**	KS TK
**Ref or Label**	Andrews_2009	**Aim**	assignment to intervention (the 'intention-to-treat' effect)		
**Experimental**	FF	**Comparator**	Fex/Placebo	**Source**	Journal article(s)

Outcome		Results		Weight	
**Domain**	**Signalling question**			**Response**	**Comments**
**Bias arising from the randomization process**	1.1 Was the allocation sequence random?			PY	
	1.2 Was the allocation sequence concealed until participants were enrolled and assigned to interventions?			PY	
	1.3 Did baseline differences between intervention groups sµggest a problem with the randomization process?			N	
	**Risk of bias judgement**			**Low**	
**Bias due to deviations from intended interventions**	2.1.Were participants aware of their assigned intervention during the trial?			N	
	2.2.Were carers and people delivering the interventions aware of participants' assigned intervention during the trial?			N	
	2.3. If Y/PY/NI to 2.1 or 2.2: Were there deviations from the intended intervention that arose because of the experimental context?			NA	
	2.4 If Y/PY to 2.3: Were these deviations likely to have affected the outcome?			NA	
	2.5. If Y/PY/NI to 2.4: Were these deviations from intended intervention balanced between groups?			NA	
	2.6 Was an appropriate analysis used to estimate the effect of assignment to intervention?			Y	
	2.7 If N/PN/NI to 2.6: Was there potential for a substantial impact (on the result) of the failure to analyse participants in the group to which they were randomized?			NA	
	**Risk of bias judgement**			**Low**	
**Bias due to missing outcome data**	3.1 Were data for this outcome available for all, or nearly all, participants randomized?			Y	

	3.2 If N/PN/NI to 3.1: Is there evidence that result was not biased by missing outcome data?	NA	
	3.3 If N/PN to 3.2: Could missingness in the outcome depend on its true value?	NA	
	3.4 If Y/PY/NI to 3.3: Is it likely that missingness in the outcome depended on its true value?	NA	
	**Risk of bias judgement**	**Low**	
**Bias in measurement of the outcome**	4.1 Was the method of measuring the outcome inappropriate?	N	
	4.2 Could measurement or ascertainment of the outcome have differed between intervention groups?	N	
	4.3 Were outcome assessors aware of the intervention received by study participants?	N	
	4.4 If Y/PY/NI to 4.3: Could assessment of the outcome have been influenced by knowledge of intervention received?	NA	
	4.5 If Y/PY/NI to 4.4: Is it likely that assessment of the outcome was influenced by knowledge of intervention received?	NA	
	**Risk of bias judgement**	**Low**	
**Bias in selection of the reported result**	5.1 Were the data that produced this result analysed in accordance with a pre-specified analysis plan that was finalized before unblinded outcome data were available for analysis?	Y	
	5.2 ... multiple eligible outcome measurements (e.g. scales, definitions, time points) within the outcome domain?	N	
	5.3 ... multiple eligible analyses of the data?	N	
	**Risk of bias judgement**	**Low**	
**Overall bias**	**Risk of bias judgement**	**Low**	

**Unique ID**	8	**Study ID**	839	**Assessor**	KS TK
**Ref or Label**	Kaiser_2007	**Aim**	assignment to intervention (the 'intention-to-treat' effect)		
**Experimental**	FF	**Comparator**	Placebo	**Source**	Journal article(s)
**Outcome**		**Results**		**Weight**	

Domain	Signalling question	Response	Comments
**Bias arising from the randomization process**	1.1 Was the allocation sequence random?	Y	
	1.2 Was the allocation sequence concealed until participants were enrolled and assigned to interventions?	Y	
	1.3 Did baseline differences between intervention groups sµggest a problem with the randomization process?	N	
	**Risk of bias judgement**	**Low**	
**Bias due to deviations from intended interventions**	2.1.Were participants aware of their assigned intervention during the trial?	N	
	2.2.Were carers and people delivering the interventions aware of participants' assigned intervention during the trial?	N	
	2.3. If Y/PY/NI to 2.1 or 2.2: Were there deviations from the intended intervention that arose because of the experimental context?	NA	
	2.4 If Y/PY to 2.3: Were these deviations likely to have affected the outcome?	NA	
	2.5. If Y/PY/NI to 2.4: Were these deviations from intended intervention balanced between groups?	NA	

	2.6 Was an appropriate analysis used to estimate the effect of assignment to intervention?	Y	
	2.7 If N/PN/NI to 2.6: Was there potential for a substantial impact (on the result) of the failure to analyse participants in the group to which they were randomized?	NA	
	**Risk of bias judgement**	**Low**	
**Bias due to missing outcome data**	3.1 Were data for this outcome available for all, or nearly all, participants randomized?	Y	
	3.2 If N/PN/NI to 3.1: Is there evidence that result was not biased by missing outcome data?	NA	
	3.3 If N/PN to 3.2: Could missingness in the outcome depend on its true value?	NA	
	3.4 If Y/PY/NI to 3.3: Is it likely that missingness in the outcome depended on its true value?	NA	
	**Risk of bias judgement**	**Low**	
**Bias in measurement of the outcome**	4.1 Was the method of measuring the outcome inappropriate?	N	
	4.2 Could measurement or ascertainment of the outcome have differed between intervention groups?	N	
	4.3 Were outcome assessors aware of the intervention received by study participants?	N	
	4.4 If Y/PY/NI to 4.3: Could assessment of the outcome have been influenced by knowledge of intervention received?	NA	
	4.5 If Y/PY/NI to 4.4: Is it likely that assessment of the outcome was influenced by knowledge of intervention received?	NA	
	**Risk of bias judgement**	**Low**	
**Bias in selection of the reported result**	5.1 Were the data that produced this result analysed in accordance with a pre-specified analysis plan that was finalized before unblinded outcome data were available for analysis?	Y	

	5.2 ... multiple eligible outcome measurements (e.g. scales, definitions, time points) within the outcome domain?			N	
	5.3 ... multiple eligible analyses of the data?			N	
	**Risk of bias judgement**			**Low**	
**Overall bias**	**Risk of bias judgement**			**Low**	
**Unique ID**	9	**Study ID**	1238	**Assessor**	KS TK
**Ref or Label**	Fokkens_2007	**Aim**	assignment to intervention (the 'intention-to-treat' effect)		
**Experimental**	FF	**Comparator**	Placebo	**Source**	Journal article(s)
**Outcome**		**Results**		**Weight**	
**Domain**	**Signalling question**			**Response**	**Comments**
**Bias arising from the randomization process**	1.1 Was the allocation sequence random?			PY	
	1.2 Was the allocation sequence concealed until participants were enrolled and assigned to interventions?			PY	
	1.3 Did baseline differences between intervention groups sμggest a problem with the randomization process?			N	
	**Risk of bias judgement**			**Low**	
	2.1.Were participants aware of their assigned intervention during the trial?			N	

**Bias due to deviations from intended interventions**	2.2.Were carers and people delivering the interventions aware of participants' assigned intervention during the trial?	N	
	2.3. If Y/PY/NI to 2.1 or 2.2: Were there deviations from the intended intervention that arose because of the experimental context?	NA	
	2.4 If Y/PY to 2.3: Were these deviations likely to have affected the outcome?	NA	
	2.5. If Y/PY/NI to 2.4: Were these deviations from intended intervention balanced between groups?	NA	
	2.6 Was an appropriate analysis used to estimate the effect of assignment to intervention?	Y	
	2.7 If N/PN/NI to 2.6: Was there potential for a substantial impact (on the result) of the failure to analyse participants in the group to which they were randomized?	NA	
	**Risk of bias judgement**	**Low**	
**Bias due to missing outcome data**	3.1 Were data for this outcome available for all, or nearly all, participants randomized?	Y	
	3.2 If N/PN/NI to 3.1: Is there evidence that result was not biased by missing outcome data?	NA	
	3.3 If N/PN to 3.2: Could missingness in the outcome depend on its true value?	NA	
	3.4 If Y/PY/NI to 3.3: Is it likely that missingness in the outcome depended on its true value?	NA	
	**Risk of bias judgement**	**Low**	
**Bias in measurement of the outcome**	4.1 Was the method of measuring the outcome inappropriate?	N	
	4.2 Could measurement or ascertainment of the outcome have differed between intervention groups?	N	
	4.3 Were outcome assessors aware of the intervention received by study participants?	N	

	4.4 If Y/PY/NI to 4.3: Could assessment of the outcome have been influenced by knowledge of intervention received?			NA	
	4.5 If Y/PY/NI to 4.4: Is it likely that assessment of the outcome was influenced by knowledge of intervention received?			NA	
	**Risk of bias judgement**			**Low**	
**Bias in selection of the reported result**	5.1 Were the data that produced this result analysed in accordance with a pre-specified analysis plan that was finalized before unblinded outcome data were available for analysis?			Y	
	5.2 ... multiple eligible outcome measurements (e.g. scales, definitions, time points) within the outcome domain?			N	
	5.3 ... multiple eligible analyses of the data?			N	
	**Risk of bias judgement**			**Low**	
**Overall bias**	**Risk of bias judgement**			**Low**	
**Unique ID**	10	**Study ID**	575	**Assessor**	KS TK
**Ref or Label**	Ratner_2006	**Aim**	assignment to intervention (the 'intention-to-treat' effect)		
**Experimental**	CIC	**Comparator**	Placebo	**Source**	Journal article(s)
**Outcome**		**Results**		**Weight**	
**Domain**	**Signalling question**			**Response**	**Comments**
**Bias arising from the randomization process**	1.1 Was the allocation sequence random?			PY	

	1.2 Was the allocation sequence concealed until participants were enrolled and assigned to interventions?	PY	
	1.3 Did baseline differences between intervention groups sμggest a problem with the randomization process?	N	
	**Risk of bias judgement**	**Low**	
**Bias due to deviations from intended interventions**	2.1.Were participants aware of their assigned intervention during the trial?	PN	
	2.2.Were carers and people delivering the interventions aware of participants' assigned intervention during the trial?	PN	
	2.3. If Y/PY/NI to 2.1 or 2.2: Were there deviations from the intended intervention that arose because of the experimental context?	NA	
	2.4 If Y/PY to 2.3: Were these deviations likely to have affected the outcome?	NA	
	2.5. If Y/PY/NI to 2.4: Were these deviations from intended intervention balanced between groups?	NA	
	2.6 Was an appropriate analysis used to estimate the effect of assignment to intervention?	Y	
	2.7 If N/PN/NI to 2.6: Was there potential for a substantial impact (on the result) of the failure to analyse participants in the group to which they were randomized?	NA	
	**Risk of bias judgement**	**Low**	
**Bias due to missing outcome data**	3.1 Were data for this outcome available for all, or nearly all, participants randomized?	Y	
	3.2 If N/PN/NI to 3.1: Is there evidence that result was not biased by missing outcome data?	NA	
	3.3 If N/PN to 3.2: Could missingness in the outcome depend on its true value?	NA	
	3.4 If Y/PY/NI to 3.3: Is it likely that missingness in the outcome depended on its true value?	NA	

	**Risk of bias judgement**	**Low**	
**Bias in measurement of the outcome**	4.1 Was the method of measuring the outcome inappropriate?	N	
	4.2 Could measurement or ascertainment of the outcome have differed between intervention groups?	N	
	4.3 Were outcome assessors aware of the intervention received by study participants?	PN	
	4.4 If Y/PY/NI to 4.3: Could assessment of the outcome have been influenced by knowledge of intervention received?	NA	
	4.5 If Y/PY/NI to 4.4: Is it likely that assessment of the outcome was influenced by knowledge of intervention received?	NA	
	**Risk of bias judgement**	**Low**	
**Bias in selection of the reported result**	5.1 Were the data that produced this result analysed in accordance with a pre-specified analysis plan that was finalized before unblinded outcome data were available for analysis?	Y	
	5.2 ... multiple eligible outcome measurements (e.g. scales, definitions, time points) within the outcome domain?	N	
	5.3 ... multiple eligible analyses of the data?	N	
	**Risk of bias judgement**	**Low**	
**Overall bias**	**Risk of bias judgement**	**Low**	

**Unique ID**	11	**Study ID**	1787	**Assessor**	KS TK
**Ref or Label**	Meltzer_2004	**Aim**	assignment to intervention (the 'intention-to-treat' effect)		

**Experimental**	TAA	**Comparator**	FP	**Source**	Journal article(s)
**Outcome**		**Results**		**Weight**	
**Domain**	**Signalling question**			**Response**	**Comments**
**Bias arising from the randomization process**	1.1 Was the allocation sequence random?			PY	
	1.2 Was the allocation sequence concealed until participants were enrolled and assigned to interventions?			PY	
	1.3 Did baseline differences between intervention groups sμggest a problem with the randomization process?			N	
	**Risk of bias judgement**			**Low**	
**Bias due to deviations from intended interventions**	2.1.Were participants aware of their assigned intervention during the trial?			Y	Single (Investigator-blinded)
	2.2.Were carers and people delivering the interventions aware of participants' assigned intervention during the trial?			Y	
	2.3. If Y/PY/NI to 2.1 or 2.2: Were there deviations from the intended intervention that arose because of the experimental context?			N	
	2.4 If Y/PY to 2.3: Were these deviations likely to have affected the outcome?			NA	
	2.5. If Y/PY/NI to 2.4: Were these deviations from intended intervention balanced between groups?			NA	
	2.6 Was an appropriate analysis used to estimate the effect of assignment to intervention?			Y	
	2.7 If N/PN/NI to 2.6: Was there potential for a substantial impact (on the result) of the failure to analyse participants in the group to which they were randomized?			NA	
	**Risk of bias judgement**			**Low**	

**Bias due to missing outcome data**	3.1 Were data for this outcome available for all, or nearly all, participants randomized?	Y	
	3.2 If N/PN/NI to 3.1: Is there evidence that result was not biased by missing outcome data?	NA	
	3.3 If N/PN to 3.2: Could missingness in the outcome depend on its true value?	NA	
	3.4 If Y/PY/NI to 3.3: Is it likely that missingness in the outcome depended on its true value?	NA	
	**Risk of bias judgement**	**Low**	
**Bias in measurement of the outcome**	4.1 Was the method of measuring the outcome inappropriate?	N	
	4.2 Could measurement or ascertainment of the outcome have differed between intervention groups?	N	
	4.3 Were outcome assessors aware of the intervention received by study participants?	N	
	4.4 If Y/PY/NI to 4.3: Could assessment of the outcome have been influenced by knowledge of intervention received?	NA	
	4.5 If Y/PY/NI to 4.4: Is it likely that assessment of the outcome was influenced by knowledge of intervention received?	NA	
	**Risk of bias judgement**	**Low**	
**Bias in selection of the reported result**	5.1 Were the data that produced this result analysed in accordance with a pre-specified analysis plan that was finalized before unblinded outcome data were available for analysis?	Y	
	5.2 ... multiple eligible outcome measurements (e.g. scales, definitions, time points) within the outcome domain?	N	
	5.3 ... multiple eligible analyses of the data?	N	
	**Risk of bias judgement**	**Low**	

Overall bias	Risk of bias judgement			Low	
**Unique ID**	12	**Study ID**	1479	**Assessor**	KS TK
**Ref or Label**	Gawchik _2003	**Aim**	assignment to intervention (the 'intention-to-treat' effect)		
**Experimental**	MF	**Comparator**	Placebo	**Source**	Journal article(s)
**Outcome**		**Results**		**Weight**	
**Domain**	**Signalling question**			**Response**	**Comments**
**Bias arising from the randomization process**	1.1 Was the allocation sequence random?			Y	
	1.2 Was the allocation sequence concealed until participants were enrolled and assigned to interventions?			Y	
	1.3 Did baseline differences between intervention groups sμggest a problem with the randomization process?			N	Slight higher in individual NSS (but not exceed MCID)
	**Risk of bias judgement**			**Low**	
**Bias due to deviations from intended interventions**	2.1.Were participants aware of their assigned intervention during the trial?			N	
	2.2.Were carers and people delivering the interventions aware of participants' assigned intervention during the trial?			N	
	2.3. If Y/PY/NI to 2.1 or 2.2: Were there deviations from the intended intervention that arose because of the experimental context?			NA	
	2.4 If Y/PY to 2.3: Were these deviations likely to have affected the outcome?			NA	

	2.5. If Y/PY/NI to 2.4: Were these deviations from intended intervention balanced between groups?	NA	
	2.6 Was an appropriate analysis used to estimate the effect of assignment to intervention?	Y	
	2.7 If N/PN/NI to 2.6: Was there potential for a substantial impact (on the result) of the failure to analyse participants in the group to which they were randomized?	NA	
	**Risk of bias judgement**	**Low**	
**Bias due to missing outcome data**	3.1 Were data for this outcome available for all, or nearly all, participants randomized?	Y	
	3.2 If N/PN/NI to 3.1: Is there evidence that result was not biased by missing outcome data?	NA	
	3.3 If N/PN to 3.2: Could missingness in the outcome depend on its true value?	NA	
	3.4 If Y/PY/NI to 3.3: Is it likely that missingness in the outcome depended on its true value?	NA	
	**Risk of bias judgement**	**Low**	
**Bias in measurement of the outcome**	4.1 Was the method of measuring the outcome inappropriate?	N	
	4.2 Could measurement or ascertainment of the outcome have differed between intervention groups?	N	
	4.3 Were outcome assessors aware of the intervention received by study participants?	N	
	4.4 If Y/PY/NI to 4.3: Could assessment of the outcome have been influenced by knowledge of intervention received?	NA	
	4.5 If Y/PY/NI to 4.4: Is it likely that assessment of the outcome was influenced by knowledge of intervention received?	NA	
	**Risk of bias judgement**	**Low**	

**Bias in selection of the reported result**	5.1 Were the data that produced this result analysed in accordance with a pre-specified analysis plan that was finalized before unblinded outcome data were available for analysis?			Y	
	5.2 ... multiple eligible outcome measurements (e.g. scales, definitions, time points) within the outcome domain?			N	
	5.3 ... multiple eligible analyses of the data?			N	
	**Risk of bias judgement**			**Low**	
**Overall bias**	**Risk of bias judgement**			**Low**	
**Unique ID**	13	**Study ID**	270	**Assessor**	KS TK
**Ref or Label**	Lumry_2003	**Aim**	assignment to intervention (the 'intention-to-treat' effect)		
**Experimental**	TA	**Comparator**	BDP	**Source**	Journal article(s)
**Outcome**		**Results**		**Weight**	
**Domain**	**Signalling question**			**Response**	**Comments**
**Bias arising from the randomization process**	1.1 Was the allocation sequence random?			Y	
	1.2 Was the allocation sequence concealed until participants were enrolled and assigned to interventions?			Y	
	1.3 Did baseline differences between intervention groups sμggest a problem with the randomization process?			N	
	**Risk of bias judgement**			**Low**	

Bias due to deviations from intended interventions	2.1.Were participants aware of their assigned intervention during the trial?	PY	
	2.2.Were carers and people delivering the interventions aware of participants' assigned intervention during the trial?	PY	
	2.3. If Y/PY/NI to 2.1 or 2.2: Were there deviations from the intended intervention that arose because of the experimental context?	N	
	2.4 If Y/PY to 2.3: Were these deviations likely to have affected the outcome?	NA	
	2.5. If Y/PY/NI to 2.4: Were these deviations from intended intervention balanced between groups?	NA	
	2.6 Was an appropriate analysis used to estimate the effect of assignment to intervention?	Y	
	2.7 If N/PN/NI to 2.6: Was there potential for a substantial impact (on the result) of the failure to analyse participants in the group to which they were randomized?	NA	
	**Risk of bias judgement**	**Low**	
**Bias due to missing outcome data**	3.1 Were data for this outcome available for all, or nearly all, participants randomized?	Y	
	3.2 If N/PN/NI to 3.1: Is there evidence that result was not biased by missing outcome data?	NA	
	3.3 If N/PN to 3.2: Could missingness in the outcome depend on its true value?	NA	
	3.4 If Y/PY/NI to 3.3: Is it likely that missingness in the outcome depended on its true value?	NA	
	**Risk of bias judgement**	**Low**	
**Bias in measurement of the outcome**	4.1 Was the method of measuring the outcome inappropriate?	N	
	4.2 Could measurement or ascertainment of the outcome have differed between intervention groups?	N	

	4.3 Were outcome assessors aware of the intervention received by study participants?			N	
	4.4 If Y/PY/NI to 4.3: Could assessment of the outcome have been influenced by knowledge of intervention received?			NA	
	4.5 If Y/PY/NI to 4.4: Is it likely that assessment of the outcome was influenced by knowledge of intervention received?			NA	
	**Risk of bias judgement**			**Low**	
**Bias in selection of the reported result**	5.1 Were the data that produced this result analysed in accordance with a pre-specified analysis plan that was finalized before unblinded outcome data were available for analysis?			Y	
	5.2 ... multiple eligible outcome measurements (e.g. scales, definitions, time points) within the outcome domain?			N	
	5.3 ... multiple eligible analyses of the data?			N	
	**Risk of bias judgement**			**Low**	
**Overall bias**	**Risk of bias judgement**			**Low**	
**Unique ID**	14	**Study ID**	1789	**Assessor**	KS TK
**Ref or Label**	Berger_2003	**Aim**	assignment to intervention (the 'intention-to-treat' effect)		
**Experimental**	TA	**Comparator**	FP	**Source**	Journal article(s)
**Outcome**		**Results**		**Weight**	
**Domain**	**Signalling question**			**Response**	**Comments**

**Bias arising from the randomization process**	1.1 Was the allocation sequence random?	PY	
	1.2 Was the allocation sequence concealed until participants were enrolled and assigned to interventions?	PY	
	1.3 Did baseline differences between intervention groups sμggest a problem with the randomization process?	N	slight difference in individual NSS + TNSS but no MCID
	**Risk of bias judgement**	**Low**	
**Bias due to deviations from intended interventions**	2.1.Were participants aware of their assigned intervention during the trial?	Y	Investigator-blinded
	2.2.Were carers and people delivering the interventions aware of participants' assigned intervention during the trial?	Y	
	2.3. If Y/PY/NI to 2.1 or 2.2: Were there deviations from the intended intervention that arose because of the experimental context?	N	
	2.4 If Y/PY to 2.3: Were these deviations likely to have affected the outcome?	NA	
	2.5. If Y/PY/NI to 2.4: Were these deviations from intended intervention balanced between groups?	NA	
	2.6 Was an appropriate analysis used to estimate the effect of assignment to intervention?	Y	
	2.7 If N/PN/NI to 2.6: Was there potential for a substantial impact (on the result) of the failure to analyse participants in the group to which they were randomized?	NA	
	**Risk of bias judgement**	**Low**	
**Bias due to missing outcome data**	3.1 Were data for this outcome available for all, or nearly all, participants randomized?	Y	
	3.2 If N/PN/NI to 3.1: Is there evidence that result was not biased by missing outcome data?	NA	
	3.3 If N/PN to 3.2: Could missingness in the outcome depend on its true value?	NA	

	3.4 If Y/PY/NI to 3.3: Is it likely that missingness in the outcome depended on its true value?	NA	
	**Risk of bias judgement**	**Low**	
**Bias in measurement of the outcome**	4.1 Was the method of measuring the outcome inappropriate?	N	
	4.2 Could measurement or ascertainment of the outcome have differed between intervention groups?	N	
	4.3 Were outcome assessors aware of the intervention received by study participants?	N	
	4.4 If Y/PY/NI to 4.3: Could assessment of the outcome have been influenced by knowledge of intervention received?	NA	
	4.5 If Y/PY/NI to 4.4: Is it likely that assessment of the outcome was influenced by knowledge of intervention received?	NA	
	**Risk of bias judgement**	**Low**	
**Bias in selection of the reported result**	5.1 Were the data that produced this result analysed in accordance with a pre-specified analysis plan that was finalized before unblinded outcome data were available for analysis?	Y	
	5.2 ... multiple eligible outcome measurements (e.g. scales, definitions, time points) within the outcome domain?	N	
	5.3 ... multiple eligible analyses of the data?	N	
	**Risk of bias judgement**	**Low**	
**Overall bias**	**Risk of bias judgement**	**Low**	

**Unique ID**	15	**Study ID**	223	**Assessor**	KS TK

**Ref or Label**	Gross_2002	**Aim**	assignment to intervention (the 'intention-to-treat' effect)		
**Experimental**	TAA	**Comparator**	FP	**Source**	Journal article(s)
**Outcome**		**Results**		**Weight**	
**Domain**	**Signalling question**			**Response**	**Comments**
**Bias arising from the randomization process**	1.1 Was the allocation sequence random?			Y	
	1.2 Was the allocation sequence concealed until participants were enrolled and assigned to interventions?			Y	
	1.3 Did baseline differences between intervention groups sµggest a problem with the randomization process?			N	
	**Risk of bias judgement**			**Low**	
**Bias due to deviations from intended interventions**	2.1.Were participants aware of their assigned intervention during the trial?			N	
	2.2.Were carers and people delivering the interventions aware of participants' assigned intervention during the trial?			N	
	2.3. If Y/PY/NI to 2.1 or 2.2: Were there deviations from the intended intervention that arose because of the experimental context?			NA	
	2.4 If Y/PY to 2.3: Were these deviations likely to have affected the outcome?			NA	
	2.5. If Y/PY/NI to 2.4: Were these deviations from intended intervention balanced between groups?			NA	
	2.6 Was an appropriate analysis used to estimate the effect of assignment to intervention?			Y	
	2.7 If N/PN/NI to 2.6: Was there potential for a substantial impact (on the result) of the failure to analyse participants in the group to which they were randomized?			NA	

	**Risk of bias judgement**	**Low**	
**Bias due to missing outcome data**	3.1 Were data for this outcome available for all, or nearly all, participants randomized?	Y	
	3.2 If N/PN/NI to 3.1: Is there evidence that result was not biased by missing outcome data?	NA	
	3.3 If N/PN to 3.2: Could missingness in the outcome depend on its true value?	NA	
	3.4 If Y/PY/NI to 3.3: Is it likely that missingness in the outcome depended on its true value?	NA	
	**Risk of bias judgement**	**Low**	
**Bias in measurement of the outcome**	4.1 Was the method of measuring the outcome inappropriate?	N	
	4.2 Could measurement or ascertainment of the outcome have differed between intervention groups?	N	
	4.3 Were outcome assessors aware of the intervention received by study participants?	N	
	4.4 If Y/PY/NI to 4.3: Could assessment of the outcome have been influenced by knowledge of intervention received?	NA	
	4.5 If Y/PY/NI to 4.4: Is it likely that assessment of the outcome was influenced by knowledge of intervention received?	NA	
	**Risk of bias judgement**	**Low**	
**Bias in selection of the reported result**	5.1 Were the data that produced this result analysed in accordance with a pre-specified analysis plan that was finalized before unblinded outcome data were available for analysis?	Y	
	5.2 ... multiple eligible outcome measurements (e.g. scales, definitions, time points) within the outcome domain?	N	
	5.3 ... multiple eligible analyses of the data?	N	

	**Risk of bias judgement**			**Low**	
**Overall bias**	**Risk of bias judgement**			**Low**	
**Unique ID**	16	**Study ID**	1677	**Assessor**	KS TK
**Ref or Label**	Meltzer_1998	**Aim**	assignment to intervention (the 'intention-to-treat' effect)		
**Experimental**	MF	**Comparator**	Placebo	**Source**	Journal article(s)
**Outcome**		**Results**		**Weight**	
**Domain**	**Signalling question**			**Response**	**Comments**
**Bias arising from the randomization process**	1.1 Was the allocation sequence random?			PY	
	1.2 Was the allocation sequence concealed until participants were enrolled and assigned to interventions?			PY	
	1.3 Did baseline differences between intervention groups sµggest a problem with the randomization process?			N	
	**Risk of bias judgement**			**Low**	
**Bias due to deviations from intended interventions**	2.1.Were participants aware of their assigned intervention during the trial?			N	
	2.2.Were carers and people delivering the interventions aware of participants' assigned intervention during the trial?			N	
	2.3. If Y/PY/NI to 2.1 or 2.2: Were there deviations from the intended intervention that arose because of the experimental context?			NA	

	2.4 If Y/PY to 2.3: Were these deviations likely to have affected the outcome?	NA	
	2.5. If Y/PY/NI to 2.4: Were these deviations from intended intervention balanced between groups?	NA	
	2.6 Was an appropriate analysis used to estimate the effect of assignment to intervention?	Y	
	2.7 If N/PN/NI to 2.6: Was there potential for a substantial impact (on the result) of the failure to analyse participants in the group to which they were randomized?	NA	
	**Risk of bias judgement**	**Low**	
**Bias due to missing outcome data**	3.1 Were data for this outcome available for all, or nearly all, participants randomized?	Y	
	3.2 If N/PN/NI to 3.1: Is there evidence that result was not biased by missing outcome data?	NA	
	3.3 If N/PN to 3.2: Could missingness in the outcome depend on its true value?	NA	
	3.4 If Y/PY/NI to 3.3: Is it likely that missingness in the outcome depended on its true value?	NA	
	**Risk of bias judgement**	**Low**	
**Bias in measurement of the outcome**	4.1 Was the method of measuring the outcome inappropriate?	N	
	4.2 Could measurement or ascertainment of the outcome have differed between intervention groups?	N	
	4.3 Were outcome assessors aware of the intervention received by study participants?	N	
	4.4 If Y/PY/NI to 4.3: Could assessment of the outcome have been influenced by knowledge of intervention received?	NA	
	4.5 If Y/PY/NI to 4.4: Is it likely that assessment of the outcome was influenced by knowledge of intervention received?	NA	

	**Risk of bias judgement**			**Low**	
**Bias in selection of the reported result**	5.1 Were the data that produced this result analysed in accordance with a pre-specified analysis plan that was finalized before unblinded outcome data were available for analysis?			Y	
	5.2 ... multiple eligible outcome measurements (e.g. scales, definitions, time points) within the outcome domain?			N	
	5.3 ... multiple eligible analyses of the data?			N	
	**Risk of bias judgement**			**Low**	
**Overall bias**	**Risk of bias judgement**			**Low**	
**Unique ID**	17	**Study ID**	849	**Assessor**	KS TK
**Ref or Label**	Bronsky_1996	**Aim**	assignment to intervention (the 'intention-to-treat' effect)		
**Experimental**	FP	**Comparator**	Ter/Placebo	**Source**	Journal article(s)
**Outcome**		**Results**		**Weight**	
**Domain**	**Signalling question**			**Response**	**Comments**
**Bias arising from the randomization process**	1.1 Was the allocation sequence random?			PY	
	1.2 Was the allocation sequence concealed until participants were enrolled and assigned to interventions?			PY	
	1.3 Did baseline differences between intervention groups sμggest a problem with the randomization process?			N	

	**Risk of bias judgement**	**Low**	
**Bias due to deviations from intended interventions**	2.1.Were participants aware of their assigned intervention during the trial?	N	
	2.2.Were carers and people delivering the interventions aware of participants' assigned intervention during the trial?	N	
	2.3. If Y/PY/NI to 2.1 or 2.2: Were there deviations from the intended intervention that arose because of the experimental context?	NA	
	2.4 If Y/PY to 2.3: Were these deviations likely to have affected the outcome?	NA	
	2.5. If Y/PY/NI to 2.4: Were these deviations from intended intervention balanced between groups?	NA	
	2.6 Was an appropriate analysis used to estimate the effect of assignment to intervention?	Y	
	2.7 If N/PN/NI to 2.6: Was there potential for a substantial impact (on the result) of the failure to analyse participants in the group to which they were randomized?	NA	
	**Risk of bias judgement**	**Low**	
**Bias due to missing outcome data**	3.1 Were data for this outcome available for all, or nearly all, participants randomized?	Y	
	3.2 If N/PN/NI to 3.1: Is there evidence that result was not biased by missing outcome data?	NA	
	3.3 If N/PN to 3.2: Could missingness in the outcome depend on its true value?	NA	
	3.4 If Y/PY/NI to 3.3: Is it likely that missingness in the outcome depended on its true value?	NA	
	**Risk of bias judgement**	**Low**	
**Bias in measurement of the outcome**	4.1 Was the method of measuring the outcome inappropriate?	N	

	4.2 Could measurement or ascertainment of the outcome have differed between intervention groups?			N	
	4.3 Were outcome assessors aware of the intervention received by study participants?			N	
	4.4 If Y/PY/NI to 4.3: Could assessment of the outcome have been influenced by knowledge of intervention received?			NA	
	4.5 If Y/PY/NI to 4.4: Is it likely that assessment of the outcome was influenced by knowledge of intervention received?			NA	
	**Risk of bias judgement**			**Low**	
**Bias in selection of the reported result**	5.1 Were the data that produced this result analysed in accordance with a pre-specified analysis plan that was finalized before unblinded outcome data were available for analysis?			Y	
	5.2 ... multiple eligible outcome measurements (e.g. scales, definitions, time points) within the outcome domain?			N	
	5.3 ... multiple eligible analyses of the data?			N	
	**Risk of bias judgement**			**Low**	
**Overall bias**	**Risk of bias judgement**			**Low**	
**Unique ID**	18	**Study ID**	1000	**Assessor**	KS TK
**Ref or Label**	Bavel_1994	**Aim**	assignment to intervention (the 'intention-to-treat' effect)		
**Experimental**	FP	**Comparator**	Ter/Placebo	**Source**	Journal article(s)
**Outcome**		**Results**		**Weight**	

Domain	Signalling question	Response	Comments
**Bias arising from the randomization process**	1.1 Was the allocation sequence random?	PY	
	1.2 Was the allocation sequence concealed until participants were enrolled and assigned to interventions?	PY	
	1.3 Did baseline differences between intervention groups sµggest a problem with the randomization process?	N	
	**Risk of bias judgement**	**Low**	
**Bias due to deviations from intended interventions**	2.1.Were participants aware of their assigned intervention during the trial?	N	
	2.2.Were carers and people delivering the interventions aware of participants' assigned intervention during the trial?	N	
	2.3. If Y/PY/NI to 2.1 or 2.2: Were there deviations from the intended intervention that arose because of the experimental context?	NA	
	2.4 If Y/PY to 2.3: Were these deviations likely to have affected the outcome?	NA	
	2.5. If Y/PY/NI to 2.4: Were these deviations from intended intervention balanced between groups?	NA	
	2.6 Was an appropriate analysis used to estimate the effect of assignment to intervention?	Y	
	2.7 If N/PN/NI to 2.6: Was there potential for a substantial impact (on the result) of the failure to analyse participants in the group to which they were randomized?	NA	
	**Risk of bias judgement**	**Low**	
**Bias due to missing outcome data**	3.1 Were data for this outcome available for all, or nearly all, participants randomized?	Y	
	3.2 If N/PN/NI to 3.1: Is there evidence that result was not biased by missing outcome data?	NA	

	3.3 If N/PN to 3.2: Could missingness in the outcome depend on its true value?	NA	
	3.4 If Y/PY/NI to 3.3: Is it likely that missingness in the outcome depended on its true value?	NA	
	**Risk of bias judgement**	**Low**	
**Bias in measurement of the outcome**	4.1 Was the method of measuring the outcome inappropriate?	N	
	4.2 Could measurement or ascertainment of the outcome have differed between intervention groups?	N	
	4.3 Were outcome assessors aware of the intervention received by study participants?	N	
	4.4 If Y/PY/NI to 4.3: Could assessment of the outcome have been influenced by knowledge of intervention received?	NA	
	4.5 If Y/PY/NI to 4.4: Is it likely that assessment of the outcome was influenced by knowledge of intervention received?	NA	
	**Risk of bias judgement**	**Low**	
**Bias in selection of the reported result**	5.1 Were the data that produced this result analysed in accordance with a pre-specified analysis plan that was finalized before unblinded outcome data were available for analysis?	Y	
	5.2 ... multiple eligible outcome measurements (e.g. scales, definitions, time points) within the outcome domain?	N	
	5.3 ... multiple eligible analyses of the data?	N	
	**Risk of bias judgement**	**Low**	
**Overall bias**	**Risk of bias judgement**	**Low**	

Unique ID	19	Study ID	853	Assessor	KS TK
Ref or Label	Ratner_1992	Aim	assignment to intervention (the 'intention-to-treat' effect)		
Experimental	FP	Comparator	BDP/Placebo	Source	Journal article(s)
Outcome		Results		Weight	

Domain	Signalling question	Response	Comments
Bias arising from the randomization process	1.1 Was the allocation sequence random?	PY	
	1.2 Was the allocation sequence concealed until participants were enrolled and assigned to interventions?	PY	
	1.3 Did baseline differences between intervention groups sμggest a problem with the randomization process?	N	
	**Risk of bias judgement**	**Low**	
Bias due to deviations from intended interventions	2.1.Were participants aware of their assigned intervention during the trial?	N	
	2.2.Were carers and people delivering the interventions aware of participants' assigned intervention during the trial?	N	
	2.3. If Y/PY/NI to 2.1 or 2.2: Were there deviations from the intended intervention that arose because of the experimental context?	NA	
	2.4 If Y/PY to 2.3: Were these deviations likely to have affected the outcome?	NA	
	2.5. If Y/PY/NI to 2.4: Were these deviations from intended intervention balanced between groups?	NA	
	2.6 Was an appropriate analysis used to estimate the effect of assignment to intervention?	Y	

	2.7 If N/PN/NI to 2.6: Was there potential for a substantial impact (on the result) of the failure to analyse participants in the group to which they were randomized?	NA	
	**Risk of bias judgement**	**Low**	
**Bias due to missing outcome data**	3.1 Were data for this outcome available for all, or nearly all, participants randomized?	Y	
	3.2 If N/PN/NI to 3.1: Is there evidence that result was not biased by missing outcome data?	NA	
	3.3 If N/PN to 3.2: Could missingness in the outcome depend on its true value?	NA	
	3.4 If Y/PY/NI to 3.3: Is it likely that missingness in the outcome depended on its true value?	NA	
	**Risk of bias judgement**	**Low**	
**Bias in measurement of the outcome**	4.1 Was the method of measuring the outcome inappropriate?	N	
	4.2 Could measurement or ascertainment of the outcome have differed between intervention groups?	N	
	4.3 Were outcome assessors aware of the intervention received by study participants?	N	
	4.4 If Y/PY/NI to 4.3: Could assessment of the outcome have been influenced by knowledge of intervention received?	NA	
	4.5 If Y/PY/NI to 4.4: Is it likely that assessment of the outcome was influenced by knowledge of intervention received?	NA	
	**Risk of bias judgement**	**Low**	
**Bias in selection of the reported result**	5.1 Were the data that produced this result analysed in accordance with a pre-specified analysis plan that was finalized before unblinded outcome data were available for analysis?	Y	
	5.2 ... multiple eligible outcome measurements (e.g. scales, definitions, time points) within the outcome domain?	N	

	5.3 ... multiple eligible analyses of the data?			N	
	**Risk of bias judgement**			**Low**	
**Overall bias**	**Risk of bias judgement**			**Low**	
**Unique ID**	21	**Study ID**	1794	**Assessor**	KS TK
**Ref or Label**	Karaulov_2019	**Aim**	assignment to intervention (the 'intention-to-treat' effect)		
**Experimental**	TA	**Comparator**	FP	**Source**	Journal article(s)
**Outcome**		**Results**		**Weight**	
**Domain**	**Signalling question**			**Response**	**Comments**
**Bias arising from the randomization process**	1.1 Was the allocation sequence random?			PY	
	1.2 Was the allocation sequence concealed until participants were enrolled and assigned to interventions?			PY	
	1.3 Did baseline differences between intervention groups sμggest a problem with the randomization process?			N	
	**Risk of bias judgement**			**Low**	
**Bias due to deviations from intended interventions**	2.1.Were participants aware of their assigned intervention during the trial?			N	
	2.2.Were carers and people delivering the interventions aware of participants' assigned intervention during the trial?			N	

	2.3. If Y/PY/NI to 2.1 or 2.2: Were there deviations from the intended intervention that arose because of the experimental context?	NA	
	2.4 If Y/PY to 2.3: Were these deviations likely to have affected the outcome?	NA	
	2.5. If Y/PY/NI to 2.4: Were these deviations from intended intervention balanced between groups?	NA	
	2.6 Was an appropriate analysis used to estimate the effect of assignment to intervention?	Y	
	2.7 If N/PN/NI to 2.6: Was there potential for a substantial impact (on the result) of the failure to analyse participants in the group to which they were randomized?	NA	
	**Risk of bias judgement**	**Low**	
**Bias due to missing outcome data**	3.1 Were data for this outcome available for all, or nearly all, participants randomized?	Y	
	3.2 If N/PN/NI to 3.1: Is there evidence that result was not biased by missing outcome data?	NA	
	3.3 If N/PN to 3.2: Could missingness in the outcome depend on its true value?	NA	
	3.4 If Y/PY/NI to 3.3: Is it likely that missingness in the outcome depended on its true value?	NA	
	**Risk of bias judgement**	**Low**	
**Bias in measurement of the outcome**	4.1 Was the method of measuring the outcome inappropriate?	N	
	4.2 Could measurement or ascertainment of the outcome have differed between intervention groups?	N	
	4.3 Were outcome assessors aware of the intervention received by study participants?	N	
	4.4 If Y/PY/NI to 4.3: Could assessment of the outcome have been influenced by knowledge of intervention received?	NA	

	4.5 If Y/PY/NI to 4.4: Is it likely that assessment of the outcome was influenced by knowledge of intervention received?			NA	
	**Risk of bias judgement**			**Low**	
**Bias in selection of the reported result**	5.1 Were the data that produced this result analysed in accordance with a pre-specified analysis plan that was finalized before unblinded outcome data were available for analysis?			Y	
	5.2 ... multiple eligible outcome measurements (e.g. scales, definitions, time points) within the outcome domain?			N	
	5.3 ... multiple eligible analyses of the data?			N	
	**Risk of bias judgement**			**Low**	
**Overall bias**	**Risk of bias judgement**			**Low**	
**Unique ID**	22	**Study ID**	1008	**Assessor**	KS TK
**Ref or Label**	Meltzer_2010	**Aim**	assignment to intervention (the 'intention-to-treat' effect)		
**Experimental**	MF	**Comparator**	Placebo	**Source**	Journal article(s)
**Outcome**		**Results**		**Weight**	
**Domain**	**Signalling question**			**Response**	**Comments**
**Bias arising from the randomization process**	1.1 Was the allocation sequence random?			PY	
	1.2 Was the allocation sequence concealed until participants were enrolled and assigned to interventions?			PY	

	1.3 Did baseline differences between intervention groups sµggest a problem with the randomization process?	PY	AHI was lower in MF group than placebo
	**Risk of bias judgement**	**Some concerns**	
**Bias due to deviations from intended interventions**	2.1.Were participants aware of their assigned intervention during the trial?	PN	
	2.2.Were carers and people delivering the interventions aware of participants' assigned intervention during the trial?	PN	
	2.3. If Y/PY/NI to 2.1 or 2.2: Were there deviations from the intended intervention that arose because of the experimental context?	NA	
	2.4 If Y/PY to 2.3: Were these deviations likely to have affected the outcome?	NA	
	2.5. If Y/PY/NI to 2.4: Were these deviations from intended intervention balanced between groups?	NA	
	2.6 Was an appropriate analysis used to estimate the effect of assignment to intervention?	Y	
	2.7 If N/PN/NI to 2.6: Was there potential for a substantial impact (on the result) of the failure to analyse participants in the group to which they were randomized?	NA	
	**Risk of bias judgement**	**Low**	
**Bias due to missing outcome data**	3.1 Were data for this outcome available for all, or nearly all, participants randomized?	Y	
	3.2 If N/PN/NI to 3.1: Is there evidence that result was not biased by missing outcome data?	NA	
	3.3 If N/PN to 3.2: Could missingness in the outcome depend on its true value?	NA	
	3.4 If Y/PY/NI to 3.3: Is it likely that missingness in the outcome depended on its true value?	NA	
	**Risk of bias judgement**	**Low**	

**Bias in measurement of the outcome**	4.1 Was the method of measuring the outcome inappropriate?	N	
	4.2 Could measurement or ascertainment of the outcome have differed between intervention groups?	N	
	4.3 Were outcome assessors aware of the intervention received by study participants?	N	
	4.4 If Y/PY/NI to 4.3: Could assessment of the outcome have been influenced by knowledge of intervention received?	NA	
	4.5 If Y/PY/NI to 4.4: Is it likely that assessment of the outcome was influenced by knowledge of intervention received?	NA	
	**Risk of bias judgement**	**Low**	
**Bias in selection of the reported result**	5.1 Were the data that produced this result analysed in accordance with a pre-specified analysis plan that was finalized before unblinded outcome data were available for analysis?	Y	
	5.2 ... multiple eligible outcome measurements (e.g. scales, definitions, time points) within the outcome domain?	N	
	5.3 ... multiple eligible analyses of the data?	N	
	**Risk of bias judgement**	**Low**	
**Overall bias**	**Risk of bias judgement**	**Some concerns**	Apnea-hypopnea index in MF group was lower than placebo group.

**Unique ID**	23	**Study ID**	836	**Assessor**	KS TK
**Ref or Label**	Given_2010	**Aim**	assignment to intervention (the 'intention-to-treat' effect)		

Experimental	FF	Comparator	Placebo	Source	Journal article(s)
**Outcome**		**Results**		**Weight**	

Domain	Signalling question	Response	Comments
**Bias arising from the randomization process**	1.1 Was the allocation sequence random?	PY	
	1.2 Was the allocation sequence concealed until participants were enrolled and assigned to interventions?	PY	
	1.3 Did baseline differences between intervention groups sµggest a problem with the randomization process?	N	
	**Risk of bias judgement**	**Low**	
**Bias due to deviations from intended interventions**	2.1.Were participants aware of their assigned intervention during the trial?	PN	
	2.2.Were carers and people delivering the interventions aware of participants' assigned intervention during the trial?	PN	
	2.3. If Y/PY/NI to 2.1 or 2.2: Were there deviations from the intended intervention that arose because of the experimental context?	NA	
	2.4 If Y/PY to 2.3: Were these deviations likely to have affected the outcome?	NA	
	2.5. If Y/PY/NI to 2.4: Were these deviations from intended intervention balanced between groups?	NA	
	2.6 Was an appropriate analysis used to estimate the effect of assignment to intervention?	Y	
	2.7 If N/PN/NI to 2.6: Was there potential for a substantial impact (on the result) of the failure to analyse participants in the group to which they were randomized?	NA	
	**Risk of bias judgement**	**Low**	

**Bias due to missing outcome data**	3.1 Were data for this outcome available for all, or nearly all, participants randomized?	Y	
	3.2 If N/PN/NI to 3.1: Is there evidence that result was not biased by missing outcome data?	NA	
	3.3 If N/PN to 3.2: Could missingness in the outcome depend on its true value?	NA	
	3.4 If Y/PY/NI to 3.3: Is it likely that missingness in the outcome depended on its true value?	NA	
	**Risk of bias judgement**	**Low**	
**Bias in measurement of the outcome**	4.1 Was the method of measuring the outcome inappropriate?	N	
	4.2 Could measurement or ascertainment of the outcome have differed between intervention groups?	N	
	4.3 Were outcome assessors aware of the intervention received by study participants?	N	
	4.4 If Y/PY/NI to 4.3: Could assessment of the outcome have been influenced by knowledge of intervention received?	NA	
	4.5 If Y/PY/NI to 4.4: Is it likely that assessment of the outcome was influenced by knowledge of intervention received?	NA	
	**Risk of bias judgement**	**Low**	
**Bias in selection of the reported result**	5.1 Were the data that produced this result analysed in accordance with a pre-specified analysis plan that was finalized before unblinded outcome data were available for analysis?	Y	
	5.2 ... multiple eligible outcome measurements (e.g. scales, definitions, time points) within the outcome domain?	N	
	5.3 ... multiple eligible analyses of the data?	N	
	**Risk of bias judgement**	**Low**	

Overall bias	Risk of bias judgement			Low	
**Unique ID**	24	**Study ID**	535	**Assessor**	KS TK
**Ref or Label**	Baena-Cagnani_2010	**Aim**	assignment to intervention (the 'intention-to-treat' effect)		
**Experimental**	MF	**Comparator**	Placebo	**Source**	Journal article(s)
**Outcome**		**Results**		**Weight**	
**Domain**	**Signalling question**			**Response**	**Comments**
**Bias arising from the randomization process**	1.1 Was the allocation sequence random?			PY	
	1.2 Was the allocation sequence concealed until participants were enrolled and assigned to interventions?			PY	
	1.3 Did baseline differences between intervention groups sµggest a problem with the randomization process?			N	
	**Risk of bias judgement**			**Low**	
**Bias due to deviations from intended interventions**	2.1.Were participants aware of their assigned intervention during the trial?			PN	
	2.2.Were carers and people delivering the interventions aware of participants' assigned intervention during the trial?			PN	
	2.3. If Y/PY/NI to 2.1 or 2.2: Were there deviations from the intended intervention that arose because of the experimental context?			NA	
	2.4 If Y/PY to 2.3: Were these deviations likely to have affected the outcome?			NA	

	2.5. If Y/PY/NI to 2.4: Were these deviations from intended intervention balanced between groups?	NA	
	2.6 Was an appropriate analysis used to estimate the effect of assignment to intervention?	Y	
	2.7 If N/PN/NI to 2.6: Was there potential for a substantial impact (on the result) of the failure to analyse participants in the group to which they were randomized?	NA	
	**Risk of bias judgement**	**Low**	
**Bias due to missing outcome data**	3.1 Were data for this outcome available for all, or nearly all, participants randomized?	Y	
	3.2 If N/PN/NI to 3.1: Is there evidence that result was not biased by missing outcome data?	NA	
	3.3 If N/PN to 3.2: Could missingness in the outcome depend on its true value?	NA	
	3.4 If Y/PY/NI to 3.3: Is it likely that missingness in the outcome depended on its true value?	NA	
	**Risk of bias judgement**	**Low**	
**Bias in measurement of the outcome**	4.1 Was the method of measuring the outcome inappropriate?	N	
	4.2 Could measurement or ascertainment of the outcome have differed between intervention groups?	N	
	4.3 Were outcome assessors aware of the intervention received by study participants?	N	
	4.4 If Y/PY/NI to 4.3: Could assessment of the outcome have been influenced by knowledge of intervention received?	NA	
	4.5 If Y/PY/NI to 4.4: Is it likely that assessment of the outcome was influenced by knowledge of intervention received?	NA	
	**Risk of bias judgement**	**Low**	

**Bias in selection of the reported result**	5.1 Were the data that produced this result analysed in accordance with a pre-specified analysis plan that was finalized before unblinded outcome data were available for analysis?			Y	
	5.2 ... multiple eligible outcome measurements (e.g. scales, definitions, time points) within the outcome domain?			N	
	5.3 ... multiple eligible analyses of the data?			N	
	**Risk of bias judgement**			**Low**	
**Overall bias**	**Risk of bias judgement**			**Low**	
**Unique ID**	25	**Study ID**	9987	**Assessor**	KS TK
**Ref or Label**	Weinstein_2009	**Aim**	assignment to intervention (the 'intention-to-treat' effect)		
**Experimental**	TAA	**Comparator**	Placebo	**Source**	Journal article(s)
**Outcome**		**Results**		**Weight**	
**Domain**	**Signalling question**			**Response**	**Comments**
**Bias arising from the randomization process**	1.1 Was the allocation sequence random?			Y	
	1.2 Was the allocation sequence concealed until participants were enrolled and assigned to interventions?			Y	
	1.3 Did baseline differences between intervention groups sμggest a problem with the randomization process?			N	
	**Risk of bias judgement**			**Low**	

**Bias due to deviations from intended interventions**	2.1.Were participants aware of their assigned intervention during the trial?	N	
	2.2.Were carers and people delivering the interventions aware of participants' assigned intervention during the trial?	N	
	2.3. If Y/PY/NI to 2.1 or 2.2: Were there deviations from the intended intervention that arose because of the experimental context?	NA	
	2.4 If Y/PY to 2.3: Were these deviations likely to have affected the outcome?	NA	
	2.5. If Y/PY/NI to 2.4: Were these deviations from intended intervention balanced between groups?	NA	
	2.6 Was an appropriate analysis used to estimate the effect of assignment to intervention?	Y	
	2.7 If N/PN/NI to 2.6: Was there potential for a substantial impact (on the result) of the failure to analyse participants in the group to which they were randomized?	NA	
	**Risk of bias judgement**	**Low**	
**Bias due to missing outcome data**	3.1 Were data for this outcome available for all, or nearly all, participants randomized?	N	
	3.2 If N/PN/NI to 3.1: Is there evidence that result was not biased by missing outcome data?	Y	
	3.3 If N/PN to 3.2: Could missingness in the outcome depend on its true value?	NA	
	3.4 If Y/PY/NI to 3.3: Is it likely that missingness in the outcome depended on its true value?	NA	
	**Risk of bias judgement**	**Low**	
**Bias in measurement of the outcome**	4.1 Was the method of measuring the outcome inappropriate?	N	
	4.2 Could measurement or ascertainment of the outcome have differed between intervention groups?	N	

	4.3 Were outcome assessors aware of the intervention received by study participants?			PN	
	4.4 If Y/PY/NI to 4.3: Could assessment of the outcome have been influenced by knowledge of intervention received?			NA	
	4.5 If Y/PY/NI to 4.4: Is it likely that assessment of the outcome was influenced by knowledge of intervention received?			NA	
	**Risk of bias judgement**			**Low**	
**Bias in selection of the reported result**	5.1 Were the data that produced this result analysed in accordance with a pre-specified analysis plan that was finalized before unblinded outcome data were available for analysis?			Y	
	5.2 ... multiple eligible outcome measurements (e.g. scales, definitions, time points) within the outcome domain?			PN	
	5.3 ... multiple eligible analyses of the data?			PN	
	**Risk of bias judgement**			**Low**	
**Overall bias**	**Risk of bias judgement**			**Low**	
**Unique ID**	26	**Study ID**	450	**Assessor**	KS TK
**Ref or Label**	Nathan_2008	**Aim**	assignment to intervention (the 'intention-to-treat' effect)		
**Experimental**	FF	**Comparator**	Placebo	**Source**	Journal article(s)
**Outcome**		**Results**		**Weight**	
**Domain**	**Signalling question**			**Response**	**Comments**

**Bias arising from the randomization process**	1.1 Was the allocation sequence random?	Y	
	1.2 Was the allocation sequence concealed until participants were enrolled and assigned to interventions?	Y	
	1.3 Did baseline differences between intervention groups sµggest a problem with the randomization process?	N	
	**Risk of bias judgement**	**Low**	
**Bias due to deviations from intended interventions**	2.1.Were participants aware of their assigned intervention during the trial?	N	
	2.2.Were carers and people delivering the interventions aware of participants' assigned intervention during the trial?	N	
	2.3. If Y/PY/NI to 2.1 or 2.2: Were there deviations from the intended intervention that arose because of the experimental context?	NA	
	2.4 If Y/PY to 2.3: Were these deviations likely to have affected the outcome?	NA	
	2.5. If Y/PY/NI to 2.4: Were these deviations from intended intervention balanced between groups?	NA	
	2.6 Was an appropriate analysis used to estimate the effect of assignment to intervention?	Y	
	2.7 If N/PN/NI to 2.6: Was there potential for a substantial impact (on the result) of the failure to analyse participants in the group to which they were randomized?	NA	
	**Risk of bias judgement**	**Low**	
**Bias due to missing outcome data**	3.1 Were data for this outcome available for all, or nearly all, participants randomized?	Y	
	3.2 If N/PN/NI to 3.1: Is there evidence that result was not biased by missing outcome data?	NA	
	3.3 If N/PN to 3.2: Could missingness in the outcome depend on its true value?	NA	

	3.4 If Y/PY/NI to 3.3: Is it likely that missingness in the outcome depended on its true value?	NA	
	**Risk of bias judgement**	**Low**	
**Bias in measurement of the outcome**	4.1 Was the method of measuring the outcome inappropriate?	N	
	4.2 Could measurement or ascertainment of the outcome have differed between intervention groups?	N	
	4.3 Were outcome assessors aware of the intervention received by study participants?	N	
	4.4 If Y/PY/NI to 4.3: Could assessment of the outcome have been influenced by knowledge of intervention received?	NA	
	4.5 If Y/PY/NI to 4.4: Is it likely that assessment of the outcome was influenced by knowledge of intervention received?	NA	
	**Risk of bias judgement**	**Low**	
**Bias in selection of the reported result**	5.1 Were the data that produced this result analysed in accordance with a pre-specified analysis plan that was finalized before unblinded outcome data were available for analysis?	Y	
	5.2 ... multiple eligible outcome measurements (e.g. scales, definitions, time points) within the outcome domain?	N	
	5.3 ... multiple eligible analyses of the data?	N	
	**Risk of bias judgement**	**Low**	
**Overall bias**	**Risk of bias judgement**	**Low**	

**Unique ID**	27	**Study ID**	840	**Assessor**	KS TK

**Ref or Label**	Vasar_2008	**Aim**	assignment to intervention (the 'intention-to-treat' effect)		
**Experimental**	FF	**Comparator**	Placebo	**Source**	Journal article(s)
**Outcome**		**Results**		**Weight**	

Domain	Signalling question	Response	Comments
**Bias arising from the randomization process**	1.1 Was the allocation sequence random?	PY	
	1.2 Was the allocation sequence concealed until participants were enrolled and assigned to interventions?	PY	
	1.3 Did baseline differences between intervention groups sµggest a problem with the randomization process?	N	
	**Risk of bias judgement**	**Low**	
**Bias due to deviations from intended interventions**	2.1.Were participants aware of their assigned intervention during the trial?	N	
	2.2.Were carers and people delivering the interventions aware of participants' assigned intervention during the trial?	N	
	2.3. If Y/PY/NI to 2.1 or 2.2: Were there deviations from the intended intervention that arose because of the experimental context?	NA	
	2.4 If Y/PY to 2.3: Were these deviations likely to have affected the outcome?	NA	
	2.5. If Y/PY/NI to 2.4: Were these deviations from intended intervention balanced between groups?	NA	
	2.6 Was an appropriate analysis used to estimate the effect of assignment to intervention?	Y	
	2.7 If N/PN/NI to 2.6: Was there potential for a substantial impact (on the result) of the failure to analyse participants in the group to which they were randomized?	NA	

	**Risk of bias judgement**	**Low**	
**Bias due to missing outcome data**	3.1 Were data for this outcome available for all, or nearly all, participants randomized?	PN	The drop out is large.
	3.2 If N/PN/NI to 3.1: Is there evidence that result was not biased by missing outcome data?	PN	
	3.3 If N/PN to 3.2: Could missingness in the outcome depend on its true value?	PY	
	3.4 If Y/PY/NI to 3.3: Is it likely that missingness in the outcome depended on its true value?	PY	
	**Risk of bias judgement**	**High**	
**Bias in measurement of the outcome**	4.1 Was the method of measuring the outcome inappropriate?	N	
	4.2 Could measurement or ascertainment of the outcome have differed between intervention groups?	N	
	4.3 Were outcome assessors aware of the intervention received by study participants?	N	
	4.4 If Y/PY/NI to 4.3: Could assessment of the outcome have been influenced by knowledge of intervention received?	NA	
	4.5 If Y/PY/NI to 4.4: Is it likely that assessment of the outcome was influenced by knowledge of intervention received?	NA	
	**Risk of bias judgement**	**Low**	
**Bias in selection of the reported result**	5.1 Were the data that produced this result analysed in accordance with a pre-specified analysis plan that was finalized before unblinded outcome data were available for analysis?	Y	
	5.2 ... multiple eligible outcome measurements (e.g. scales, definitions, time points) within the outcome domain?	N	
	5.3 ... multiple eligible analyses of the data?	N	

	**Risk of bias judgement**			**Low**	
**Overall bias**	**Risk of bias judgement**			**High**	Drop out from the study >20%
**Unique ID**	28	**Study ID**	576	**Assessor**	KS TK
**Ref or Label**	Meltzer_2007	**Aim**	assignment to intervention (the 'intention-to-treat' effect)		
**Experimental**	CIC	**Comparator**	Placebo	**Source**	Journal article(s)
**Outcome**		**Results**		**Weight**	
**Domain**	**Signalling question**			**Response**	**Comments**
**Bias arising from the randomization process**	1.1 Was the allocation sequence random?			PY	
	1.2 Was the allocation sequence concealed until participants were enrolled and assigned to interventions?			PY	
	1.3 Did baseline differences between intervention groups sμggest a problem with the randomization process?			N	
	**Risk of bias judgement**			**Low**	
**Bias due to deviations from intended interventions**	2.1.Were participants aware of their assigned intervention during the trial?			PN	
	2.2.Were carers and people delivering the interventions aware of participants' assigned intervention during the trial?			PN	
	2.3. If Y/PY/NI to 2.1 or 2.2: Were there deviations from the intended intervention that arose because of the experimental context?			NA	

	2.4 If Y/PY to 2.3: Were these deviations likely to have affected the outcome?	NA	
	2.5. If Y/PY/NI to 2.4: Were these deviations from intended intervention balanced between groups?	NA	
	2.6 Was an appropriate analysis used to estimate the effect of assignment to intervention?	Y	
	2.7 If N/PN/NI to 2.6: Was there potential for a substantial impact (on the result) of the failure to analyse participants in the group to which they were randomized?	NA	
	**Risk of bias judgement**	**Low**	
**Bias due to missing outcome data**	3.1 Were data for this outcome available for all, or nearly all, participants randomized?	Y	
	3.2 If N/PN/NI to 3.1: Is there evidence that result was not biased by missing outcome data?	NA	
	3.3 If N/PN to 3.2: Could missingness in the outcome depend on its true value?	NA	
	3.4 If Y/PY/NI to 3.3: Is it likely that missingness in the outcome depended on its true value?	NA	
	**Risk of bias judgement**	**Low**	
**Bias in measurement of the outcome**	4.1 Was the method of measuring the outcome inappropriate?	N	
	4.2 Could measurement or ascertainment of the outcome have differed between intervention groups?	N	
	4.3 Were outcome assessors aware of the intervention received by study participants?	N	
	4.4 If Y/PY/NI to 4.3: Could assessment of the outcome have been influenced by knowledge of intervention received?	NA	
	4.5 If Y/PY/NI to 4.4: Is it likely that assessment of the outcome was influenced by knowledge of intervention received?	NA	

	**Risk of bias judgement**			**Low**	
**Bias in selection of the reported result**	5.1 Were the data that produced this result analysed in accordance with a pre-specified analysis plan that was finalized before unblinded outcome data were available for analysis?			Y	
	5.2 ... multiple eligible outcome measurements (e.g. scales, definitions, time points) within the outcome domain?			N	
	5.3 ... multiple eligible analyses of the data?			N	
	**Risk of bias judgement**			**Low**	
**Overall bias**	**Risk of bias judgement**			**Low**	
**Unique ID**	29	**Study ID**	1065	**Assessor**	KS TK
**Ref or Label**	Chervinsky_2007	**Aim**	assignment to intervention (the 'intention-to-treat' effect)		
**Experimental**	CIC	**Comparator**	Placebo	**Source**	Journal article(s)
**Outcome**		**Results**		**Weight**	
**Domain**	**Signalling question**			**Response**	**Comments**
**Bias arising from the randomization process**	1.1 Was the allocation sequence random?			PY	
	1.2 Was the allocation sequence concealed until participants were enrolled and assigned to interventions?			PY	
	1.3 Did baseline differences between intervention groups sµggest a problem with the randomization process?			N	

	**Risk of bias judgement**	**Low**	
**Bias due to deviations from intended interventions**	2.1.Were participants aware of their assigned intervention during the trial?	N	
	2.2.Were carers and people delivering the interventions aware of participants' assigned intervention during the trial?	N	
	2.3. If Y/PY/NI to 2.1 or 2.2: Were there deviations from the intended intervention that arose because of the experimental context?	NA	
	2.4 If Y/PY to 2.3: Were these deviations likely to have affected the outcome?	NA	
	2.5. If Y/PY/NI to 2.4: Were these deviations from intended intervention balanced between groups?	NA	
	2.6 Was an appropriate analysis used to estimate the effect of assignment to intervention?	Y	
	2.7 If N/PN/NI to 2.6: Was there potential for a substantial impact (on the result) of the failure to analyse participants in the group to which they were randomized?	NA	
	**Risk of bias judgement**	**Low**	
**Bias due to missing outcome data**	3.1 Were data for this outcome available for all, or nearly all, participants randomized?	PN	The drop out is large.
	3.2 If N/PN/NI to 3.1: Is there evidence that result was not biased by missing outcome data?	PN	
	3.3 If N/PN to 3.2: Could missingness in the outcome depend on its true value?	PY	
	3.4 If Y/PY/NI to 3.3: Is it likely that missingness in the outcome depended on its true value?	PY	
	**Risk of bias judgement**	**High**	
**Bias in measurement of the outcome**	4.1 Was the method of measuring the outcome inappropriate?	N	

	4.2 Could measurement or ascertainment of the outcome have differed between intervention groups?	N	
	4.3 Were outcome assessors aware of the intervention received by study participants?	N	
	4.4 If Y/PY/NI to 4.3: Could assessment of the outcome have been influenced by knowledge of intervention received?	NA	
	4.5 If Y/PY/NI to 4.4: Is it likely that assessment of the outcome was influenced by knowledge of intervention received?	NA	
	**Risk of bias judgement**	**Low**	
**Bias in selection of the reported result**	5.1 Were the data that produced this result analysed in accordance with a pre-specified analysis plan that was finalized before unblinded outcome data were available for analysis?	Y	
	5.2 ... multiple eligible outcome measurements (e.g. scales, definitions, time points) within the outcome domain?	N	
	5.3 ... multiple eligible analyses of the data?	N	
	**Risk of bias judgement**	**Low**	
**Overall bias**	**Risk of bias judgement**	**High**	Drop out from the study >20%

**Unique ID**	30	**Study ID**	9989	**Assessor**	KS TK
**Ref or Label**	Rosenblut_2007	**Aim**	assignment to intervention (the 'intention-to-treat' effect)		
**Experimental**	FF	**Comparator**	Placebo	**Source**	Journal article(s)
**Outcome**		**Results**		**Weight**	

Domain	Signalling question	Response	Comments
**Bias arising from the randomization process**	1.1 Was the allocation sequence random?	Y	
	1.2 Was the allocation sequence concealed until participants were enrolled and assigned to interventions?	Y	
	1.3 Did baseline differences between intervention groups sμggest a problem with the randomization process?	PN	
	**Risk of bias judgement**	**Low**	
**Bias due to deviations from intended interventions**	2.1.Were participants aware of their assigned intervention during the trial?	N	
	2.2.Were carers and people delivering the interventions aware of participants' assigned intervention during the trial?	N	
	2.3. If Y/PY/NI to 2.1 or 2.2: Were there deviations from the intended intervention that arose because of the experimental context?	NA	
	2.4 If Y/PY to 2.3: Were these deviations likely to have affected the outcome?	NA	
	2.5. If Y/PY/NI to 2.4: Were these deviations from intended intervention balanced between groups?	NA	
	2.6 Was an appropriate analysis used to estimate the effect of assignment to intervention?	Y	
	2.7 If N/PN/NI to 2.6: Was there potential for a substantial impact (on the result) of the failure to analyse participants in the group to which they were randomized?	NA	
	**Risk of bias judgement**	**Low**	
**Bias due to missing outcome data**	3.1 Were data for this outcome available for all, or nearly all, participants randomized?	PN	The drop out is large.
	3.2 If N/PN/NI to 3.1: Is there evidence that result was not biased by missing outcome data?	PN	

	3.3 If N/PN to 3.2: Could missingness in the outcome depend on its true value?	PY	
	3.4 If Y/PY/NI to 3.3: Is it likely that missingness in the outcome depended on its true value?	PY	
	**Risk of bias judgement**	**High**	
**Bias in measurement of the outcome**	4.1 Was the method of measuring the outcome inappropriate?	N	
	4.2 Could measurement or ascertainment of the outcome have differed between intervention groups?	N	
	4.3 Were outcome assessors aware of the intervention received by study participants?	N	
	4.4 If Y/PY/NI to 4.3: Could assessment of the outcome have been influenced by knowledge of intervention received?	NA	
	4.5 If Y/PY/NI to 4.4: Is it likely that assessment of the outcome was influenced by knowledge of intervention received?	NA	
	**Risk of bias judgement**	**Low**	
**Bias in selection of the reported result**	5.1 Were the data that produced this result analysed in accordance with a pre-specified analysis plan that was finalized before unblinded outcome data were available for analysis?	Y	
	5.2 ... multiple eligible outcome measurements (e.g. scales, definitions, time points) within the outcome domain?	PN	
	5.3 ... multiple eligible analyses of the data?	PN	
	**Risk of bias judgement**	**Low**	
**Overall bias**	**Risk of bias judgement**	**High**	Drop out from the study >20%

**Unique ID**	31	**Study ID**	9988	**Assessor**	KS TK
**Ref or Label**	Tai_2003	**Aim**	assignment to intervention (the 'intention-to-treat' effect)		
**Experimental**	FP	**Comparator**	BUD	**Source**	Journal article(s)
**Outcome**		**Results**		**Weight**	
**Domain**	**Signalling question**			**Response**	**Comments**
**Bias arising from the randomization process**	1.1 Was the allocation sequence random?			Y	
	1.2 Was the allocation sequence concealed until participants were enrolled and assigned to interventions?			Y	
	1.3 Did baseline differences between intervention groups sμggest a problem with the randomization process?			N	
	**Risk of bias judgement**			**Low**	
**Bias due to deviations from intended interventions**	2.1.Were participants aware of their assigned intervention during the trial?			N	
	2.2.Were carers and people delivering the interventions aware of participants' assigned intervention during the trial?			N	
	2.3. If Y/PY/NI to 2.1 or 2.2: Were there deviations from the intended intervention that arose because of the experimental context?			NA	
	2.4 If Y/PY to 2.3: Were these deviations likely to have affected the outcome?			NA	
	2.5. If Y/PY/NI to 2.4: Were these deviations from intended intervention balanced between groups?			NA	
	2.6 Was an appropriate analysis used to estimate the effect of assignment to intervention?			PY	

	2.7 If N/PN/NI to 2.6: Was there potential for a substantial impact (on the result) of the failure to analyse participants in the group to which they were randomized?	NA	
	**Risk of bias judgement**	**Low**	
**Bias due to missing outcome data**	3.1 Were data for this outcome available for all, or nearly all, participants randomized?	Y	
	3.2 If N/PN/NI to 3.1: Is there evidence that result was not biased by missing outcome data?	NA	
	3.3 If N/PN to 3.2: Could missingness in the outcome depend on its true value?	NA	
	3.4 If Y/PY/NI to 3.3: Is it likely that missingness in the outcome depended on its true value?	NA	
	**Risk of bias judgement**	**Low**	
**Bias in measurement of the outcome**	4.1 Was the method of measuring the outcome inappropriate?	N	
	4.2 Could measurement or ascertainment of the outcome have differed between intervention groups?	NI	
	4.3 Were outcome assessors aware of the intervention received by study participants?	PN	
	4.4 If Y/PY/NI to 4.3: Could assessment of the outcome have been influenced by knowledge of intervention received?	NA	
	4.5 If Y/PY/NI to 4.4: Is it likely that assessment of the outcome was influenced by knowledge of intervention received?	NA	
	**Risk of bias judgement**	**Some concerns**	
**Bias in selection of the reported result**	5.1 Were the data that produced this result analysed in accordance with a pre-specified analysis plan that was finalized before unblinded outcome data were available for analysis?	Y	
	5.2 ... multiple eligible outcome measurements (e.g. scales, definitions, time points) within the outcome domain?	PN	

	5.3 ... multiple eligible analyses of the data?			PN	
	**Risk of bias judgement**			**Low**	
**Overall bias**	**Risk of bias judgement**			**Some concerns**	
**Unique ID**	32	**Study ID**	130	**Assessor**	KS TK
**Ref or Label**	Fokkens_2002	**Aim**	assignment to intervention (the 'intention-to-treat' effect)		
**Experimental**	BUD	**Comparator**	Placebo	**Source**	Journal article(s)
**Outcome**		**Results**		**Weight**	
**Domain**	**Signalling question**			**Response**	**Comments**
**Bias arising from the randomization process**	1.1 Was the allocation sequence random?			Y	
	1.2 Was the allocation sequence concealed until participants were enrolled and assigned to interventions?			Y	
	1.3 Did baseline differences between intervention groups sμggest a problem with the randomization process?			N	
	**Risk of bias judgement**			**Low**	
**Bias due to deviations from intended interventions**	2.1.Were participants aware of their assigned intervention during the trial?			N	
	2.2.Were carers and people delivering the interventions aware of participants' assigned intervention during the trial?			N	

	2.3. If Y/PY/NI to 2.1 or 2.2: Were there deviations from the intended intervention that arose because of the experimental context?	NA	
	2.4 If Y/PY to 2.3: Were these deviations likely to have affected the outcome?	NA	
	2.5. If Y/PY/NI to 2.4: Were these deviations from intended intervention balanced between groups?	NA	
	2.6 Was an appropriate analysis used to estimate the effect of assignment to intervention?	Y	
	2.7 If N/PN/NI to 2.6: Was there potential for a substantial impact (on the result) of the failure to analyse participants in the group to which they were randomized?	NA	
	**Risk of bias judgement**	**Low**	
**Bias due to missing outcome data**	3.1 Were data for this outcome available for all, or nearly all, participants randomized?	Y	
	3.2 If N/PN/NI to 3.1: Is there evidence that result was not biased by missing outcome data?	NA	
	3.3 If N/PN to 3.2: Could missingness in the outcome depend on its true value?	NA	
	3.4 If Y/PY/NI to 3.3: Is it likely that missingness in the outcome depended on its true value?	NA	
	**Risk of bias judgement**	**Low**	
**Bias in measurement of the outcome**	4.1 Was the method of measuring the outcome inappropriate?	N	
	4.2 Could measurement or ascertainment of the outcome have differed between intervention groups?	N	
	4.3 Were outcome assessors aware of the intervention received by study participants?	N	
	4.4 If Y/PY/NI to 4.3: Could assessment of the outcome have been influenced by knowledge of intervention received?	NA	

	4.5 If Y/PY/NI to 4.4: Is it likely that assessment of the outcome was influenced by knowledge of intervention received?			NA	
	**Risk of bias judgement**			**Low**	
**Bias in selection of the reported result**	5.1 Were the data that produced this result analysed in accordance with a pre-specified analysis plan that was finalized before unblinded outcome data were available for analysis?			Y	
	5.2 ... multiple eligible outcome measurements (e.g. scales, definitions, time points) within the outcome domain?			N	
	5.3 ... multiple eligible analyses of the data?			N	
	**Risk of bias judgement**			**Low**	
**Overall bias**	**Risk of bias judgement**			**Low**	
**Unique ID**	33	**Study ID**	1790	**Assessor**	KS TK
**Ref or Label**	Kobayashi_1995	**Aim**	assignment to intervention (the 'intention-to-treat' effect)		
**Experimental**	TA	**Comparator**	Placebo	**Source**	Journal article(s)
**Outcome**		**Results**		**Weight**	
**Domain**	**Signalling question**			**Response**	**Comments**
**Bias arising from the randomization process**	1.1 Was the allocation sequence random?			PY	
	1.2 Was the allocation sequence concealed until participants were enrolled and assigned to interventions?			PY	

	1.3 Did baseline differences between intervention groups sμggest a problem with the randomization process?	N	
	**Risk of bias judgement**	**Low**	
**Bias due to deviations from intended interventions**	2.1.Were participants aware of their assigned intervention during the trial?	N	
	2.2.Were carers and people delivering the interventions aware of participants' assigned intervention during the trial?	N	
	2.3. If Y/PY/NI to 2.1 or 2.2: Were there deviations from the intended intervention that arose because of the experimental context?	NA	
	2.4 If Y/PY to 2.3: Were these deviations likely to have affected the outcome?	NA	
	2.5. If Y/PY/NI to 2.4: Were these deviations from intended intervention balanced between groups?	NA	
	2.6 Was an appropriate analysis used to estimate the effect of assignment to intervention?	Y	
	2.7 If N/PN/NI to 2.6: Was there potential for a substantial impact (on the result) of the failure to analyse participants in the group to which they were randomized?	NA	
	**Risk of bias judgement**	**Low**	
**Bias due to missing outcome data**	3.1 Were data for this outcome available for all, or nearly all, participants randomized?	Y	
	3.2 If N/PN/NI to 3.1: Is there evidence that result was not biased by missing outcome data?	NA	
	3.3 If N/PN to 3.2: Could missingness in the outcome depend on its true value?	NA	
	3.4 If Y/PY/NI to 3.3: Is it likely that missingness in the outcome depended on its true value?	NA	
	**Risk of bias judgement**	**Low**	

**Bias in measurement of the outcome**	4.1 Was the method of measuring the outcome inappropriate?	N	
	4.2 Could measurement or ascertainment of the outcome have differed between intervention groups?	N	
	4.3 Were outcome assessors aware of the intervention received by study participants?	N	
	4.4 If Y/PY/NI to 4.3: Could assessment of the outcome have been influenced by knowledge of intervention received?	NA	
	4.5 If Y/PY/NI to 4.4: Is it likely that assessment of the outcome was influenced by knowledge of intervention received?	NA	
	**Risk of bias judgement**	**Low**	
**Bias in selection of the reported result**	5.1 Were the data that produced this result analysed in accordance with a pre-specified analysis plan that was finalized before unblinded outcome data were available for analysis?	Y	
	5.2 ... multiple eligible outcome measurements (e.g. scales, definitions, time points) within the outcome domain?	N	
	5.3 ... multiple eligible analyses of the data?	N	
	**Risk of bias judgement**	**Low**	
**Overall bias**	**Risk of bias judgement**	**Low**	

Supplementary Table S6. League table of standardized mean difference for total ocular symptom score changes from baseline in patients with seasonal allergic rhinitis

**FF**			**-0.32 (-0.46,-0.19)**
-0.03 (-0.26,0.20)	**MF**		-0.7 (-1.87,0.46)
-0.08 (-0.28,0.13)	-0.05 (-0.29,0.20)	**FP**	**-0.24 (-0.40,-0.09)**
**-0.32 (-0.45,-0.19)**	**-0.29 (-0.48,-0.10)**	**-0.24 (-0.40,-0.09)**	**PLAC**

FF, fluticasone furoate; FP, fluticasone propionate; MF, mometasone furoate; PLAC, Placebo.
Bold values indicate statistical significance.
The numbers in the upper-right portion of the league table represent the standardized mean difference (SMD) of total ocular symptom score (TOSS) changes from baseline in seasonal allergic rhinitis (SAR) from the pooling of direct evidence from pairwise meta-analyses. The lower-left portion of the league table represents the SMDs of TOSS changes from baseline in SAR from network meta-analysis (NMA) or indirect evidence. Treatments are arranged in order of the mean ranking from NMA from the best (left) to the worst (right). For the direct evidence, negative SMDs indicate that the treatment specified in the row is superior to that specified in the column. For the indirect evidence, negative SMDs indicate that the treatment specified in the column is superior to that specified in the row.

Supplementary Table S7. Sensitivity analyses according to the outcomes measured

(A) TNSS changes from baseline in SAR

Treatment	Primary analysis (main findings)	SUCRA rank	Excluding studies with imputation of missing SD values	SUCRA rank	Excluding studies with significant risk of bias	SUCRA rank	Excluding studies published before 2000	SUCRA rank	Excluding studies with small sample size	SUCRA rank
**Number of studies**	12 (15 treatment pairs)		11		11		11		11	
**MF**	**-0.47 (-0.63,-0.31)**	2.7	**-0.45 (-0.66,0.24)**	2.9	**-0.46 (-0.62,-0.30)**	2.8	**-0.49 (-0.67,-0.31)**	2.5	**-0.46 (-0.62,-0.30)**	2.8
**FF**	**-0.46 (-0.59,-0.33)**	2.8	**-0.46 (-0.60,-0.33)**	2.8	**-0.46 (-0.59,-0.33)**	2.8	**-0.46 (-0.59,-0.33)**	2.9	**-0.46 (-0.59,-0.33)**	2.8
**CIC**	**-0.44 (-0.75,-0.13)**	3	**-0.44 (-0.78,-0.10)**	3	**-0.44 (-0.75,-0.13)**	3	**-0.44 (-0.76,-0.12)**	3.1	**-0.44 (-0.75,-0.13)**	3
**FP**	**-0.42 (-0.67,-0.17)**	3.2	**-0.42 (-0.69,-0.15)**	3.2	**-0.42 (-0.67,-0.17)**	3.2	**-0.42 (-0.68,-0.16)**	3.3	**-0.42 (-0.67,-0.17)**	3.2
**TAA**	**-0.41 (-0.81,-0.00)**	3.3	-0.41 (-0.85,0.03)	3.2	**-0.41 (-0.81,-0.00)**	3.3	-0.41 (-0.83,0.01)	3.3	**-0.41 (-0.81,-0.00)**	3.3
**Placebo**	Reference	6	Reference	6	Reference	6	Reference	6	Reference	6
**Global inconsistency χ2 (*P* value)**	0.29 (0.5890)		0.23 (0.6337)		0.29 (0.5889)		0.26 (0.6072)		0.29 (0.5889)	

(B) Leave-one-out sensitivity analysis of TNSS changes from baseline in SAR

Treatment	Primary analysis (main findings)	Leave-one-out sensitivity analysis							
		Exclude study 1	Exclude study 2	Exclude study 3	Exclude study 4	Exclude study 5	Exclude study 6	Exclude study 7	Exclude study 8
**MF**	**-0.47 (-0.63,-0.31)**	**-0.49 (-0.63,-0.35)**	**-0.47 (-0.63,-0.31)**	**-0.47 (-0.63,-0.31)**	**-0.45 (-0.66,-0.24)**	**-0.45 (-0.65,-0.24)**	**-0.47 (-0.64,-0.30)**	**-0.50 (-0.69,-0.31)**	**-0.49 (-0.67,-0.31)**

**FF**	**-0.46 (-0.59,-0.33)**	**-0.47 (-0.63,-0.31)**	**-0.46 (-0.59,-0.33)**	**-0.44 (-0.58,-0.30)**	**-0.46 (-0.60,-0.33)**	**-0.46 (-0.60,-0.33)**	**-0.47 (-0.62,-0.33)**	**-0.46 (-0.59,-0.33)**	**-0.46 (-0.59,-0.33)**
**CIC**	**-0.44 (-0.75,-0.13)**	**-0.44 (-0.76,-0.12)**	NA	**-0.44 (-0.76,-0.12)**	**-0.44 (-0.78,-0.10)**	**-0.44 (-0.78,-0.10)**	**-0.44 (-0.78,-0.10)**	**-0.44 (-0.77,-0.11)**	**-0.44 (-0.76,-0.12)**
**FP**	**-0.42 (-0.67,-0.17)**	**-0.44 (-0.69,-0.18)**	**-0.42 (-0.67,-0.17)**	**-0.41 (-0.67,-0.15)**	**-0.42 (-0.69,-0.15)**	**-0.42 (-0.69,-0.15)**	**-0.43 (-0.70,-0.16)**	**-0.42 (-0.68,-0.16)**	**-0.42 (-0.68,-0.16)**
**TAA**	**-0.41 (-0.81,-0.00)**	**-0.43 (-0.84,-0.01)**	**-0.41 (-0.81,-0.00)**	-0.40 (-0.82,0.02)	-0.41 (-0.85,0.03)	-0.41 (-0.85,0.03)	-0.42 (-0.86,0.02)	-0.41 (-0.84,0.01)	-0.41 (-0.83,0.01)
**PLAC**	Reference	Reference	Reference	Reference	Reference	Reference	Reference	Reference	Reference
**Global inconsistency χ2 (*P* value)**	0.29 (0.5890)	0.16 (0.6859)	0.29 (0.5889)	0.35 (0.5558)	0.23 (0.6337)	0.23 (0.6304)	0.19 (0.6616)	0.25 (0.6180)	0.26 (0.6072)

(B) Leave-one-out sensitivity analysis of TNSS changes from baseline in SAR (continued)

**Treatment**	**Primary analysis (main findings)**	**Leave-one-out sensitivity analysis**						
		Exclude study 9	Exclude study 10	Exclude study 11 (treatment pair 1)	Exclude study 11 (treatment pair 2)	Exclude study 11 (treatment pair 3)	Exclude study 12 (treatment pair 1)	Exclude study 12 (treatment pair 2)
**MF**	**-0.47 (-0.63,-0.31)**	**-0.47 (-0.63,-0.31)**	**-0.46 (-0.62,-0.30)**	**-0.47 (-0.63,-0.30)**	**-0.47 (-0.63,-0.30)**	**-0.48 (-0.58,-0.38)**	**-0.47 (-0.64,-0.30)**	**-0.47 (-0.63,-0.30)**
**FF**	**-0.46 (-0.59,-0.33)**	**-0.46 (-0.59,-0.33)**	**-0.46 (-0.59,-0.33)**	**-0.45 (-0.59,-0.31)**	**-0.45 (-0.59,-0.31)**	**-0.39 (-0.48,-0.30)**	**-0.49 (-0.64,-0.34)**	**-0.49 (-0.63,-0.34)**
**CIC**	**-0.44 (-0.75,-0.13)**	**-0.44 (-0.75,-0.13)**	**-0.44 (-0.75,-0.13)**	**-0.44 (-0.77,-0.11)**	**-0.44 (-0.77,-0.11)**	**-0.44 (-0.66,-0.22)**	**-0.44 (-0.77,-0.11)**	**-0.44 (-0.77,-0.11)**
**FP**	**-0.42 (-0.67,-0.17)**	**-0.42 (-0.67,-0.17)**	**-0.42 (-0.67,-0.17)**	**-0.49 (-0.87,-0.12)**	-0.35 (-0.71,0.01)	**-0.37 (-0.56,-0.18)**	**-0.44 (-0.70,-0.17)**	**-0.44 (-0.70,-0.17)**
**TAA**	**-0.41 (-0.81,-0.00)**	NA	**-0.41 (-0.81,-0.00)**	-0.49 (-0.99,0.01)	-0.34 (-0.83,0.15)	**-0.36 (-0.66,-0.07)**	-0.43 (-0.85,0.00)	**-0.43 (-0.85,-0.00)**
**PLAC**	Reference	Reference	Reference	Reference	Reference	Reference	Reference	Reference
**Global inconsistency χ2 (*P* value)**	0.29 (0.5890)	0.29 (0.5890)	0.29 (0.5889)	NA	NA	1.27 (0.2600)	0.16 (0.6923)	0.16 (0.6900)

(C) TNSS changes from baseline in PAR

Treatment	Primary analysis (main findings)	SUCRA rank	Excluding studies in children	SUCRA rank	Excluding studies with significant risk of bias	SUCRA rank	Excluding studies published before 2000	SUCRA rank	Excluding studies with small sample size	SUCRA rank
**Number of studies**	12		9		10		11		10	
**BUD**	**-0.43 (-0.75,-0.11)**	2.4	-0.34 (-1.27,0.60)	4	**-0.43 (-0.73,-0.14)**	2	**-0.42 (-0.72,-0.12)**	2	**-0.47 (-0.81,-0.12)**	2
**FF**	**-0.36 (-0.53,-0.19)**	3	**-0.36 (-0.52,-0.21)**	3.9	**-0.26 (-0.45,-0.07)**	4	**-0.36 (-0.51,-0.21)**	2.5	**-0.36 (-0.54,-0.19)**	2.9
**TAA**	**-0.32 (-0.54,-0.10)**	3.7	**-0.49 (-0.83,-0.16)**	2.8	**-0.30 (-0.52,-0.09)**	3.4	-0.22 (-0.44,0.01)	4.5	**-0.30 (-0.53,-0.08)**	3.7
**CIC**	**-0.29 (-0.48,-0.11)**	4	**-0.29 (-0.45,-0.14)**	4.6	**-0.27 (-0.50,-0.04)**	3.9	**-0.29 (-0.45,-0.14)**	3.5	**-0.29 (-0.48,-0.11)**	3.8
**FP**	-0.28 (-0.64,0.08)	4	-0.43 (-0.86,0.01)	3.5	-0.27 (-0.61,0.08)	4	-0.19 (-0.53,0.16)	4.8	-0.23 (-0.62,0.16)	4.4
**MF**	**-0.28 (-0.55,-0.01)**	4.1	-0.66 (-1.48,0.16)	2.5	**-0.27 (-0.52,-0.03)**	3.9	**-0.27 (-0.51,-0.03)**	3.8	-0.24 (-0.53,0.05)	4.5
**PLAC**	Reference	6.9	Reference	6.7	Reference	6.9	Reference	6.8	Reference	6.8
**Global inconsistency χ2 (*P* value)**	0.41 (0.5207)		NA		0.47 (0.4915)		0.79 (0.3751)		NA	

(D) Leave-one-out sensitivity analysis of TNSS changes from baseline in PAR

Treatment	Primary analysis (main findings)	Leave-one-out sensitivity analysis					
		Exclude study 1	Exclude study 2	Exclude study 3	Exclude study 4	Exclude study 5	Exclude study 6
**BUD**	**-0.43 (-0.75,-0.11)**	-0.14 (-1.07,0.78)	**-0.43 (-0.76,-0.09)**	**-0.43 (-0.75,-0.11)**	**-0.42 (-0.78,-0.06)**	**-0.43 (-0.76,-0.10)**	**-0.43 (-0.70,-0.17)**
**FF**	**-0.36 (-0.53,-0.19)**	**-0.36 (-0.54,-0.19)**	**-0.41 (-0.64,-0.18)**	**-0.36 (-0.54,-0.19)**	**-0.36 (-0.57,-0.16)**	**-0.42 (-0.64,-0.20)**	**-0.26 (-0.42,-0.10)**
**TAA**	**-0.32 (-0.54,-0.10)**	**-0.30 (-0.53,-0.08)**	**-0.32 (-0.56,-0.09)**	**-0.32 (-0.54,-0.10)**	**-0.33 (-0.58,-0.08)**	**-0.32 (-0.55,-0.09)**	**-0.28 (-0.44,-0.13)**
**CIC**	**-0.29 (-0.48,-0.11)**	**-0.29 (-0.48,-0.11)**	**-0.29 (-0.50,-0.09)**	**-0.29 (-0.48,-0.11)**	-0.31 (-0.63,0.00)	**-0.29 (-0.49,-0.10)**	**-0.30 (-0.42,-0.17)**

**FP**	-0.28 (-0.64,0.08)	-0.23 (-0.62,0.16)	-0.29 (-0.67,0.09)	-0.28 (-0.64,0.08)	-0.30 (-0.71,0.11)	-0.29 (-0.66,0.09)	-0.24 (-0.52,0.03)
**MF**	**-0.28 (-0.55,-0.01)**	**-0.28 (-0.56,-0.01)**	-0.29 (-0.58,0.01)	-0.66 (-1.49,0.17)	-0.30 (-0.62,0.02)	**-0.28 (-0.57,-0.00)**	**-0.26 (-0.46,-0.07)**
**PLAC**	Reference	Reference	Reference	Reference	Reference	Reference	Reference
**Global inconsistency χ2 (*P* value)**	0.41 (0.5207)	NA	0.38 (0.5402)	0.41 (0.5216)	0.34 (0.5624)	0.39 (0.5297)	0.57 (0.4484)

(D) Leave-one-out sensitivity analysis of TNSS changes from baseline in PAR (continued)

**Treatment**	**Primary analysis (main findings)**	**Leave-one-out sensitivity analysis**					
		Exclude study 7	Exclude study 8	Exclude study 9	Exclude study 10	Exclude study 11	Exclude study 12
**BUD**	**-0.43 (-0.75,-0.11)**	**-0.43 (-0.75,-0.11)**	**-0.42 (-0.78,-0.06)**	**-0.42 (-0.72,-0.12)**	**-0.47 (-0.81,-0.12)**	**-0.46 (-0.75,-0.17)**	**-0.47 (-0.81,-0.12)**
**FF**	**-0.36 (-0.53,-0.19)**	**-0.36 (-0.54,-0.19)**	**-0.36 (-0.57,-0.16)**	**-0.36 (-0.51,-0.21)**	**-0.36 (-0.54,-0.19)**	**-0.36 (-0.51,-0.21)**	**-0.36 (-0.54,-0.19)**
**TAA**	**-0.32 (-0.54,-0.10)**	**-0.32 (-0.54,-0.10)**	**-0.33 (-0.58,-0.08)**	-0.22 (-0.44,0.01)	**-0.30 (-0.53,-0.08)**	**-0.51 (-0.81,-0.20)**	**-0.30 (-0.53,-0.08)**
**CIC**	**-0.29 (-0.48,-0.11)**	**-0.29 (-0.48,-0.11)**	-0.27 (-0.60,0.05)	**-0.29 (-0.45,-0.14)**	**-0.29 (-0.48,-0.11)**	**-0.29 (-0.44,-0.15)**	**-0.29 (-0.48,-0.11)**
**FP**	**-0.28 (-0.64,0.08)**	-0.28 (-0.64,0.08)	-0.30 (-0.71,0.11)	-0.19 (-0.53,0.16)	-0.56 (-1.46,0.35)	**-0.45 (-0.83,-0.07)**	-0.23 (-0.62,0.16)
**MF**	**-0.28 (-0.55,-0.01)**	-0.24 (-0.52,0.04)	-0.30 (-0.62,0.02)	**-0.27 (-0.51,-0.03)**	**-0.28 (-0.56,-0.01)**	**-0.27 (-0.50,-0.04)**	**-0.28 (-0.56,-0.01)**
**PLAC**	Reference	Reference	Reference	Reference	Reference	Reference	Reference
**Global inconsistency χ2 (*P* value)**	0.41 (0.5207)	0.41 (0.5216)	0.34 (0.5624)	0.79 (0.3751)	NA	0.07 (0.7947)	NA

(E) TOSS changes from baseline in SAR

Treatment	Primary analysis (main findings)	SUCRA rank	Excluding studies with small sample size	SUCRA rank
**Number of studies**	6		5	
**FF**	**-0.32 (-0.45,-0.19)**	1.6	**-0.32 (-0.45,-0.19)**	1.5
**MF**	**-0.29 (-0.48,-0.10)**	1.9	**-0.27 (-0.46,-0.08)**	2.1
**FP**	**-0.24 (-0.40,-0.09)**	2.5	**-0.24 (-0.40,-0.09)**	2.4
**PLAC**	Reference	4	Reference	4
**Global inconsistency χ2 (*P* value)**	NA		NA	

(F) Acceptability in SAR

Treatment	Primary analysis (main findings)	SUCRA rank	Analysis with risk difference model (no imputation)	SUCRA rank	Excluding studies published before 2000	SUCRA rank	Excluding studies with small sample size	SUCRA rank
**Number of studies**	10 (11 treatment pairs)		7		6		6	
**FF**	0.60 (0.27,1.32)	2.5	**-0.39 (-0.49,-0.29)**	2.5	0.58 (0.22,1.50)	1.8	0.58 (0.22,1.50)	1.8
**FP**	0.57 (0.12,2.67)	2.5	NA	NA	NA	NA	NA	NA
**TAA**	0.74 (0.10,5.39)	3.3	NA	NA	NA	NA	NA	NA
**MF**	0.89 (0.25,3.20)	3.6	**-0.46 (-0.58,-0.33)**	1.6	0.66 (0.10,4.12)	2.1	0.66 (0.10,4.12)	2.1
**CIC**	1.56 (0.41,6.00)	4.9	**-0.44 (-0.66,-0.22)**	1.9	1.56 (0.29,8.49)	3.2	1.56 (0.29,8.49)	3.2
**PLAC**	Reference	4.1	Reference	4	Reference	2.9	Reference	2.9
**Global inconsistency χ2 (*P* value)**	NA		NA		NA		NA	

(G) Acceptability in PAR

Treatment	Primary analysis (main findings)	SUCRA rank	Analysis with risk difference model (no imputation)	SUCRA rank	Excluding studies with significant risk of bias	SUCRA rank	Excluding studies published before 2000	SUCRA rank	Excluding studies with small sample size	SUCRA rank
**Number of studies**	11		10		8		10		9	
**FF**	0.90 (0.67,1.20)	2.9	**-0.41 (-0.64,-0.17)**	2.3	0.97 (0.33,2.83)	3.4	0.90 (0.67,1.20)	3	0.90 (0.67,1.20)	2.6
**TAA**	0.92 (0.51,1.67)	3.3	**-0.31 (-0.55,-0.06)**	3.4	0.92 (0.51,1.67)	3.2	0.91 (0.48,1.71)	3.3	0.92 (0.51,1.67)	3
**CIC**	0.95 (0.71,1.28)	3.5	**-0.29 (-0.50,-0.09)**	3.6	1.05 (0.62,1.79)	3.8	0.95 (0.71,1.28)	3.5	0.95 (0.71,1.28)	3.1
**MF**	1.09 (0.40,2.98)	4	-0.29 (-0.59,0.01)	3.7	1.09 (0.40,2.98)	3.9	1.09 (0.40,2.98)	4	1.16 (0.41,3.25)	3.9
**BUD**	3.94 (0.04,409.94)	4.9	-0.15 (-1.10,0.79)	4.3	3.94 (0.04,409.94)	4.9	3.87 (0.04,405.89)	4.9	NA	NA
**FP**	2.77 (0.26,29.10)	5.4	-0.24 (-0.66,0.18)	4.1	2.77 (0.26,29.10)	5.4	2.73 (0.26,28.97)	5.4	2.77 (0.26,29.10)	4.9
**PLAC**	Reference	4	Reference	6.5	Reference	3.5	Reference	4	Reference	3.6
**Global inconsistency χ2 (*P* value)**	NA		NA		NA		NA		NA	

BDP, beclomethasone dipropionate; BUD, budesonide; CIC, ciclesonide; FF, fluticasone furoate; FP, fluticasone propionate; MF, Mometasone furoate; TAA, Triamcinolone acetonide; PLAC, placebo.

NA, not applicable; PAR, perennial allergic rhinitis; SAR seasonal allergic rhinitis; SD, standard deviation; SUCRA, surface under the cumulative ranking; TNSS, total nasal symptom score; TOSS, total ocular symptom score.
Bold indicates statistical significance (P < .05).

Supplementary Table S8. Pairwise meta-analysis of standardized mean difference (95% CI) for continuous outcomes

Comparisons	No. of studies	Total no. of treatment 1	Total no. of treatment 2	Pairwise meta-analysis SMD (95% CI)	Heterogeneity I^2 (variation in SMD attributable to heterogeneity)
**TNSS changes from baseline in SAR**					
Ciclesonide					
Placebo	1	164	163	-0.44 (-0.66, -0.22)	NA
Fluticasone furoate					
Placebo	6	1127	1054	-0.46 (-0.62,-0.30)	73.71%
Fluticasone propionate					
Placebo	1	144	72	-0.50 (-0.78, -0.21)	NA
Fluticasone furoate	1	144	147	0.10 (-0.13, 0.33)	NA
Triamcinolone acetonide	1	147	148	-0.01 (-0.24, 0.22)	NA
Mometasone furoate					
Placebo	5	773	716	-0.48 (-0.59, -0.36)	12.04%
**TNSS changes from baseline in PAR**					
Budesonide					
Placebo	1	107	114	-0.47 (-0.75, -0.19)	NA
Fluticasone propionate	1	10	14	-0.09 (-0.90, 0.72)	NA
Ciclesonide					
Placebo	2	679	455	-0.30 (-0.42, -0.18)	0.00%
Fluticasone furoate					
Placebo	3	460	459	-0.36 (-0.56, -0.16)	58.09%
Mometasone furoate					
Placebo	2	210	200	-0.28 (-0.52, -0.03)	7.02%
Triamcinolone acetonide					
Placebo	2	319	323	-0.32 (-0.61, -0.03)	65.51%
Fluticasone propionate	1	128	128	0.07 (-0.18, 0.31)	NA
**TOSS changes from baseline in SAR**					
Fluticasone furoate					
Placebo	3	444	442	-0.32 (-0.46, -0.19)	0.00%
Fluticasone propionate					
Placebo	1	314	312	-0.24 (-0.40, -0.09)	NA
Mometasone furoate					
Placebo	2	227	213	-0.7 (-1.87, 0.46)	67.52%

CI, confidence interval; NA, not applicable; PAR, perennial allergic rhinitis; SAR, seasonal allergic rhinitis; SMD, standardized mean difference; TNSS, total nasal symptom score; TOSS, total ocular symptom score
*95% confidence intervals of I^2 were only calculable for treatment comparisons that include more than 2 studies.

Supplementary Table S9. Pairwise meta-analysis of odds ratio (95% CI) for acceptability

Comparisons	No. of studies	No. of events/ Total no. of treatment 1	No. of events/ Total no. of treatment 2	Pairwise meta-analysis Odds ratio (95% CI)	Heterogeneity I^2 (variation in OR attributable to heterogeneity)
**Acceptability in SAR**					
Fluticasone furoate					
Placebo	4	34/828	53/834	0.61 (0.26, 1.39)	62.12%
Fluticasone propionate					
Placebo	1	6/117	10/115	0.59 (0.21, 1.68)	NA
Triamcinolone acetonide	2	8/327	10/320	1.27 (0.49, 3.29)	0.00%
Mometasone furoate					
Placebo	3	9.5/551	9/506	0.95 (0.32, 2.81)	4.43%
Ciclesonide					
Placebo	1	21/164	14/163	1.49 (0.73, 3.03)	NA
**Acceptability in PAR**					
Ciclesonide					
Placebo	2	155/679	96/455	0.97 (0.73, 1.29)	0.00%
Fluticasone furoate					
Placebo	3	194/916	96/507	0.92 (0.70, 1.22)	0.00%
Mometasone furoate					
Placebo	2	8.5/210	7.5/201	1.09 (0.40, 2.97)	0.00%
Triamcinolone acetonide					
Placebo	2	23/324	25/328	0.93 (0.52, 1.68)	0.00%
Fluticasone propionate					
Triamcinolone acetonide	1	3/131	1/129	0.34 (0.03, 3.30)	NA
Budesonide	1	0.5/14	0.5/10	1.40 (0.03, 76.67)	NA

CI, confidence interval; OR, odds ratio; PAR, perennial allergic rhinitis; SAR, seasonal allergic rhinitis.
*95% confidence intervals of I^2 were only calculable for treatment comparisons that include more than 2 studies.

Supplementary Table S10. Assessment of inconsistency in network meta-analysis

**(A) Global inconsistency in networks using the "design-by-treatment" interaction model**			
**Network outcome**	**χ2**	**P value for test of global inconsistency**	
TNSS in SAR	0.29	0.589	
TNSS in PAR	0.41	0.5207	

**(B) Loop inconsistency in networks**			
**Closed triangular or quadratic loop of evidence**	**Inconsistency factor (95% CI)**	**P value**	**Loop heterogeneity τ2**
TNSS changes from baseline in SAR			
FF-FP-PLAC	0.136	0.699	0.026
TNSS changes from baseline in PAR			
BUD-FP-PLAC-TAA	0.351	0.448	0.000

BUD, budesonide; FF, fluticasone furoate; FP, fluticasone propionate; TAA, triamcinolone acetonide; PLAC, placebo. CI; confidence interval; PAR, perennial allergic rhinitis; SAR, seasonal allergic rhinitis; TNSS; total nasal symptom score.
*Heterogeneity of the loop cannot be estimated due to insufficient observations and was set equal to 0.

Supplementary Table S11. Summary of the strength of evidence from pairwise meta-analysis and network meta-analysis of placebo-controlled comparison

	Pairwise meta-analysis				Network meta-analysis	GRADE strength of evidence
**Treatment comparison**	**No. of studies included (n)**	**Effect estimate (95% CI)**	**I^2 (P value)**	**τ^2**	**Effect estimate (95% CI)**	
**TNSS changes from baseline in SAR**						
MF vs PLAC	5 (1489)	SMD -0.48 (-0.59, -0.36)	12.04% (0.34)	0.00	SMD -0.47 (-0.63,-0.31)	moderate[d]
FF vs PLAC	5 (2181)	SMD -0.41 (-0.57, -0.25)	68.91% (0.01)	0.03	SMD -0.46 (-0.59,-0.33)	moderate[a]
CIC vs PLAC	1 (327)	SMD -0.44 (-0.66, -0.22)	NA	0.00	SMD -0.44 (-0.75,-0.13)	moderate[c]
FP vs PLAC	1 (216)	SMD -0.50 (-0.78, -0.21)	NA	0.00	SMD -0.42 (-0.67,-0.17)	moderate[c]
TAA vs PLAC	NA	NA	NA	NA	SMD -0.41 (-0.81,-0.00)	NA
**TNSS changes from baseline in PAR**						
BUD vs PLAC	1 (221)	SMD -0.47 (-0.75, -0.19)	NA	0.00	SMD -0.43 (-0.75,-0.11)	Low[b,c]
FF vs PLAC	3 (919)	SMD -0.36 (-0.56, -0.16)	58.09% (0.09)	0.02	SMD -0.36 (-0.53,-0.19)	moderate[c]
TAA vs PLAC	2 (642)	SMD -0.32 (-0.61, -0.03)	65.51% (0.09)	0.03	SMD -0.32 (-0.54,-0.10)	moderate[b]
FP vs PLAC	NA	NA	NA	NA	SMD -0.28 (-0.64,0.08)	NA
CIC vs PLAC	2 (1134)	SMD -0.30 (-0.42, -0.18)	0.00% (0.73)	0.00	SMD -0.29 (-0.48,-0.11)	moderate[d]
MF vs PLAC	2 (410)	SMD -0.28 (-0.52, -0.03)	7.02% (0.3)	0.01	SMD -0.28 (-0.55,-0.01)	moderate[b]
**TOSS changes from baseline in SAR**						
FF vs PLAC	3 (886)	SMD -0.32 (-0.46, -0.19)	0.00% (0.64)	0.00	SMD -0.32 (-0.45,-0.19)	high
MF vs PLAC	2 (440)	SMD -0.70 (-1.87, 0.46)	67.52% (0.08)	0.53	SMD -0.29 (-0.48,-0.10)	very low[a,b,d]
FP vs PLAC	1 (626)	SMD -0.24 (-0.40, -0.09)	NA	0.00	SMD -0.24 (-0.40,-0.09)	moderate[c]
**Acceptability in SAR**						
FF vs PLAC	4 (1662)	OR 0.61 (0.26, 1.39)	62.12% (0.05)	0.42	OR 0.60 (0.27,1.32)	moderate[b]
FP vs PLAC	1 (232)	OR 0.59 (0.21, 1.68)	NA	0.00	OR 0.57 (0.12,2.67)	low[b,c]
TAA vs PLAC	NA	NA	NA	NA	OR 0.74 (0.10,5.39)	NA
MF vs PLAC	3 (1057)	OR 0.95 (0.32, 2.81)	4.43% (0.35)	0.04	OR 0.89 (0.25,3.20)	moderate[b]
CIC vs PLAC	1 (327)	OR 1.49 (0.73, 3.03)	NA	0.00	OR 1.56 (0.41,6.00)	low[b,d]
**Acceptability in PAR**						
FF vs PLAC	3 (1423)	OR 0.92 (0.70, 1.22)	0.00% (0.99)	0.00	OR 0.90 (0.67,1.20)	moderate[d]
TAA vs PLAC	2 (652)	OR 0.93 (0.52, 1.68)	0.00% (0.90)	0.00	OR 0.92 (0.51,1.67)	high
CIC vs PLAC	2 (1134)	OR 0.97 (0.73, 1.29)	0.00% (0.74)	0.00	OR 0.95 (0.71,1.28)	moderate[d]
MF vs Placebo	2 (411)	OR 1.09 (0.40, 2.97)	0.00% (0.69)	0.00	OR 1.09 (0.40,2.98)	moderate[b]
BUD vs PLAC	NA	NA	NA	NA	OR 3.94 (0.04,409.94)	NA
FP vs PLAC	NA	NA	NA	NA	OR 2.77 (0.26,29.10)	NA

BUD, budesonide; CIC, ciclesonide; FF, fluticasone furoate; FP, fluticasone propionate; MF, Mometasone furoate; TAA, triamcinolone acetonide; PLAC, placebo. CI; confidence interval; PAR, perennial allergic rhinitis; SAR, seasonal allergic rhinitis; TNSS; total nasal symptom score; TOSS, total ocular symptom score.

Grading of Recommended Assessment, Development, and Evaluation (GRADE) rating scale interpretation:

High quality: we are very confident that the true effect lies close to the effect estimate. Moderate quality: we are confident that the true effect is likely close to the effect estimate, but there is a possibility to be substantially different. Low quality: our confidence in the effect estimate is limited; the true effect may differ substantially from the effect estimate. Very low quality: we have very little confidence in the effect estimate, so the true effect is likely to differ substantially from the effect estimate.

[a] Inconsistency, significant heterogeneity, or indirectness.

[b] Imprecision.

[c] Publication bias or single study.

[d] Risk of bias.

Supplementary Table S12. Summary of the strength of evidence from pairwise meta-analysis and network meta-analysis of active-controlled comparison

	Pairwise meta-analysis				Network meta-analysis	GRADE strength of evidence
**Treatment comparison**	**No. of studies included (N)**	**Effect estimate (95% CI)**	**I^2 (P value)**	**τ^2**	**Effect estimate (95% CI)**	
**TNSS changes from baseline in SAR**						
FP vs FF	1 (291)	SMD 0.10 (-0.13, 0.33)	NA	0.00	SMD -0.04 (-0.29,0.21)	very low[a,b,c]
FP vs TAA	1 (295)	SMD -0.01 (-0.24, 0.22)	NA	0.00	SMD -0.01 (-0.33,0.31)	low[b,c]
**TNSS changes from baseline in PAR**						
BUD vs FP	1 (24)	SMD -0.09 (-0.90, 0.72)	NA	0.00	SMD -0.13 (-0.50,0.24)	low[b,c]
TAA vs FP	1 (256)	SMD 0.07 (-0.18, 0.31)	NA	0.00	SMD -0.02 (-0.31,0.26)	very low[a,b,c]
**Acceptability in SAR**						
FP vs TAA	2 (647)	OR 1.27 (0.49, 3.29)	0.00% (0.62)	0.00	OR 0.78 (0.30,2.03)	moderate[b]
**Acceptability in PAR**						
FP vs TAA	1 (260)	OR 0.34 (0.03, 3.30)	NA	0.00	OR 0.33 (0.03,3.25)	moderate[c]
FP vs BUD	1 (24)	OR 1.40 (0.03, 76.67)	NA	0.00	OR 1.42 (0.03,78.11)	low[b,c]

BUD, budesonide; FF, fluticasone furoate; FP, fluticasone propionate; TAA, triamcinolone acetonide.
Grading of Recommended Assessment, Development, and Evaluation (GRADE) rating scale interpretation:
High quality: we are very confident that the true effect lies close to the effect estimate. Moderate quality: we are confident that the true effect is likely close to the effect estimate, but there is a possibility to be substantially different. Low quality: our confidence in the effect estimate is limited; the true effect may differ substantially from the effect estimate. Very low quality: we have very little confidence in the effect estimate, so the true effect is likely to differ substantially from the effect estimate.
[a] Inconsistency, significant heterogeneity, or indirectness.
[b] Imprecision.
[c] Publication bias or single study.
[d] Risk of bias.

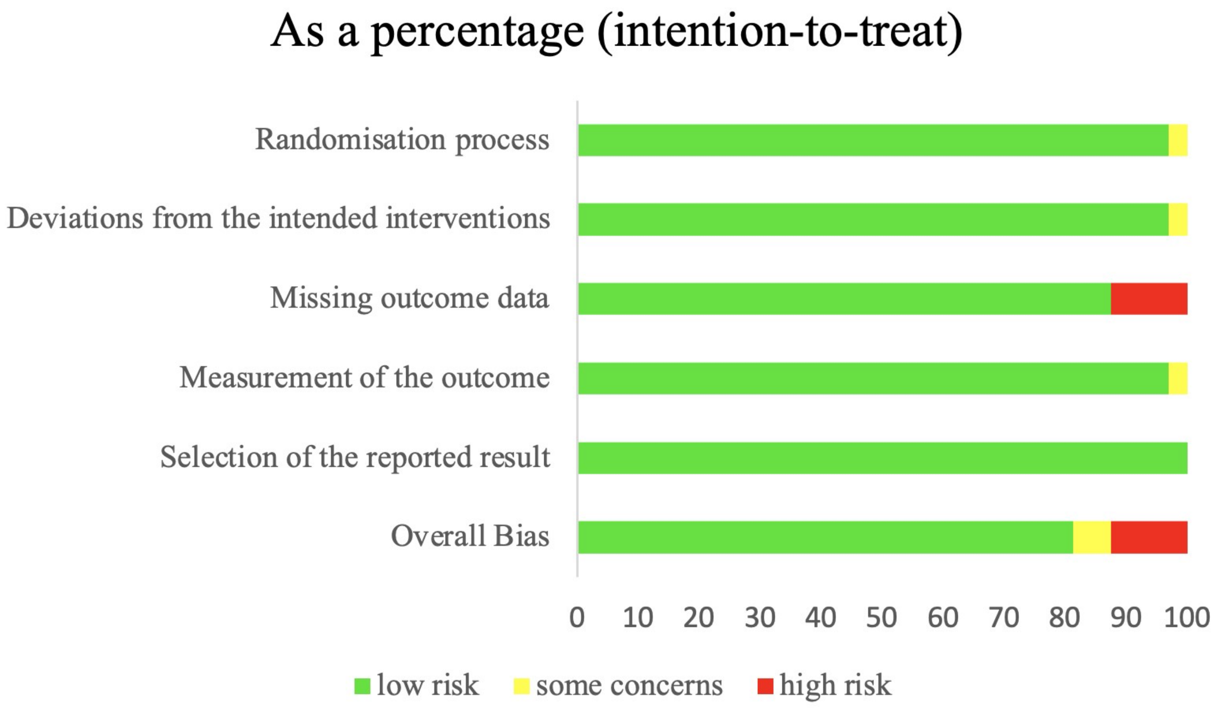

Supplementary Figure S1. Summarized proportions for each domain of the risk-of-bias assessment from included studies.

No.	Study (year)	Randomisation process	Deviations from the intended intervention	Missing outcome data	Measurement of the outcome	Selection of the reported result	Overall
1	Ratner (2015)	+	+	+	+	+	+
2	Igarashi (2012)	+	!	-	+	+	-
3	Meltzer (2011)	+	+	+	+	+	+
4	Prenner (2010)	+	+	+	+	+	+
5	Okubo (2009)	+	+	+	+	+	+
6	Jacobs (2009)	+	+	+	+	+	+
7	Andrews (2009)	+	+	+	+	+	+
8	Kaiser (2007)	+	+	+	+	+	+
9	Fokkens (2007)	+	+	+	+	+	+
10	Ratner (2006)	+	+	+	+	+	+
11	Meltzer (2004)	+	+	+	+	+	+
12	Gawchik (2003)	+	+	+	+	+	+
13	Lumry (2003)	+	+	+	+	+	+
14	Berger (2003)	+	+	+	+	+	+
15	Gross (2002)	+	+	+	+	+	+
16	Meltzer (1998)	+	+	+	+	+	+
17	Bronsky (1996)	+	+	+	+	+	+
18	Bavel (1994)	+	+	+	+	+	+
19	Ratner (1992)	+	+	+	+	+	+
20	Karaulov (2019)	+	+	+	+	+	+
21	Meltzer (2010)	!	+	+	+	+	!
22	Given (2010)	+	+	+	+	+	+
23	Baena-Cagnani (2010)	+	+	+	+	+	+
24	Weinstein (2009)	+	+	+	+	+	+
25	Nathan (2008)	+	+	+	+	+	+
26	Vasar (2008)	+	+	-	+	+	-
27	Meltzer (2007)	+	+	+	+	+	+
28	Chervinsky (2007)	+	+	-	+	+	-
29	Rosenblut (2007)	+	+	-	+	+	-
30	Tai (2003)	+	+	+	!	+	!
31	Fokkens (2002)	+	+	+	+	+	+
32	Kobayashi (1995)	+	+	+	+	+	+

+ Low risk

! Some concerns

- High risk

Supplementary Figure S2. Quality assessment of included studies using the revised Cochrane risk-of-bias tool for randomized trials (RoB 2).

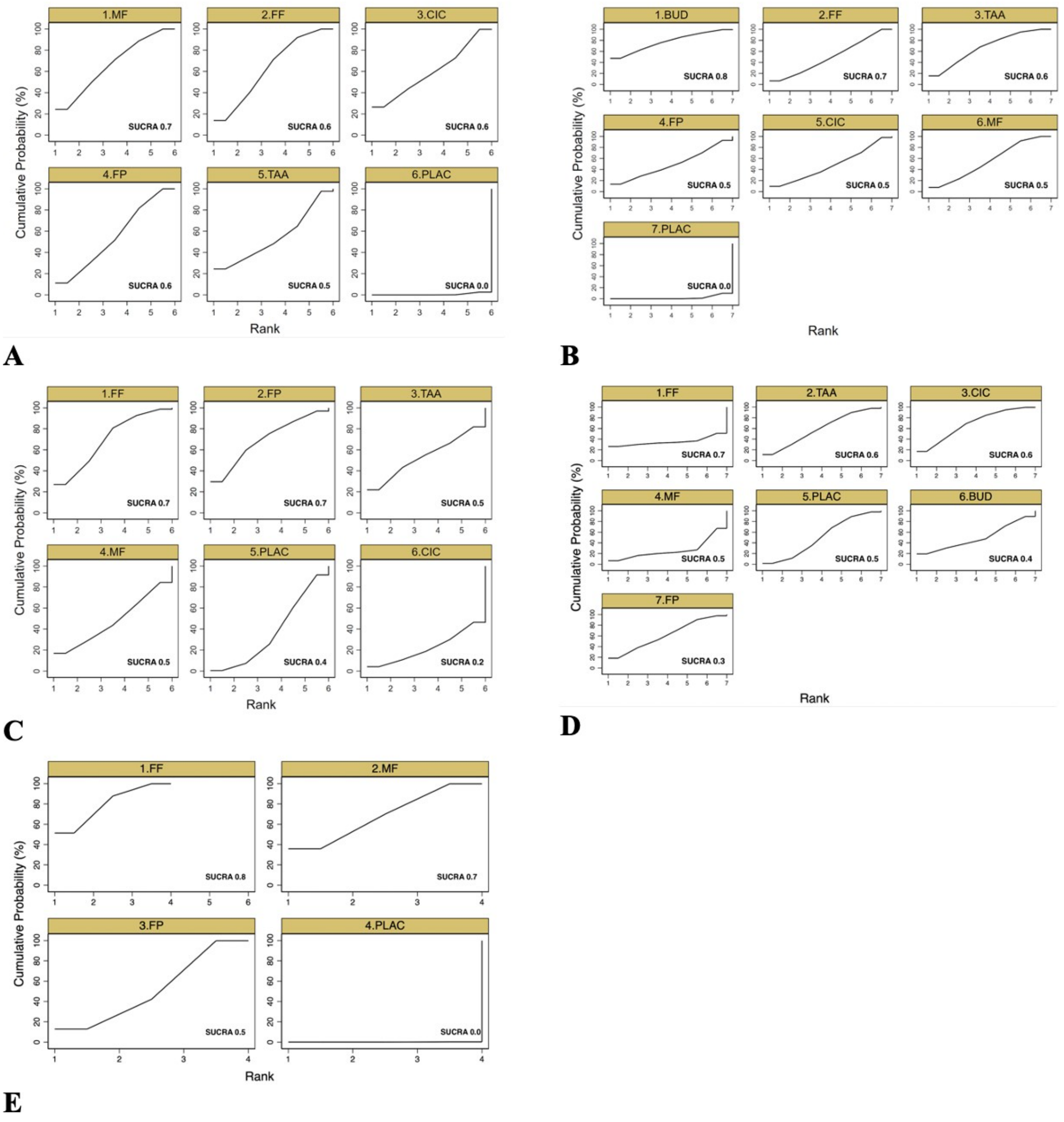

Supplementary Figure S3. The surface under the cumulative ranking (SUCRA) curves according to measured outcomes. **A**, Total nasal symptom score (TNSS) changes from baseline in seasonal allergic rhinitis (SAR); **B**, TNSS changes from baseline in perennial allergic rhinitis (PAR); **C**, Acceptability in SAR; **D**, Acceptability in PAR; **E**, Total ocular symptom score changes from baseline in SAR. BUD, budesonide; CIC, ciclesonide; FF, fluticasone furoate; FP, fluticasone propionate; MF, mometasone furoate; TA, triamcinolone acetonide; PLAC, placebo

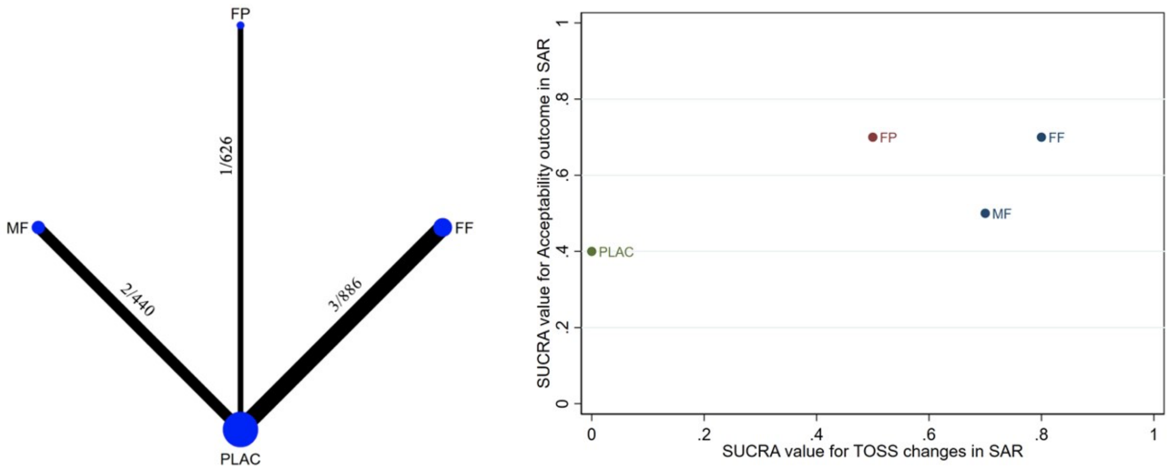

A **B**

Supplementary Figure S4. **A,** Networks of treatment comparisons according to the total ocular symptom score (TOSS) changes from baseline in seasonal allergic rhinitis (SAR), (6 studies, 6 treatment pairs, 1,952 patients). The line thickness is proportional to the number of trials comparing each pair of treatments. The size of every circle is proportional to the number of randomly assigned participants. **B,** Cluster ranking based on SUCRA values for TOSS changes from baseline and acceptability outcome via any cause of dropouts in patients with SAR. FF, fluticasone furoate; FP, fluticasone propionate; MF, mometasone furoate; PLAC, placebo

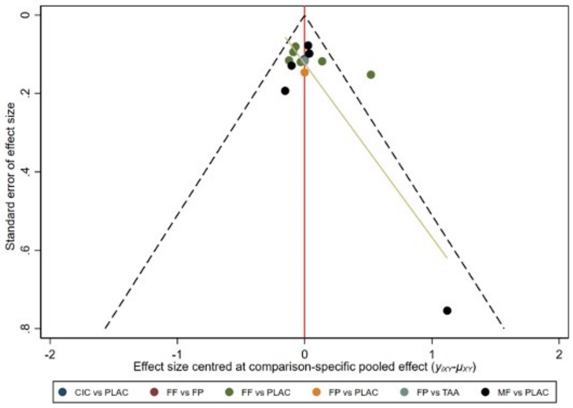
Standard error of effect size
Effect size centred at comparison-specific pooled effect (yiXY-μXY)
CIC vs PLAC
FF vs FP
FF vs PLAC
FP vs PLAC
FP vs TAA
MF vs PLAC

A

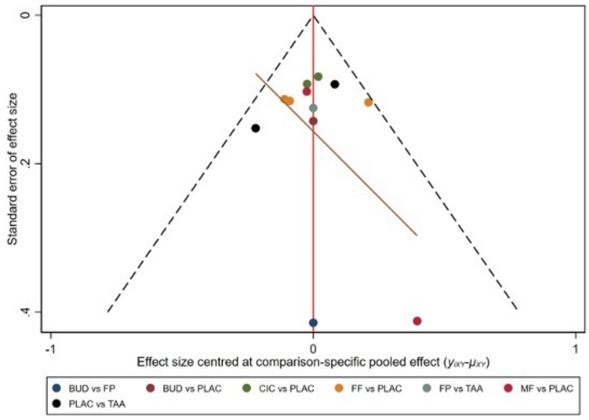
Standard error of effect size
Effect size centred at comparison-specific pooled effect (yiXY-μXY)
BUD vs FP
BUD vs PLAC
CIC vs PLAC
FF vs PLAC
FP vs TAA
MF vs PLAC
PLAC vs TAA

B

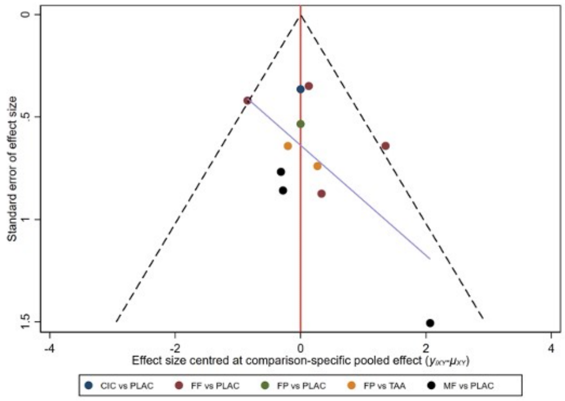
Standard error of effect size
Effect size centred at comparison-specific pooled effect (yiXY-μXY)
CIC vs PLAC
FF vs PLAC
FP vs PLAC
FP vs TAA
MF vs PLAC

C

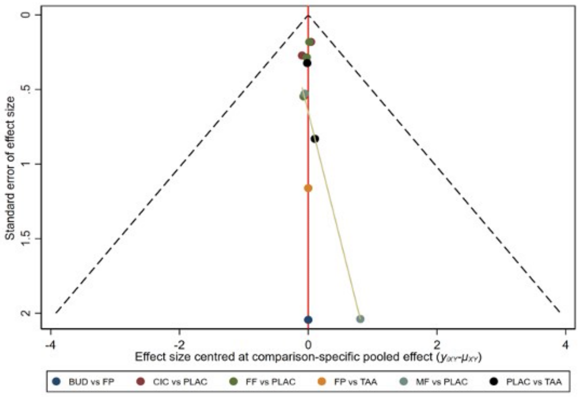
Standard error of effect size
Effect size centred at comparison-specific pooled effect (yiXY-μXY)
BUD vs FP
CIC vs PLAC
FF vs PLAC
FP vs TAA
MF vs PLAC
PLAC vs TAA

D

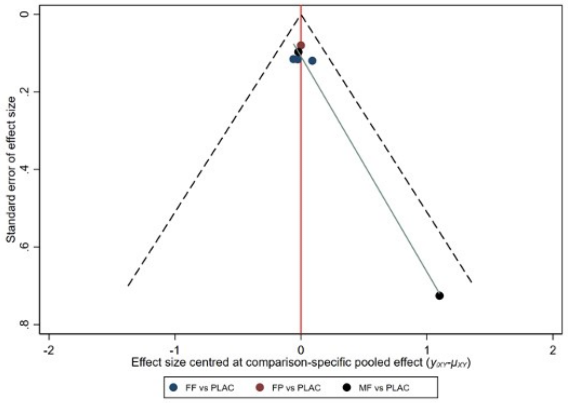
Standard error of effect size
Effect size centred at comparison-specific pooled effect (yiXY-μXY)
FF vs PLAC
FP vs PLAC
MF vs PLAC

E

Supplementary Figure S5. Comparison-adjusted funnel plot for each network. **A,** Total nasal symptom score (TNSS) changes from baseline in seasonal allergic rhinitis (SAR); **B,** TNSS changes from baseline in perennial allergic rhinitis (PAR); **C,** Acceptability in SAR; **D,** Acceptability in PAR; **E,** Total ocular symptom score changes from baseline in SAR. BUD, budesonide; CIC, ciclesonide; FF, fluticasone furoate; FP, fluticasone propionate; MF, mometasone furoate; TA, triamcinolone acetonide; PLAC, placebo
